# Supplementary material for: Identification of genetic variants affecting vitamin D receptor binding and associations with autoimmune disease
Source: Hum Mol Genet. 2017 Mar 9;26(11):2164–76. doi: 10.1093/hmg/ddx092 (PMC5886188; doi:10.1093/hmg/ddx092)
Supplement: Supplementary Data [file ddx092_supp.zip › ddx092-suppl_data/Supplementary_Material_revisions.R2.pdf]

Supplementary Material for
Identification of genetic variants affecting vitamin D receptor binding and associations with autoimmune disease

Giuseppe Gallone[1,2,⋆], Wilfried Haerty[1,2], Giulio Disanto[2], Sreeram Ramagopalan[3,⋆,†], Chris P. Ponting[1,2,4,†], and Antonio J. Berlanga-Taylor[5,6,7⋆,†]

[1]MRC Functional Genomics Unit, University of Oxford, Oxford OX1 3PT
[2]Department of Physiology, Anatomy and Genetics, University of Oxford, South Parks Road, OX1 3PT
[3]Real-World Evidence, Evidera, London, W6 8DL
[4]MRC Human Genetics Unit, The Institute of Genetics and Molecular Medicine, University of Edinburgh, Western General Hospital, Crewe Road, Edinburgh EH4 2XU
[5]Wellcome Trust Centre for Human Genetics, Nuffield Department of Clinical Medicine, University of Oxford, Oxford OX3 7BN
[6]CGAT, MRC Functional Genomics Unit, University of Oxford, Department of Physiology, Anatomy and Genetics, Oxford, Oxford OX1 3PT
[7]*present address:* MRC-PHE Centre for Environment and Health, Department of Epidemiology & Biostatistics, School of Public Health, Faculty of Medicine, Imperial College London, St Mary's Campus, Norfolk Place, LONDON W2 1PG
[†]Authors contributed equally.
[⋆]Corresponding authors.

Contents

1 Study sample **4**

2 Initial Data Processing **4**
2.1 Read alignment to the human genome . . . 4
2.2 Cross-correlation analysis . . . 8
2.3 Genetic variation data and genotype imputation . . . 11

3 General Analytical Methods **12**
3.1 Peak calling . . . 12
3.2 Genomic context analysis . . . 20
3.3 Interval overlap enrichment analyses . . . 22
3.4 Transcription factor binding motif analysis . . . 22
3.5 Functional enrichment at VDR binding locations . . . 24
3.6 Phylogenetic conservation at RXR::VDR motif locations . . . 25
3.7 Definition of Linkage Disequilibrium Intervals . . . 25

4 Detection of Genotype-associated Differential VDR Binding Affinity **26**
4.1 VDR-ASB — Allele-specific Binding Analysis . . . 26
4.2 VDR-QTL — Genotype-Phenotype Bayesian Association Testing . . . 27

5 Analysis of VDR-BVs **30**
5.1 General setup . . . 30
5.2 Analysis of VDR-BV PWM occupancy . . . 30
5.3 VDR-BV Enrichment Analyses . . . 33
5.4 Analyses of VDR-BV Effect in RXR::VDR Consensus Motif . . . 36

6 Supplementary Figures 38

List of Tables

S1	Source of genotype information for the VDR ChIP-exo data	5
S2	Pre-mapping sample statistics	6
S3	Mapping to hg19 reference, statistics	7
S4	Cross-correlation profiles, statistics (ChIP-exo)	11
S5	Cross-correlation profiles, statistics (ChIP-seq)	12
S6	Peak widths, ChIP-exo and ChIP-seq	14
S7	Peak widths, ChIP-exo and ChIP-seq, best 500 regions	15
S8	Peak widths, ChIP-exo and ChIP-seq, only intersecting regions	16
S9	Peak widths, ChIP-exo and ChIP-seq, only IDR reproducible	17
S10	Per-sample consensus peak sets, ChIP-exo	19
S11	Intersection of VDR ChIP-exo and ChIP-seq Binding Regions	20
S12	PScanChIP analysis of genomic regions identified by VDR ChIP-exo peaks	24
S13	QTL genotype-binding affinity association: IMPUTE2/SNPTEST	29
S14	QTL genotype-binding affinity association: IMPUTE2/BIMBAM	29
S15	PScanChIP analysis of PWMs enriched at or around VDR-BVs	31
S16	Ranking of PWM models hit by VDR-BVs	32
S17	VDR-BV enrichment analysis at VitD genes	36
S18	VDR-BVs in bottom (LOB) or top (GOB) deciles of CEU or YRI DAF distributions	37

List of Figures

S1	Cross-correlation profiles	39
S2	Distribution of Peak Widths for the ChIP-exo samples	40
S3	Summary plots for binding affinity data - I	41
S4	Summary plots for binding affinity data - II	42
S5	VDR signal profiles at common genomic features — ChIP-exo vs ChIP-seq	43
S6	VDR ChIP-exo pooled signal profiles at common genomic features	44
S7	Genomic Association Testing	45
S8	Genomic Association Testing — GWAS catalog intervals	46
S9	*De novo* motif analysis at genomic regions identified by VDR ChIP-exo peaks	47
S10	Two classes of VDR binding sites	48
S11	Supplementary GREAT analysis enrichment results	49
S12	Manhattan Plots for the VDR-QTL Bayesian association analysis	50
S13	Sample VDR-QTL event — chr7	51
S14	Sample VDR-QTL event — chr2	52
S15	Sample VDR-QTL event — chr6	53
S16	Genomic association testing of VDR-sBVs/VDR-rBVs with functional annotation	54
S17	Vertebrate conservation of VDR-BVs at bound RXR::VDR motif intervals	55
S18	Class I RXR::VDR motifs — Effects of binding variation on VDR binding affinity	56
S19	Class I RXR::VDR motifs — VDR-BV DAF analysis	57
S20	Distribution of DAFs for VDR-BVs in RXR::VDR motifs	58

1 Study sample

We analysed data generated by ChIP-exo from a total of 30 lymphoblastoid cell lines obtained from Coriell[1]. Of these, a total of 12 (4 trios) were derived from Yoruba individuals from Ibadan, Nigeria, whereas 18 (including 1 trio and 15 unrelated individuals) were derived from individuals of West- and North-European Ancestry who are residents of Utah, USA (CEPH). The analysis in the main text was performed on 27 samples, which passed quality checks (detailed in Section 3.1, Pag. 17).

These cells lines had been genotyped previously as part of the Hapmap project (merged phases I+II and III[2]) [Frazer et al., 2007; Abecasis et al., 2010]. A subset of these has also been genotyped as part of the 1000 genomes project [Abecasis et al., 2012]. Specifically, 23 of the 30 samples have been genotyped on the Omni 2.5 chip platform[3], which has double the number of markers found on any of the single Hapmap chips and uses markers discovered in the 1000 genomes pilot project to a lower allele frequency than were available to Hapmap. Additionally, a total of 16 out of the 30 samples are available as part of the integrated 1000 genomes genotype collection, version 3, 30/4/2012[4].

Of the 30 cell lines available, 19 had been sequenced at low coverage as part of the 1000 genomes project, while for 16 cell lines high coverage full sequencing data is available from Complete Genomics[5]. Table S1, Pag. 5 present a summary of genotype data and full sequencing data currently available.

2 Initial Data Processing

2.1 Read alignment to the human genome

We obtained ChIP-exo sequence reads (single-end 40bp, 1.8+ Phred+33 qualities) from the sequencing facility (Peconics Inc., USA) already aligned against the hg18 reference. Having ascertained that unmapped reads had not been discarded during the hg18 alignment, we extracted raw reads for all samples using the bedtools suite (v. 2.17.0) [Quinlan and Hall, 2010] . The resulting fastq files were used for all remaining analyses. Summary statistics are available in Table S2, Pag. 6.

We mapped the raw sequence reads against the standard hg19 build of the human reference genome using the Stampy aligner (v 1.0.21 [Lunter and Goodson, 2011]), because of its capability of mapping reads containing variable (up to 10-15%) amount of variation with respect to the reference. We followed the recommended procedure and initially mapped raw reads with BWA (v. 0.7.4) [Li and Durbin, 2009] using option `-q 10`, the latter being our choice of fastq quality threshold for read trimming down to 35bp. These bwa-aligned reads represented our input for further alignment using Stampy, with options `--sensitive --bamkeepgoodreads`. Finally, we filtered the reads to retain only uniquely mapping reads with $\mathrm{MAPQ} > 20$. Summary statistics for these alignments are shown in Table S3 (Pag. 7).

Post-mapping QC and removal of blacklisted regions

It is known that functional genomics experiments based on next generation sequencing that measure biochemical activity of various elements in the genome often produce artefactual signal in certain regions of the genome. In order to ensure exclusion of artefactual mapped reads from any of the downstream analyses, we obtained a list of comprehensive empirical blacklists identified by the ENCODE consortium [Dunham et al., 2012] and two additional blacklisted region tracks (DAC, DER) downloaded from the UCSC genome browser[6] [Fujita et al., 2011].

These blacklists are applicable to data based on short-read sequencing (20-100bp) and are available online[7]. They were derived from large compendia of data using a combination of automated heuristics

[1] https://catalog.coriell.org/

[2] phases I+II (rel #24) and III (hapmap3_r2), http://hapmap.ncbi.nlm.nih.gov/downloads/genotypes/latest/forward/non-redundant/

[3] ftp://ftp.1000genomes.ebi.ac.uk/vol1/ftp/technical/working/20120131_omni_genotypes_and_intensities/

[4] ftp://ftp.1000genomes.ebi.ac.uk/vol1/ftp/release/20110521/

[5] completegenomics.com/public-data/69-Genomes/

[6] http://hgwdev.cse.ucsc.edu/cgi-bin/hgFileUi?db=hg19&g=wgEncodeMapability

[7] https://sites.google.com/site/anshulkundaje/projects/blacklists

Family	Sample	Genotypes			Genome	
		1kg_v37	Omni_2.5	Hapmap_R27	1KG	CG (accession)
			**CEU**			
1328	GM06989	✓	✓	✓	✓	✓ (SRR820105)
1340	GM07029			✓		✓ (SRR800229)
1408	GM10831			✓		✓ (SRR800245)
1334	GM10846			✓		
	GM10847	✓	✓	✓	✓	
1350	GM11829	✓	✓	✓	✓	✓ (SRR801804)
	GM11832		✓	✓	✓	✓ (SRR799952)
1423	GM11918		✓	✓	✓	
	GM11919	✓	✓	✓	✓	✓ (SRR801824)
1375	GM12264			✓		
1353	GM12383	✓	✓	✓	✓	
	GM12489	✓	✓	✓	✓	✓ (SRR800011)
1358	GM12716	✓	✓	✓	✓	✓ (SRR825198)
1447	GM12752			✓		✓ (SRR800266)
1459	GM12872	✓	✓	✓	✓	✓ (SRR800101)
13291	GM06986	✓	✓	✓	✓	
	GM06997			✓		
	GM07045			✓		
			**YRI**			
Y110	GM19213	✓	✓	✓	✓	
	GM19215		✓	✓		
	GM19214		✓	✓	✓	
Y111	GM19189	✓	✓	✓	✓	✓ (SRR822866)*
	GM19191		✓	✓		✓ (SRR833574)*
	GM19190	✓	✓	✓	✓	✓ (SRR832868)*
Y116	GM19235	✓	✓	✓	✓	
	GM19237		✓	✓		
	GM19236	✓	✓	✓	✓	✓ (SRR822868)*
Y120	GM19247	✓	✓	✓	✓	✓ (SRR825205)*
	GM19249		✓	✓		✓ (SRR833565)*
	GM19248	✓	✓	✓	✓	

Table S1: Source of genotype information for the VDR ChIP-exo data

**Sample**	**Pop.**	**Family**	**Gender**	**Relationship**	**Total Raw Reads**	**Duplicate Raw Reads** (% Total)
06986	CEU	13291	M	mgf	6,908,366	2,573,780 (37.25)
06989	CEU	1328	F	m	7,097,740	2,521,598 (35.53)
06997	CEU	13291	F	m	5,683,226	2,112,717 (37.17)
07029	CEU	1340	M	f	16,181,109	6,005,207 (37.11)
07045	CEU	13291	F	mgm	9,121,447	3,260,545 (35.74)
10831	CEU	1408	F	m	5,932,335	2,226,465 (37.53)
10846	CEU	1334	M	f	1,378,896	485,865 (35.23)
10847	CEU	1334	F	m	12,771,932	4,737,271 (37.09)
11829	CEU	1350	M	pgf	17,995,364	6,726,654 (37.38)
11832	CEU	1350	F	mgm	10,221,763	3,740,216 (36.60)
11918	CEU	1423	F	pgm	15,744,407	5,918,521 (37.59)
11919	CEU	1423	M	mgf	3,010,139	1,053,686 (35.00)
12264	CEU	1375	M	mgf	5,106,994	1,911,837 (37.43)
12383	CEU	1353	F	pgm	18,063,867	6,560,963 (36.32)
12489	CEU	1353	F	mgm	13,163,810	4,943,935 (37.56)
12716	CEU	1358	M	pgf	5,260,229	1,931,268 (36.71)
12752	CEU	1447	M	f	2,615,550	917,540 (35.08)
12872	CEU	1459	M	pgf	18,497,563	6,764,766 (36.57)
19189	YRI	Y111	M	f	32,380,566	11,973,815 (36.98)
19190	YRI	Y111	F	m	25,110,086	9,523,796 (37.93)
19191	YRI	Y111	M	c	21,021,964	6,492,563 (30.88)
19213	YRI	Y110	M	f	63,178,358	23,917,616 (37.85)
19214	YRI	Y110	F	m	4,303,358	1,138,690 (26.46)
19215	YRI	Y110	F	c	3,790,597	988,175 (26.07)
19235	YRI	Y116	F	m	3,213,974	838,989 (26.10)
19236	YRI	Y116	M	f	10,562,864	3,046,258 (28.84)
19237	YRI	Y116	F	c	10,980,252	3,207,641 (29.21)
19247	YRI	Y120	F	m	12,897,246	3,497,928 (27.12)
19248	YRI	Y120	M	f	17,572,800	4,793,972 (27.28)
19249	YRI	Y120	M	c	35,656,955	10,468,221 (29.36)
μ	–	–	–	–	13,847,459	4,809,350 (34.10)
σ	–	–	–	–	12,655,998	4,662,251 (4.28)

Table S2: Pre-mapping sample statistics. Labels in the `Relationship` column refer to `Family` column coded as follows: *f*: father. *m*: mother. *c*: child. *mgf*: maternal grandfather. *mgm*: maternal grandmother. *pgm*: paternal grandmother. *pgf*: paternal grandfather.

Sample	Pop	Reads				
			Mapped			
		**Total Raw**	BWA (% Total Raw)	Stampy (% Total Raw)	Stampy MAPQ > 20 (% Total Raw)	**Stampy MAPQ > 20 RBL** (% Total Raw)
6986	CEU	6,908,366	6,723,931 (97.33)	6,819,440 (98.71)	6,060,508 (87.72)	5,952,126 (86.16)
6989	CEU	7,097,740	6,908,352 (97.33)	7,006,231 (98.71)	6,199,084 (87.34)	6,108,367 (86.06)
6997	CEU	5,683,226	5,515,548 (97.05)	5,592,955 (98.41)	4,975,834 (87.55)	4,895,473 (86.14)
7029	CEU	16,181,109	15,814,470 (97.73)	16,049,511 (99.19)	14,369,390 (88.80)	14,140,697 (87.39)
7045	CEU	9,121,447	8,850,169 (97.03)	8,987,620 (98.53)	7,978,569 (87.47)	7,833,217 (85.88)
10831	CEU	5,932,335	5,745,533 (96.85)	5,824,324 (98.18)	5,141,689 (86.67)	5,050,573 (85.14)
10846	CEU	1,378,896	1,312,999 (95.22)	1,332,960 (96.67)	1,176,538 (85.32)	1,146,847 (83.17)
10847	CEU	12,771,932	12,271,358 (96.08)	12,459,575 (97.55)	11,053,230 (86.54)	10,867,496 (85.09)
11829	CEU	17,995,364	17,568,702 (97.63)	17,818,928 (99.02)	15,756,822 (87.56)	15,508,527 (86.18)
11832	CEU	10,221,763	9,839,644 (96.26)	9,994,591 (97.78)	8,852,507 (86.60)	8,691,123 (85.02)
11918	CEU	15,744,407	15,357,927 (97.55)	15,564,538 (98.86)	13,899,827 (88.28)	13,732,411 (87.22)
11919	CEU	3,010,139	2,911,243 (96.71)	2,954,462 (98.15)	2,624,087 (87.17)	2,569,979 (85.38)
12264	CEU	5,106,994	4,787,482 (93.74)	4,857,936 (95.12)	4,329,373 (84.77)	4,224,707 (82.72)
12383	CEU	18,063,867	17,533,112 (97.06)	17,786,530 (98.46)	15,717,382 (87.01)	15,441,802 (85.48)
12489	CEU	13,163,810	12,821,201 (97.40)	12,974,455 (98.56)	11,591,799 (88.06)	11,423,271 (86.78)
12716	CEU	5,260,229	5,050,733 (96.02)	5,138,025 (97.68)	4,539,661 (86.30)	4,383,191 (83.33)
12752	CEU	2,615,550	2,531,589 (96.79)	2,567,836 (98.18)	2,280,572 (87.19)	2,236,765 (85.52)
12872	CEU	18,497,563	17,878,779 (96.65)	18,137,005 (98.05)	16,141,183 (87.26)	15,843,545 (85.65)
19189	YRI	32,380,566	31,391,905 (96.95)	31,893,747 (98.50)	28,371,375 (87.62)	27,741,895 (85.67)
19190	YRI	25,110,086	24,436,623 (97.32)	24,848,761 (98.96)	22,089,755 (87.97)	21,647,763 (86.21)
19191	YRI	21,021,964	20,383,012 (96.96)	20,778,384 (98.84)	18,350,477 (87.29)	17,787,029 (84.61)
19213	YRI	63,178,358	61,292,303 (97.01)	62,304,610 (98.62)	54,736,821 (86.64)	53,444,867 (84.59)
19214	YRI	4,303,358	4,140,971 (96.23)	4,219,506 (98.05)	3,719,310 (86.43)	3,597,551 (83.60)
19215	YRI	3,790,597	3,656,947 (96.47)	3,727,320 (98.33)	3,246,813 (85.65)	3,154,452 (83.22)
19235	YRI	3,213,974	3,083,177 (95.93)	3,151,671 (98.06)	2,734,982 (85.09)	2,678,985 (83.35)
19236	YRI	10,562,864	10,183,249 (96.41)	10,396,114 (98.42)	9,082,834 (85.99)	8,872,160 (83.99)
19237	YRI	10,980,252	10,554,731 (96.12)	10,765,451 (98.04)	9,406,748 (85.67)	9,215,655 (83.93)
19247	YRI	12,897,246	12,274,435 (95.17)	12,528,822 (97.14)	10,915,787 (84.64)	10,700,502 (82.97)
19248	YRI	17,572,800	16,001,313 (91.06)	16,326,547 (92.91)	14,194,509 (80.77)	13,836,993 (78.74)
19249	YRI	35,656,955	34,151,403 (95.78)	34,906,583 (97.90)	29,891,373 (83.83)	29,173,224 (81.82)

Table S3: Mapping to `hg19` reference — preliminary BWA alignment, Stampy alignment and final set of useful reads (uniquely mapping, $\mathrm{MAPQ} > 20$), reads in blacklisted regions (UHS, DER, DAC) removed.

and manual curation. Most of the flagged regions are found at specific types of repeats including within centromeres, telomeres and satellite repeats. Here, we removed mapped reads falling in the union of the blacklisted regions prior to any further processing of the data. Final mapping statistics post-removal of reads falling in blacklisted regions are shown in the right-most column of Table S3, Pag. 7.

Treatment of duplicate mapped reads

Historically, ChIP-seq has been performed to build genome-wide maps of binding localisation, without regards to the quantification of the binding signal at the mapped binding positions. In this, the removal of duplicated reads has been considered standard practice[Bailey et al., 2013], because in many instances a high proportion of duplicate reads in ChIP-seq has been linked to PCR amplification bias [Landt et al., 2012]. A formalised measure of the proportion of duplicates in a ChIP-seq dataset has been proposed by the ENCODE guidelines [Landt et al., 2012], which suggest a maximum of 20% duplicates for every 10 million reads sequenced [Landt et al., 2012].

In this study, however, we were interested not only in mapping binding locations, but most importantly in quantifying signal variation in ChIP-exo peaks. Given that we were aiming for a differential binding analysis, the relative magnitudes of the peaks were crucial to the calculations: removing duplicates will diminish the dynamic range such that no peak can have a greater magnitude than the read length. The exclusion of duplicated reads and read pairs from high throughput sequencing data would limit the upper bounds of potential read depth on the genome and therefore would restrict the observable dynamic range of ChIP signal, as discussed in recent work based on the quantification of binding affinity from ChIP-seq data [Ross-Innes et al., 2012; Carroll et al., 2014; Quinodoz et al., 2014]. As a consequence of this, binding affinity analyses of ChIP-seq signals do not discard duplicate reads [Carroll et al., 2014]. Provided the fraction of total duplicate reads is within specifications [Landt et al., 2012], these are usually kept throughout the analysis. Any final differential hypotheses are then manually inspected to identify any completely artefactual results which could be a result of duplicate reads alone.

Here, a further important point to consider is the increased resolution of ChIP-exo as compared to ChIP-seq. Due to the lambda-exonuclease digestion stage, sequencing will initiate, in optimal cases, near to or at the crosslinking site, rather than at the beginning and end of the sonicated fragment. Ideally, a high number of duplicate reads for ChIP-exo are to be expected in the absence of PCR amplification bias, and would be an indication of a successful exonuclease digestion step. Table S2 (Pag. 6) shows our duplication rate varying in the interval 29% to 37%, which we considered within specification for a ChIP-exo experiment [Carroll et al., 2014].

Consequently, for most of the analyses in this study, we decided to either retain all duplicate reads, or to retain duplicate reads based on a binomial distribution of mapping reads and a $p = 1E - 5$ cut-off, following Feng et al. [2012] (for further details, see Section 3.1, Pag. 12).

2.2 Cross-correlation analysis

Sequence reads generated from NGS technologies such as ChIP-seq and ChIP-exo only represent the 5' and 3' portions of the sonicated DNA fragments obtained during the laboratory preparation of the sequencing libraries. In the case of ChIP-seq, this leads to the stereotypical structured pattern of clusters of reads from the Watson and Crick strands. Each pair of such clusters is arranged around the true location of maximal ChIP enrichment.

An important analysis to perform post-alignment of reads is the inference of the approximate consensus size of the sonicated fragments. This is usually performed by evaluating the distances between the Watson and Crick read clusters. This reconstruction of the fragment length from the reads is used for two main purposes:

1. *Peak calling tag shift* — Fragment size estimation can inform a downstream peak calling analysis: a peak caller can take advantage of the inferred fragment length information to accurately reconstruct the peak signal information. To be more specific, let us consider a ChIP-seq dataset mapping the binding patterns of a transcription factor, with read length l_{read} and fragment length l_{fragment} ($l_{\text{read}} < l_{\text{fragment}}$). Many peak callers will either a) *shift* the reads mapped to each strand in the

$5' \rightarrow 3'$ direction for an amount equal to $l_{\text{fragment}}/2$ and pile-up the resulting (+) and (-) tags, or b) *extend* each mapped read in the $5' \rightarrow 3'$ direction into a tag of length l_{fragment}, and pile-up the resulting (+) and (-) tags. Method a) is used, amongst others, by MACS 1.4.2 [Zhang et al., 2008] whereas method b) is used by both MACS 2 [Feng et al., 2012] and Peakranger [Feng et al., 2011].

2. *Data Quality Assessment* — Evaluation of the distribution of fragment sizes estimated from a ChIP-seq dataset may reveal interesting features about the data. The presence of an unambiguous consensus peak in the distribution may indicate that the dataset contains a sufficient number of properly paired peaks (which in turn suggests most peaks represent true biological signal). On the contrary, the absence of a clear consensus fragment distance in the distribution may be due to a number of different reasons, which can be of either technical or biological nature:

   - Sufficient ChIP-seq quality, but few binding sites and hence peaks (relative to the overall size of the genome)
   - Undersequenced dataset (insufficient sequencing depth)
   - Pccurrence of substantial mismapping due to lower read quality
   - Dataset has poor ChIP efficiency (hence lots of background noise and weak peaks)
   - Dataset has broad regions of enrichment and not strong punctate peaks

A relatively robust way of arriving at an estimate of the fragment length $\tilde{d}_{\text{fragment}}$ such that

$$\tilde{d}_{\text{fragment}} \approx l_{\text{fragment}} \tag{S1}$$

is to compute a strand *cross-correlation profile* [Kharchenko et al., 2008] of read start density on the (+) and (-) strand. For each chromosome, we compute the number of read starts at each position on the (+) strand and separately on the (-) strand. Then, we shift these vectors with respect to each other and compute the correlation for each shift d.

We may then plot a cross-correlation profile as the cross-correlation values on the y-axis and the shift d used to compute the correlation on the x-axis. Due to the aforementioned separation of the clustered reads on the (+) and (-) strand around true binding sites, we would then expect to observe a peak in the cross-correlation profile at $\tilde{d}_{\text{fragment}}$ which is predominant in the data. The outcomes of such an analysis may then be broadly divided in the following classes, from best- to worst-case scenario:

- *High quality ChIP-seq dataset* — For a really strong ChIP-seq dataset (excellent antibody and high number of binding regions with respect to the genome), the cross-correlation profile will feature a dominant mode at the true peak shift $d = \tilde{d}_{\text{fragment}}$. In most cases, a much smaller mode will also visible, at $d = l_{\text{read}}$.

- *Medium quality ChIP-seq dataset* — For a moderately strong ChIP-seq dataset (e.g. the TF has few peaks and/or the antibody is not very efficient and/or the peaks are relatively broad scattered peaks) it can be shown that that the cross-correlation profile is noticeably bi-modal, with the first mode at $d = \tilde{d}_{\text{fragment}}$ and another mode at $d = l_{\text{read}}$.

- *Low Quality ChIP-seq dataset* — For a really weak ChIP-seq dataset the cross-correlation profile d will be very different. An extreme situation would be the cross-correlation profile of a control dataset. Here, no discernible mode at $d = \tilde{d}_{\text{fragment}}$ is likely to be present. This would be expected, since in a successful control no significant clustering of reads around specific target sites should be noticeable[8]. However, a peak at $d = l_{\text{read}}$ will most likely be visible even in this case.

In all cases described above we mention the existence of a second mode (or peak) in a ChIP-seq cross-correlation profile for $d = l_{\text{read}}$. This is commonly known as the *phantom peak* [Kundaje et al., 2013].

The phantom peak occurs due to unique mappability properties of the mapped reads. If a position i on the (+) strand in the genome is uniquely mappable, the position $i + l_{\text{read}} - 1$ on the (-) strand is also uniquely mappable. Therefore, in an input dataset (or, equivalently, in a random scattering of reads to uniquely

[8]Except potentially weak biases in open chromatin regions depending on the protocol used.

mappable locations[9]) the odds of finding reads starting on the (+) and (-) strand separated by $d = l_{\text{read}}$ is larger than the odds of finding reads separated by any other shift d.

The relationship between the magnitude of the phantom peak at $d = l_{\text{read}}$ and the presence, position and magnitude of the fragment length peak at $d = l_{\text{fragment}}$ in the cross-correlation profile has been increasingly used as a quality measure for ChIP-seq in a number of recent ENCODE publications [Landt et al., 2012] and has been formalised in two quantitative metrics [Landt et al., 2012; Marinov et al., 2014] known as the Normalized strand cross-correlation coefficient (NSC)

$$\mathbf{NSC} = \frac{\text{cc}\left[\tilde{d}_{\text{fragment}}\right]}{\min\{\text{cc}\}} \tag{S2}$$

and Relative strand cross-correlation coefficient (RSC)

$$\mathbf{RSC} = \frac{\left(\text{cc}\left[\tilde{d}_{\text{fragment}}\right] - \min\{\text{cc}\}\right)}{\left(\text{cc}\left[l_{\text{read}}\right] - \min\{\text{cc}\}\right)} \tag{S3}$$

Cross-correlation analysis with ChIP-exo

The analysis outlined above has been mostly used for ChIP-seq defined analysis of transcription factors. Little has been described about the possibility of performing cross-correlation studies starting from non-punctate binding events or generally broader Chromatin marks. Most importantly, little is known about the applicability of the cross-correlation analysis to ChIP-exo analysis of transcription factor binding. To our knowledge, the only initial assessment on the application of cross-correlation, artefact removal and other ChIP-seq quality metrics to ChIP-exo datasets is presented in Carroll et al. [2014], who carry out preliminary assessments of Estrogen Receptor (ER) and FoxA1 ChIP data.

Crucially, the additional processing stages involved in the preparation of ChIP-exo data imply that the concept of "fragment length" assumes a different meaning. Unlike ChIP-seq, with ChIP-exo, λ-exonuclease digestion is employed to cut the sonicated fragments $5' \to 3'$ on both strands, theoretically down to the TF cross-linking site [Rhee and Pugh, 2011]. Sequencing will then proceed $5' \to 3'$ approximately from the cross-linking site on through the actual binding site on each strand. Therefore, for each binding event, the two clusters of reads from the Watson and Crick strands will not be spaced by $\tilde{d}_{\text{fragment}}$ (as was the case with ChIP-seq) but by a quantity $\tilde{d}_{\text{CL}}$

$$\tilde{d}_{\text{CL}} \approx d_{\text{CL}} = |l_{\text{cl+}} - l_{\text{cl-}}| \tag{S4}$$

where $\tilde{d}_{\text{CL}}$ approximates the distance between the start of the sequencing at the cross-linking site on the (+) and the (-) strand, and crucially, based on what was reported by Rhee and Pugh [2011],

$$d_{\text{CL}} << l_{\text{fragment}}. \tag{S5}$$

As a consequence of this, while a cross-correlation analysis for ChIP-exo data can no more be used to infer $\tilde{d}_{\text{fragment}}$, it can potentially be used to infer $\tilde{d}_{\text{CL}}$.

Unlike the ChIP-seq/TF case, where the l_{read} mostly depends on the library preparation stage and is generally independent of the particular binding features of the TF being considered, in a ChIP-exo scenario d_{CL} will be strongly dependent on the binding configurations for the TF being assayed.

Within the scope of this VDR binding study, the largest binding configuration known is a 15bp double hexamer [Haussler et al., 2013] and $l_{\text{read}} = 40$. As a consequence, any spikes in cross-correlation for d_{CL} will be close to the phantom peak at the read length. However, we deemed it important to assess whether the data would be close to the 1bp resolution [Rhee and Pugh, 2011] and we reasoned that a cross-correlation profile would be a more precise way to visualise this, before looking at meta-models of peak profiles.

Similarly to what Carroll et al. [2014] suggested, for the correlation analyses we used only our sets of uniquely mapping reads where we had removed all data falling in any of the UCSC and ENCODE blacklisted regions. Additionally, we filtered the alignment files to retain only 40bp long mapped reads. This was done due to a small proportion of our reads having $l_{\text{read}} < 40$ due to pre-mapping trimming.

Initially, we calculated cross-correlation profiles for each sample using the phantompeakqual tools[10]

[9] In a genome made up of unmappable, multimappable locations and unique mappable locations

[10] `http://code.google.com/p/phantompeakqualtools/`

**Sample**	**Mapped reads**	$\tilde{d}_{\text{read}}$	$\tilde{d}_{\text{CL}}$ (naive)	$\tilde{d}_{\text{CL}}$	NSC (naive)	NSC
Pooled	351,901,193	40	2 (12)	2 (12,9,22,23)	3.20	7.74
NA19213	54,736,821	39	12	12	3.33	10.56
NA19249	29,891,373	39	39	2	7.06	13.76
NA19189	28,371,375	39	39	2	4.95	17.74
NA19190	22,089,755	40	40	2	5.46	34.01
NA19191	18,350,477	40	40	2	4.66	39.44
NA12872	16,141,183	39	39	12	7.01	11.93
NA11829	15,756,822	39	39	9	5.17	14.63
NA12383	15,717,382	38	38	2	5.61	19.25
NA07029	14,369,390	40	40	2	7.00	24.82
NA19248	14,194,509	39	39	12	9.55	19.70
NA11918	13,899,827	40	40	12	7.74	21.59
NA12489	11,591,799	36	12	12	9.06	15.55
NA10847	11,053,230	37	37	12	6.87	41.59
NA19247	10,915,787	38	38	2	7.51	91.35
NA19237	9,406,748	40	40	12	7.32	6918.11
NA19236	9,082,834	40	40	12	6.58	338.23
NA11832	8,852,507	38	38	9	8.32	5201.03
NA07045	7,978,569	37	31	31	NA	NA
NA06989	6,199,084	41	26	12	8.43	237.08
NA06986	6,060,508	39	39	23	NA	NA
NA10831	5,141,689	39	39	27	NA	NA
NA06997	4,975,834	34	10	10	57.33	181.33
NA12716	4,539,661	40	40	40	NA	NA
NA12264	4,329,373	37	37	36	NA	NA
NA19214	3,719,310	40	40	40	NA	NA
NA19215	3,246,813	41	41	41	NA	NA
NA19235	2,734,982	41	32	32	NA	NA
NA11919	2,624,087	39	39	35	NA	NA
NA12752	2,280,572	35	29	29	NA	NA
NA10846	1,176,538	36	36	36	NA	NA

Table S4: Aggregate statistics for the cross-correlation profiles for the pooled ChIP-exo samples (data also shown by sample, sorted by descending number of mapped reads). Estimated digested fragment lengths where $\tilde{d}_{\text{CL}} > l_{\text{read}}$ have been discarded. Cross-correlation statistics evaluated for $d = [0, 1500]$ nt. Results are reported for both the naive and mappability-corrected analysis. NA indicates cases where an estimate for $\tilde{d}_{\text{CL}}$ could not be obtained, even after correcting for mappability, because the phantom peak dominates the profile.

[Kundaje et al., 2013]. We used a strand shift interval of $[-1500\text{nt}, 1500\text{nt}]$ for all tests. We then further refined these results using a mappability-corrected approach to cross-correlation inference, MaSC [Ramachandran et al., 2013]. Results are presented in Table S4, Pag. 11 and, for the ChIP-seq data, in Table S5, Pag. 12. A summary of the cross-correlation profiling results for the pooled ChIP-exo data is shown in Figure S1, Pag. 39

2.3 Genetic variation data and genotype imputation

To homogenise the available genotype and SNP density information (Section 1, Pag. 4 and Table S1, Pag. 5) across all samples in the study we carried out genotype imputation using the IMPUTE2 platform [Howie et al., 2009, 2011].

We used the 1000 genome reference panel as our reference panel (Phase I-V3-20101123 integrated haplotypes, NCBI build b37, IMPUTE2 data version: June 2014, including genotype likelihoods for 36,820,992 SNPs, 1,384,273 short bi-allelic indels and 14,017 structural variations[11]) and the sparser Hapmap genotype as the study panel. IMPUTE2 was run with default options and a `-int` (chunksize) value of 4Mb.

IMPUTE2 output was post-processed using in-house scripts and post-imputation QC was carried out

[11] `https://mathgen.stats.ox.ac.uk/impute/impute_v2.html#reference`

Sample	Mapped reads	$\tilde{d}_{\text{fragment}}$	$\tilde{d}_{\text{read}}$	NSC
GM10855D3 (Est), R1	10,279,640	126	37	4.458
GM10855D3 (Est), R2	10,276,117	141	36	4.361
GM10855D3, R1	14,510,369	106	37	4.672
GM10855D3, R2	15,469,682	116	38	4.501
GM10855 (Est), R1	13,678,697	124	39	3.293
GM10855 (Est), R2	14,377,146	119	36	3.108
GM10855 (Unstim), R1	14,289,949	122	37	3.453
GM10855 (Unstim), R2	10,540,226	118	38	3.816
GM10861D3 (Est), R1	11,884,378	113	35	4.634
GM10861D3 (Est), R2	12,155,679	116	39	4.473
GM10861D3, R1	12,104,302	121	36	4.397
GM10861D3, R2	13,990,508	111	37	4.214
GM10861 (Est), R1	12,667,408	127	36	3.205
GM10861 (Est), R2	13,517,261	131	37	3.032
GM10861 (Unstim), R1	12,119,343	127	36	3.258
GM10861 (Unstim), R2	8,969,337	127	36	3.589
GM10855-ctrl	13,878,323	128	35	6.005
GM10861-ctrl	14,650,928	126	35	3.983

Table S5: Statistics for the cross-correlation profiles for the ChIP-seq samples in Ramagopalan et al. [2010]. Cross-correlation evaluated for $d = [0, 1500]$ nt. The correlation and NSC statistics refer to the mappability-corrected correlation analysis [Ramachandran et al., 2013].

using QCTOOLS[12]. Variants characterised by parameters `INFO` < 0.4 (for an explanation of this parameter see Marchini and Howie [2010]) and HWE $p < 1 \times 10^{-6}$ were discarded.

3 General Analytical Methods

3.1 Peak calling

We used MACS version 2.0.10 [Feng et al., 2012] and GPS/GEM version 2.4.0 [Guo et al., 2012] to identify regions of VDR binding. The results produced by MACS are, in our experience, quite sensitive to fragment size estimation and read length. MACS estimates a value for $\tilde{d}_{\text{fragment}}$ and then performs tag pile-up and peak calling. The MACS2 fragment size estimation algorithm was not entirely well suited to ChIP-exo data. We deactivated MACS' model estimation routines and employed our own estimation of cross-linked fragment length obtained as explained, via a mappability-corrected cross-correlation analysis [Ramachandran et al., 2013]. MACS is able to use the the custom $\tilde{d}_{\text{CL}}$ chosen to extend each read in the $5' \rightarrow 3'$ direction and to perform read pile-up and peak summit estimation.

Comparison of VDR ChIP-exo and VDR ChIP-seq peak resolution

We used MACS2 to assess the capability of ChIP-exo to identify VDR binding locations at a higher resolution than obtainable with standard ChIP-seq. For the comparison, we downloaded from the NCBI[13] publicly available VDR ChIP-seq data ([Ramagopalan et al., 2010] consisting of data derived from two CEPH lymphoblastoid cell lines from Hapmap, GM10855 and GM10861) . The VDR consensus peakset from Ramagopalan et al. [2010], which was mapped to the hg18 reference, was lifted over to the hg19 reference using the UCSC `liftOver` tool. Additionally, we remapped the original ChIP-seq reads (36nt, single end, Illumina) to the hg19 reference using the same pipeline we applied to the ChIP-exo data, described in Section 2.1.

For the per-sample ChIP-exo MACS 2 peak calling, we only kept reads of length $l_{\text{read}} = 40$nt, meaning we discarded a small proportion of the mapped reads which (due to initial adapter trimming) were of length $l_{\text{read}} < 40$nt. We did this to ensure that the precision of fragment extension and pile-up carried out by

[12]`http://www.well.ox.ac.uk/~gav/qctool`
[13]Gene Expression Omnibus, a.n, GSE22484 and GSE22176, `http://www.ncbi.nlm.nih.gov/geo`

MACS was not unduly influenced by a small percentage of uniquely mapping reads of smaller than expected size. We did include all reads afterwards, during signal quantification.

In order to maximise the resolution potential offered by ChIP-exo, we ran two separate instances of MACS 2 for each sample. In the first, we chose $\tilde{d}_{\mathrm{CL}} = 12$; in the second, we chose $\tilde{d}_{\mathrm{CL}} = 24$ (see Section 2.2) For the peak calling, we employed the "auto" MACS 2 option[14] and a q-value cut-off of $q = 0.1$. For the λ background calculation, we used an estimated effective genome size of 2,540,757,438bp (unique mappability for 40bp Human reads).

Our final selection of per-sample MACS 2 peaksets was obtained by merging, for each sample, the set of intervals obtained for $\tilde{d}_{\mathrm{CL}} = 12$ with the set of intervals obtained with $\tilde{d}_{\mathrm{CL}} = 24$, as follows:

1. *Peak is observed in only one of the two sets* — Peaks in this category are likely to be composed of reads spatially distributed such that most reads on strand (+) are placed at or close to the inferred cross-linked fragment length ($\tilde{d}_{\mathrm{CL}} = 12$ or $\tilde{d}_{\mathrm{CL}} = 24$) with respect to reads on strand (-). We save this peak in the final set as is.

2. *Peak is present in both sets* — Peaks in this category are likely to be composed of reads spatially distributed such that reads on strand (+) are placed at or close to any of the two inferred cross-linked fragment length with respect to reads on strand (-). As a consequence, MACS will detect the peak both when reads are extended by $\tilde{d}_{\mathrm{CL}} = 12$ and when reads are extended by $\tilde{d}_{\mathrm{CL}} = 24$. For this kind of peak, we chose to report the interval width from the $\tilde{d}_{\mathrm{CL}} = 24$ set.

Peak sets obtained according to this procedure were compared to the consensus ChIP-seq peakset from Ramagopalan et al. [2010]. In addition, we included in the comparison peak calling results from ChIP-seq sample GM10855 (calcitriol activated, replicate number 2), again from Ramagopalan et al. [2010], which we analysed using our ChIP-exo pipeline (in summary, reads were mapped to hg19, filtered for mapping quality, removed when found to be mapped to any of the blacklisted regions; peak calling was carried out using the fragment size inferred from the cross-correlation analysis in Section 2.2, $\tilde{d}_{\mathrm{fragment}} = 116$; other parameters as in ChIP-exo analysis). Peak width statistics for the resulting interval sets are shown in Table S6 (Pag. 14, also summarised in Figure S2-A, Pag. 40). The variable number of peaks across samples illustrates the issues with p-value/FDR based thresholding of peak calling analyses on multiple samples characterised by variable numbers of mapping reads.

To identify a more meaningful subset of peaks to compare, we initially thresholded the peaksets to select the best 500 peaks, as ranked by MACS 2 p-value[15]. For the ChIP-exo peaks, the best 500 peaks were selected from each of the two pools ($\tilde{d}_{\mathrm{CL}} = 12$ and $\tilde{d}_{\mathrm{CL}} = 24$) and then pooled as described above. These were compared with the best 500 peaks from the new analysis on the GM10855 sample. The results are in Table S7 (Pag. 15, also summarised in Figure S2-B, Pag. 40).

To verify whether previously identified VDR binding locations could now be picked at higher resolution, we then only selected ChIP-exo peaks intersecting with the consensus ChIP-seq set of 2,776 binding locations in Ramagopalan et al. [2010]. Results are in Table S8 (Pag. 16, also summarised in Figure S2-C, Pag. 40).

Defining a stringent reproducible peakset — the Irreproducible Discovery Rate (IDR)

To address the issue of the difficulty of comparing p-values across datasets we tested the reproducibility of our peaks using the Irreproducible Discovery Rate (IDR) methodology [Li et al., 2011; Landt et al., 2012].

In summary, the IDR is an approach to quantitatively measure the reproducibility of findings identified from replicate experiments and provides highly stable thresholds based on order statistics. The procedure measures the extent to which the ranks of the signals derived from the different samples (ideally biological or technical replicates) are no longer consistent across replicates, in decreasing significance. The IDR approach has been developed as part of the ENCODE and modENCODE projects [Dunham et al., 2012] and lies at the core of ENCODE's standardised framework to obtain uniform peak calls across replicates and samples.

[14] For every position, MACS 2 will decide how many duplicate reads to retain based on a binomial distribution and $p = 1E - 5$ cut-off.

[15] or the maximum number of peaks, whichever was smaller.

**Sample**	**# Peaks**	**Min**	**1st Qu.**	**Median**	**Mean**	**3rd Qu.**	**Max**
GM06986	5,896	12	12	12	24.71	28.00	345
GM06989	704	12	24	40	52.59	64.00	332
GM06997	16,048	12	24	24	42.69	50.00	667
GM07029	3,441	12	24	31	47.33	57.00	469
GM07045	841	12	24	37	47.83	60.00	323
GM10831	343	12	24	36	47.77	61.50	272
GM10846	341	12	24	24	28.80	31.00	145
GM10847	2,356	12	24	39	50.15	64.00	437
GM11829	3,713	12	24	37	50.07	62.00	798
GM11832	1,525	12	24	39	48.09	59.00	393
GM11918	2,688	12	24	42	60.32	78.00	730
GM11919	2,055	12	24	24	30.27	25.50	323
GM12264	5,925	12	24	24	32.05	29.00	389
GM12383	3,670	12	24	38	51.42	66.00	718
GM12489	5,269	12	24	46	62.68	81.00	824
GM12716	242	12	24	36	47.91	59.75	355
GM12752	2,060	12	24	24	35.30	39.00	372
GM12872	6,410	12	24	41	59.97	73.00	802
GM19189	7,751	12	24	36	47.98	61.00	727
GM19190	11,685	12	24	35	45.28	57.00	493
GM19191	2,050	12	13	31	42.50	54.00	390
GM19213	3,851	12	27	45	57.87	74.00	663
GM19214	2,285	12	12	12	22.07	24.00	264
GM19215	2,083	12	12	12	24.69	29.00	321
GM19235	1,173	12	12	12	21.31	24.00	299
GM19236	1,375	12	24	32	43.49	54.00	395
GM19237	1,970	12	24	35	44.15	55.00	405
GM19247	3,291	12	24	42	52.92	68.00	404
GM19248	13,282	12	24	44	59.13	75.00	738
GM19249	50,869	12	24	49	70.40	91.00	740
ChIP-seq (GM10855,D3,R2)	16,098	116	136	176	219.60	246.00	9,030
**ChIP-seq (consensus)**	2,776	100	479	635.50	781.30	891.20	9,980

Table S6: Peak widths, pooled 12/24 dataset and ChIP-seq. $q = 0.1$, MACS 2 `keep-dup=auto`.

Sample	# Peaks	Min	1st Qu.	Median	Mean	3rd Qu.	Max
GM06986	609	12	35.00	58.00	64.17	84.00	345
GM06989	539	12	27.00	43.00	56.84	71.50	332
GM06997	583	16	96.00	147.00	148.20	195.50	667
GM07029	581	12	48.00	94.00	96.29	129.00	469
GM07045	552	12	25.00	40.50	53.22	66.00	323
GM10831	343	12	24.00	36.00	47.77	61.50	272
GM10846	341	12	24.00	24.00	28.80	31.00	145
GM10847	597	12	40.00	66.00	74.03	99.00	437
GM11829	588	13	50.00	81.00	92.44	124.00	798
GM11832	598	12	35.00	49.00	58.46	76.75	393
GM11918	557	13	76.00	114.00	119.60	154.00	730
GM11919	697	12	18.00	24.00	35.49	42.00	323
GM12264	624	12	36.00	56.00	67.60	92.00	389
GM12383	613	12	54.00	81.00	90.07	117.00	718
GM12489	582	13	83.00	126.00	130.60	165.00	824
GM12716	242	12	24.00	36.00	47.91	59.75	355
GM12752	635	12	24.00	39.00	52.43	71.00	372
GM12872	581	14	94.00	143.00	148.20	190.00	802
GM19189	594	14	74.00	111.00	114.30	148.00	727
GM19190	637	12	39.00	72.00	80.06	114.00	493
GM19191	622	12	30.25	53.00	65.94	91.00	390
GM19213	591	13	65.00	102.00	107.00	138.00	663
GM19214	685	12	12.00	24.00	38.24	52.00	264
GM19215	708	12	12.00	30.00	40.89	55.00	321
GM19235	525	12	12.00	13.00	28.92	37.00	299
GM19236	605	12	27.00	41.00	52.30	68.00	395
GM19237	635	12	27.00	44.00	54.14	70.00	405
GM19247	598	12	48.00	79.00	86.36	115.80	404
GM19248	584	17	97.00	136.50	143.90	179.00	738
GM19249	602	20	132.00	184.00	191.00	239.00	740
ChIP-seq (GM10855,D3,R2)	500	187	330	398.50	470.60	515.80	4500

Table S7: Peak Widths, Pooled 12/24 dataset and ChIP-seq. $q = 0.1$, only best 500 Peaks by p-value are shown.

Sample	# Peaks	Min	1st Qu.	Median	Mean	3rd Qu.	Max
GM06986	836	1	19	35.00	45.34	62.00	345
GM06989	298	12	24	38.00	49.47	57.00	332
GM06997	1,366	2	27	57.00	78.00	103.00	667
GM07029	612	12	25	44.00	55.40	68.00	469
GM07045	332	12	24	38.00	48.60	60.00	323
GM10831	127	12	24	32.00	46.94	57.00	272
GM10846	70	12	24	26.00	36.93	42.75	145
GM10847	772	12	34	53.00	61.69	78.00	437
GM11829	889	12	36	59.00	70.80	92.00	798
GM11832	618	8	30	47.00	54.51	66.00	393
GM11918	903	1	33	57.00	67.94	86.00	730
GM11919	385	12	24	27.00	42.31	53.00	323
GM12264	836	9	24	41.00	50.59	64.00	377
GM12383	1,014	11	35	59.00	68.51	89.00	718
GM12489	1,428	12	38	69.00	83.81	115.00	824
GM12716	79	12	24	32.00	48.62	52.50	355
GM12752	511	12	24	30.00	42.34	50.00	372
GM12872	1,588	7	37	67.00	84.89	115.00	802
GM19189	1,544	3	37	66.00	78.57	108.00	727
GM19190	1,418	2	28	50.50	60.92	79.00	493
GM19191	583	12	26	44.00	53.33	68.50	390
GM19213	1,190	12	40	65.50	76.28	102.00	663
GM19214	381	12	12	24.00	33.85	43.00	264
GM19215	485	12	12	27.00	36.57	47.00	321
GM19235	246	12	12	24.00	32.48	41.00	299
GM19236	432	12	24	41.00	50.45	62.25	395
GM19237	610	12	26	43.00	52.18	66.00	405
GM19247	1,110	12	35	60.50	68.46	89.00	404
GM19248	2,120	3	41	81.00	94.77	132.00	738
GM19249	3,163	1	51	103.00	122.30	176.50	740
**ChIP-seq (consensus)**	2,776	100	479	635.50	781.30	891.20	9,980

Table S8: Peak Widths, Pooled 12/24 dataset and ChIP-seq. $q = 0.1$, only ChIP-exo peaks intersecting with VDR ChIP-seq peaks from Ramagopalan et al. [2010] are shown.

Sample	# Peaks	Min	1st Qu.	Median	Mean	3rd Qu.	Max
GM06986	2,273	12	24.00	38.00	63.80	86.00	806
GM06989	2,181	12	24.00	31.00	50.23	46.00	716
GM06997	2,111	25	92.00	137.00	145.10	187.00	833
GM07029	2,486	12	29.00	44.00	71.91	99.00	734
GM07045	2,268	12	24.00	33.00	47.33	46.00	613
GM10831	2,117	12	24.00	24.00	39.45	40.00	496
GM10846	2,112	12	24.00	24.00	27.82	24.00	234
GM10847	2,444	12	26.00	43.00	70.98	100.20	743
GM11829	2,247	12	41.00	80.00	99.81	145.00	845
GM11832	2,338	12	25.00	41.00	60.51	79.00	545
GM11918	2,377	12	32.00	52.00	90.87	135.00	798
GM11919	2,111	24	24.00	24.00	35.85	36.00	494
GM12264	2,114	12	24.00	32.00	55.70	64.00	421
GM12383	2,429	12	33.00	57.00	87.29	127.00	813
GM12489	2,357	12	54.00	128.00	138.80	198.00	840
GM12716	2,155	12	24.00	24.00	36.81	38.00	501
GM12752	2,111	24	24.00	24.00	44.71	41.00	432
GM12872	2,337	12	48.00	127.00	145.80	211.00	852
GM19189	2,431	12	46.00	113.00	125.20	179.00	760
GM19190	2,590	12	34.00	60.00	85.88	123.00	719
GM19191	2,564	12	28.00	43.00	67.16	90.00	625
GM19213	2,464	12	38.00	93.00	113.60	170.00	750
GM19214	2,123	12	24.00	24.00	41.12	41.00	394
GM19215	2,114	12	24.00	24.00	44.46	44.00	479
GM19235	2,111	24	24.00	24.00	35.77	38.00	391
GM19236	2,467	12	24.00	36.00	49.90	53.50	679
GM19237	2,447	12	24.00	36.00	53.58	60.00	420
GM19247	2,454	12	28.25	47.00	78.61	117.80	657
GM19248	2,298	12	94.00	152.00	160.50	213.80	728
GM19249	2,322	12	153.00	213.00	220.50	279.00	783
**ChIP-seq (consensus)**	2,776	100	479.00	635.50	781.30	891.20	9,980

Table S9: Peak Widths, Pooled 12/24 dataset and ChIP-seq data from Ramagopalan et al. [2010]. Only ChIP-exo peaks at the IDR=0.1 threshold level are shown.

In order to assess ChIP-exo peak width using a robust set of peaks reproducible across samples, we followed the IDR pipeline and called peaks on each sample, and on the pooled data from all samples, using MACS 2 and a very loose threshold ($p = 1E - 3$) as required. All duplicate reads were retained.

Again, we ran two groups of peak calling tests using respectively $\tilde{d}_{\mathrm{CL}} = 12$nt and $\tilde{d}_{\mathrm{CL}} = 24$nt. Then, we ran the IDR pipeline once for each set, for all sample pairs combinations (435×2 tests). At an IDR threshold[16] of 0.1 we obtained a set of 1,488 peaks for $\tilde{d}_{\mathrm{CL}} = 12$nt and 2,111 peaks for $\tilde{d}_{\mathrm{CL}} = 24$nt.

We proceeded to pool the two sets as previously explained, obtaining a final estimate of 2,124 reproducible peaks at IDR$= 0.1$. The peak width distributions for the sample-level IDR-reproducible peaks are shown in Figure S2-D, Pag. 40 (full summary results are provided in Table S9, Pag. 17).

To obtain these sample level peak sets, we thresholded the loose MACS 2 peaks calls used for the IDR pipeline at the 0.1 level, both for the $\tilde{d}_{\mathrm{CL}} = 12$nt and for the $\tilde{d}_{\mathrm{CL}} = 24$nt peak sets. We then merged the 2 peak sets for each samples. We refer to the IDR$= 0.1$ peakset of 2,124 regions as $\mathrm{CP}_{\mathrm{IDR}_{0.1}}$.

Defining a consensus binding set based on peak overlap

Having ascertained that our ChIP-exo data allows the identification of read pile-ups at better spatial resolution compared to the consensus ChIP-seq data in Ramagopalan et al. [2010] (and compared to the results of a new analysis of some of the data from the same publication) we then moved on to defining a consensus binding set for differential binding affinity analysis.

[16]The IDR was conceived to assess reproducibility across biological replicates or technical replicates for a sample. Here, we used it in a slightly different context: we sought to understand what the extent of reproducible signal is *across different LCL samples*. Hence, to account for normal biological variation across samples belonging to different individuals, we used a more permissive IDR cut-off than is normally used for replicate reproducibility.

Using the IDR methodology we have shown that it is possible to derive a conservative consensus peaks set of 2,124 peaks that are reproducible across ChIP-exo samples. However, this peakset will be too stringent for a differential binding analysis, because when considering variation in binding affinity across samples at the same position, the *absence* or *near-absence* of read pile-up in a proportion of the samples may carry valuable information.

Therefore, to derive a loose set of binding locations across samples — including locations where signal is carried only by a minimum proportion of the available samples — we used an *overlap* approach: we grouped per-sample peak calls and called a genomic interval a "binding location" for VDR if a peak at that position had been called for at least x of the 30 samples. We examined 3 cases, at an increasing level of stringency: $x = 3, x = 10, x = 20$.

To build this set, we again considered the per-sample MACS2 peak calls obtained for the ChIP-exo versus ChIP-seq comparison (Table S6, Pag. 14). Additionally, we called per-sample peaks using the GEM peak caller (v. 2.4.1, [Guo et al., 2012]) with recommended ChIP-exo options `--smooth 3 --mrc 20 --s 2,540,757,438`.

Again, as for the MACS peak calling analysis, we let GEM decide how many duplicates to retain per sample based on a binomial model. We note that, while we decided to discard most duplicate reads at the peak calling stage by using binomial models of duplication, we *reintroduced* all duplicate reads for the inferred intervals later, at the signal quantification stage. As discussed in Section 2.1, this decision is grounded on the insight that discarding all duplicate reads in a ChIP signal quantification experiment would greatly reduce the dynamic range of signal variation (as the dynamic range would, for every position, be upper bound by l_{read}) and follows the findings of recently published studies suggesting duplicate reads carry information in ChIP-seq quantification studies [Ross-Innes et al., 2012; Carroll et al., 2014], an insight which is even more applicable to ChIP-exo data.

To obtain a set of comprehensive peak calls we then pooled, for each sample, MACS 2 and GEM peaks as follows:

1. *Peak is reported by MACS 2 or GEM only* — Report the peak as is;

2. *Peak is reported by MACS 2 and GEM* — Report the union of the two peaks in the final set.

Sample-level statistics for the resulting peakset collection are show in Table S10, Pag. 19.

Before proceeding further, we noted the extremely low number of consensus binding regions for two CEPH samples, GM10846 and GM12716. For sample GM10846, very few mapping reads were available (Table S3, Pag. 7), while sample GM12716, while having a sufficient number of mapping reads, seemed to fail to return paired peaks both via the MACS and the GEM algorithm, indicating potential high background noise and/or severe problems with the exonuclease cutting, which might have made it impossible to detect paired pile-ups on the opposite strands using either of the two chosen peak callers. Consequently, we did not consider these two samples for further analysis.

Next, we used DiffBind [Stark and Brown, 2011] to manipulate the 28 per-sample merged peaksets and to obtain a consensus peakset based on an overlap threshold. To account for the difference in numbers of mapping reads across samples, we normalised the read numbers using DiffBind's embedded EdgeR routines, based on the Trimmed Means of M's (TMM) algorithm [Robinson and Oshlack, 2010]. Based on DiffBind's definition of overlap as a 1bp minimum overlap, we obtained a total of 16,563 binding regions overlapping at least 3 of the 28 samples. Aggregate results for binding affinity across the samples for the 16,563 region consensus are shown in Figure S3, Pag. 41. From Figures S3-A and S3-B it appears evident that one of the samples, NA06997, stands out, showing overall poor agreement with the other samples. The sample shows extremely high read depth for a fraction of the consensus peaks when compared to any of the remaining 27 samples (Fig. S5-C) which, taken together, show much better patterns of correlation (for example, NA11918 and NA12872, Fig. S3-D). We thus chose to discard sample NA06997, and recalculated overlap statistics using the remaining 27 samples.

As a result, we obtained a total of 15,509 consensus binding regions overlapping 3 of the 27 samples. Aggregate results are shown in Figure S4(A-C), pag. 42.

We designated this peakset CP_{o_3} (consensus peakset at 3 samples overlap). For comparison we also plotted the binding affinity patterns observed when selecting a more stringent set of binding regions overlapping 10 of the 27 samples (2,329 regions, Figure S4(D-F)) and an extremely stringent set of binding

Sample	MACS	GEM	Consensus
GM06986	5,896	4,083	8,018
GM06989	704	2,214	2,424
GM06997	16,048	9,662	17,179
GM07029	3,441	1,471	3,659
GM07045	841	2,120	2,598
GM10831	343	1,346	1,418
GM10846	341	89	352
GM10847	2,356	1,306	2,785
GM11829	3,713	2,562	4,533
GM11832	1,525	2,136	2,820
GM11918	2,688	1,734	2,804
GM11919	2,055	378	2,088
GM12264	5,925	2,426	6,665
GM12383	3,670	2,261	4,355
GM12489	5,269	5,945	7,483
GM12716	242	478	500
GM12752	2,060	1,248	2,381
GM12872	6,410	7,487	9,562
GM19189	7,751	7,136	10,998
GM19190	11,685	5,709	14,043
GM19191	2,050	1,278	2,542
GM19213	3,851	5,065	6,264
GM19214	2,285	1,623	3,226
GM19215	2,083	1,866	3,143
GM19235	1,173	344	1,254
GM19236	1,375	1,048	1,941
GM19237	1,970	1,139	2,635
GM19247	3,291	2,245	4,030
GM19248	13,282	13,979	18,743
GM19249	50,869	26,608	50,644

Table S10: Per-sample consensus peak sets for quantitative ChIP-exo binding affinity analysis. Values in the row termed “consensus” were obtained by merging MACS and GEM calls and fusing intervals at most 20bp away from each other.

Bkg.	Intervals	Obs.	Exp.	Fold	p-value
ChIP-exo ∩ ChIP-seq [Ramagopalan et al., 2010]					
B1	CP_{o_3}	2,118	41.60	49.74	1E-04
B1	$CP_{o_{10}}$	1,029	6.70	133.74	1E-04
B1	$CP_{o_{20}}$	387	2.37	115.13	1E-04
B1	$CP_{IDR_{0.1}}$	868	6.42	117.06	1E-04
B2	CP_{o_3}	2,118	391.46	5.40	1E-04
B2	$CP_{o_{10}}$	1,029	172.52	5.93	1E-04
B2	$CP_{o_{20}}$	387	69.70	5.49	1E-04
B2	$CP_{IDR_{0.1}}$	868	166.45	5.19	1E-04
B3	CP_{o_3}	1,460	38.18	37.29	1E-04
B3	$CP_{o_{10}}$	676	5.98	96.94	1E-04
B3	$CP_{o_{20}}$	239	2.20	75.00	1E-04
B3	$CP_{IDR_{0.1}}$	549	5.61	83.18	1E-04
ChIP-exo ∩ ChIP-seq (GM10855D3-R2)					
B1	CP_{o_3}	6,752	105.07	63.66	1E-04
B1	$CP_{o_{10}}$	1,820	17.18	100.18	1E-04
B1	$CP_{o_{20}}$	572	5.92	82.78	1E-04
B3	CP_{o_3}	4,849	97.94	49.02	1E-04
B3	$CP_{o_{10}}$	1,249	15.41	76.19	1E-04
B3	$CP_{o_{20}}$	397	5.47	61.47	1E-04

Table S11: Intersection of VDR ChIP-exo binding intervals and two interval sets: 1) the 2,776 consensus VDR binding regions from Ramagopalan et al. [2010]; 2) intervals from the ChIP-seq sample GM10855 (calcitriol activated, replicate number 2), from Ramagopalan et al. [2010] — peak called *de novo* using ChIP-exo pipeline.

regions, each overlapping 20/27 samples (732 regions, Figure S4(G-I)). We shall refer to these two peaksets as $CP_{o_{10}}$ and $CP_{o_{20}}$ respectively.

Intersection of VDR ChIP-exo and ChIP-seq binding regions

We tested the intersection of VDR ChIP-exo consensus peaks and ChIP-seq peaks (2,776 consensus regions, [Ramagopalan et al., 2010]) using the Genomic Association Tester [Heger et al., 2013]. All tests used mappability corrected (uniquely mappable 40bp reads) and GC-corrected full segment overlaps. We considered enrichment of peak overlap based on 10,000 randomisations over the following three backgrounds:

- **B1** — full background of ungapped hg19 contigs.
- **B2** — ChIP-seq peaks from Ramagopalan et al. [2010] extended +/-5kb.
- **B3** — Ensembl 73 protein coding genes -5kb upstream TSS and +1kb downstream TTS.

Results are summarised in Table S11.

Generation of signal tracks

We generated signal tracks with MACS version 2.0.10 [Feng et al., 2012]) with options `-g 2540757438 --keep-dup=all --nomodel --extsize 40 -B --SPMR`. We converted MACS2 bedgraph signal tracks to bigwig using the UCSC utilities and prepared them for visualisation in the UCSC browser using custom code.

3.2 Genomic context analysis

We used ngsplot, version 2.41.3 [Shen et al., 2014], to obtain summary visualisations of ChIP-exo signal on and around genomic features. We initially looked at patterns of ChIP-exo signal as related to all Ensembl 75

genes [Flicek et al., 2014]. Successively, we restricted the signal analysis to regions of the genome in close proximity to the consensus peaks called in Section 3.1. To see whether patterns of signal enrichment would be revealed by restricting the analysis to regions in proximity of increasingly stronger peaks, we used the three peaksets derived from the overlap analysis: CP_{o_3}, $\mathrm{CP}_{o_{10}}$ and $\mathrm{CP}_{o_{20}}$.

To compare this data with the findings from Ramagopalan et al. [2010], we included in the study their VDR ChIP-seq consensus peakset (2,776 regions) and the reads for one calcitriol-stimulated sample, GM10855D3 (replicate 2, 15,469,682 uniquely mapped reads to hg19, Table S5). As for the ChIP-exo data, we considered a sample featuring a similar number of mapped reads to GM10855D3, GM19248 (14,194,509 uniquely mapped reads, Table S4). For comparison, we repeated the analysis using the set of pooled reads from all the 27 ChIP-exo samples.

To assign each peak from our consensus peakset to its proximal genomic feature we used the region annotator in DiffRep [Shen et al., 2013]. The region annotator algorithm used Ensembl 75 [Flicek et al., 2014] gene/transcript annotation to assign each peak to one of the following classes:

1. `ProximalPromoter`: +/- 250bp of TSS
2. `Promoter1K`: +/- 1kbp of TSS
3. `Promoter3k`: +/- 3kbp of TSS
4. `Genebody`: anywhere between a feature's promoter and up to 1kbp downstream of the TES
5. `Genedeserts`: genomic regions that are depleted with features and are at least 1Mbp long
6. `Pericentromere`: similarly defined as pericentromere
7. `Subtelomere`: similarly defined as pericentromere.
8. `OtherIntergenic`: any region that does not belong to the above categories

For all of the above categories a "feature" could be any of a) protein coding gene b) pseudogene c) lncRNA d) miRNA. To build the four gene lists based on peak vicinity to proximal features, we collected the Ensembl IDs assigned to each peak in categories 1, 2, 3 and 4 (as cases 5,6,7,8 correspond to peaks falling in regions where a feature cannot be associated).

We ran the ngsplot algorithm for the ChIP-seq sample Gm10855, for the ChIP-exo sample Gm19248 and for the pooled ChIP-exo data (27 samples). For each, we looked at signal pile up at features belonging to the following 5 set:

1. All features
2. Features assigned to CP_{o_3} ChIP-exo consensus peakset;
3. Features assigned to $\mathrm{CP}_{o_{10}}$ ChIP-exo consensus peakset;
4. Features assigned to $\mathrm{CP}_{o_{20}}$ ChIP-exo consensus peakset;
5. Features assigned to ChIP-seq consensus peakset from Ramagopalan et al. [2010];

We computed ChIP-exo signal signatures across a variety of annotations. We used Ensembl 75 [Flicek et al., 2014] and UCSC [Meyer et al., 2013] data for gene body, transcript, exon and CGI (CpG island) information. We relied on ENCODE AWG data [Dunham et al., 2012] (LCL cell line GM12878) for DHS data.

Results are shown in Figure S5 (Pag. 43) and Figure S6.

3.3 Interval overlap enrichment analyses

We used the Genomic Association Tester (GAT) [Heger et al., 2013] to test for enrichment of VDR binding sites in a number of genomic features and disease intervals (Figure S7, Pag. 45). We did this to test whether VDR co-localises with other TFs, whether it binds to known regulatory regions and where it binds in the genome architecture as defined by chromatin segmentation and genomic feature annotation. Additionally, we tested whether we would replicate the significant overlap with disease regions as indicated by GWAS SNPs (GWAS catalog [Welter et al., 2014]) and reported by Ramagopalan et al. [2010].

Based on the four VDR ChIP-exo based peaksets CP_{o_3}, $\mathrm{CP}_{o_{10}}$, $\mathrm{CP}_{o_{20}}$ and $\mathrm{CP}_{\mathrm{IDR}_{0.1}}$, and on the VDR ChIP-seq consensus peakset from Ramagopalan et al. [2010], we conducted the following enrichment overlap tests:

1. Relative enrichment of overlap-based VDR ChIP-exo peaksets, reproducibility-based ChIP-exo peaks and ChIP-seq peaks;
2. Enrichment in common genomic features from (source: Ensembl 72 [Flicek et al., 2014])
3. Each peakset against ENCODE TFBS clusters (V3) uniformly processed by the ENCODE Analysis Working Group[17];
4. Each peakset against ENCODE chromatin segmentation data for LCL sample GM12878[18];
5. Each peakset against DNase clusters (V2) from UW and Duke ENCODE data uniformly processed by the ENCODE Analysis Working Group[19];
6. Each peakset against MHC intervals and extended MHC intervals;
7. Each peakset against FANTOM5 *in vivo* enhancer data from Andersson et al. [2014]
8. Each peakset against intervals obtained by extending the GWAS catalog [Welter et al., 2014] disease SNPs $\pm$ 150kb (as done in Ramagopalan et al. [2010]).

We conducted each binary enrichment test for nucleotide-level overlap of the kind [*VDR peaks*] — [*Test intervals*] based on 10,000 randomisations. For each test, we restricted our background to ungapped hg19 contigs which we further limited to uniquely mappable regions for, respectively, 40bp reads (ChIP-exo) and 36bp (ChIP-seq) (ENCODE mappability tracks were downloaded from the UCSC and processed using in-house scripts) yielding a final mappable genome background of 2,176,142,897bp (36bp ChIP-seq reads) and 2,246,095,071 (40bp ChIP-exo reads). To correct for genome-wide patterns of GC content variability, we produced suitable isochore tracks using in-house scripts and utilised them in the analysis.

3.4 Transcription factor binding motif analysis

For the motif analysis of the sequences underlying the peaks we used a combined approach based on MEME-ChIP [Ma et al., 2014] from the MEME suite [Bailey et al., 2009], XXmotif [Hartmann et al., 2013] and PScanChIP [Zambelli et al., 2013] from the Weeder/MoDtools suite [Pavesi et al., 2006].

De novo motif finding

First, we used MEME-ChIP and XXmotif to perform a *de novo* motif analysis to find unsupervised patterns of motif enrichment. We used the ChIP-exo $\mathrm{CP}_{o_{10}}$ peak set (2,329 regions, 15% of the total number of regions in the CP_{o_3} peak set) for both the *de novo* analyses. For each region in $\mathrm{CP}_{o_{10}}$ we extended each peak summit by $\pm$150bp and extracted the sequence underlying the interval from a repeat-masked version of the hg19 reference, downloaded from the UCSC ftp site [Kuhn et al., 2013].

[17] `http://hgdownload.cse.ucsc.edu/goldenPath/hg19/encodeDCC/wg\-Encode\-Reg\-Tf\-bs\-Cluste\-red/`

[18] `http://hgdownload.cse.ucsc.edu/goldenPath/hg19/encodeDCC/wgEncodeBroadHmm/`

[19] `http://hgdownload.cse.ucsc.edu/goldenPath/hg19/encodeDCC/wgEncodeRegDnaseClustered/`

We ran the following set of MEME-ChIP tools: MEME (to detect longer motifs, i.e. the VDR-RXR heterodimer), DREME [Bailey, 2011] (particularly suited to detecting shorter motif instances, i.e. monomeric VDR binding event) and CENTRIMO [Bailey and Machanick, 2012] (to assess central enrichment for any of the *de novo* motif instances found). Program options were as follows.

```
-meme-mod anr
-meme-minw 5
-meme-maxw 20
-meme-nmotifs 10
-dreme-e 0.05
-centrimo-score 5
-centrimo-ethresh 10
```

Using the same input sequences, we carried out a separate *de novo* search using XXmotif, with the following options:

```
--zoops
--revcomp
--localization
--batch
--merge-motif-threshold LOW
```

Results for the *de novo* motif finding are summarised in Figure S9, Pag. 47. The most enriched motif found by MEME-ChIP (CENTRIMO, E-value $= 7.0e - 115$) almost perfectly matches the consensus motif for the RXR::VDR heterodimer and the one previously found in LCLs by Ramagopalan et al. [2010]. The XXmotif analysis again reports a 15bp PWM closely resembling the consensus RXR::VDR as the most highly enriched (E-value $= 6.36e - 87$) (Figure S9C, sequence logo obtained with WebLogo 3.4 Crooks et al. [2004]).

MEME-ChIP reports motif instances clustered by similarity: Figure S9 reports results for the first two clusters in the ranking, which appear to broadly describe, respectively, mostly heterodimeric and monomeric VDR binding configurations. We note that, in spite of this broad partitioning, cluster 1 (second row) reports a perfect instance of the `(A/G)(A/G)G(T/G)TCA` VDR half site as found by DREME, while cluster 2 mostly reports motifs obtained via assessment of central bias by CENTRIMO. The third and fourth clusters (data not shown) report centrally enriched binding motifs also resembling monomeric/dimeric VDR binding configurations at increasing degree of degeneracy.

PScanChIP Analysis

To obtain an estimate of motif occupancy in the sequence underlying the ChIP-exo peaks, which accounted for a) the specificity of the DNA sequence in the search space and b) any epigenetic priors marking the search space sequences, we ran a PScanChIP analysis [Zambelli et al., 2013] on the full CP_{o_3} peak set. PScanChIP is able to discriminate PWM instances in a set of specified regions based on statistical estimates of *positional bias* within the regions according to different measures and criteria. It provides the option of specifying rather precise backgrounds based on genome-wide collections of putative regulatory (TF accessible) regions in various cell lines. In summary, it outputs PWM enrichment figures at 3 levels:

1. *Global*: whether the PWM is over/under-represented in the search space intervals with respect to a background of DHS TF-accessible regions;

2. *Local*: whether the PWM is over- or under-represented in the 150 bp input regions with respect to the genomic regions flanking them;

3. *Central*: whether the PWM is over-represented at the peak summits.

Additionally, we found that PscanChIP could successfully identify not only the actual binding sites for the TF of interest, but also secondary motifs corresponding to other TFs that tend to bind the same regions, and (if any such TFs are present) provide precise positional correlations among their respective sites.

NAME	ID	**L**_PVALUE O/U	**G**_PVALUE O/U	PREF_POS	PREF_POS_PVALUE
**RXRA::VDR**	MA0074.1	0 ↑	0 ↑	[-3,7]	2.131e-096
**XXmotif15bp**	—	0 ↑	—	[-3,7]	3.049e-124
CTCF	MA0139.1	0 ↑	0.438 ↑	[-6,4]	2.000e-065
**DREME7bp**	—	1.798e-249 ↑	—	[-7,3]	3.814e-157
RORA_1	MA0071.1	7.121e-105 ↑	4.364e-36 ↑	[-5,5]	3.848e-063
GABPA	MA0062.2	1.127e-104 ↑	3.410e-78 ↑	[25,35]	1
RXR::RAR_DR5	MA0159.1	1.781e-88 ↑	2.467e-31 ↑	[-2,8]	2.022e-038
Esrrb	MA0141.1	9.013e-80 ↑	1.984e-20 ↑	[-4,6]	1.132e-038
ELK4	MA0076.1	2.002e-77 ↑	9.960e-68 ↑	[-31,-21]	1
NFYA	MA0060.1	3.395e-70 ↑	8.374e-36 ↑	[-12,-2]	1
NR4A2	MA0160.1	2.114e-68 ↑	3.318e-10 ↑	[-6,4]	2.094e-040
ESR1	MA0112.2	1.479e-65 ↑	1 ↑	[0,10]	4.115e-021
Zfp423	MA0116.1	1.555e-65 ↑	8.754e-05 ↑	[-3,7]	2.947e-020
ELK1	MA0028.1	5.528e-64 ↑	3.481e-70 ↑	[-40,-30]	1
Pax2	MA0067.1	1.395e-63 ↑	1 ↑	[-1,9]	9.728e-008
CREB1	MA0018.2	3.291e-63 ↑	1.871e-18 ↑	[-6,4]	8.567e-015
ESR2	MA0258.1	7.453e-63 ↑	0.061 ↓	[-1,9]	1.549e-024
ARID3A	MA0151.1	1.529e-50 ↓	6.891e-93 ↓	[53,63]	8.476e-008
Mycn	MA0104.2	1.339e-47 ↑	0.018 ↑	[0,10]	3.027e-005
NFE2L1::MafG	MA0089.1	2.673e-47 ↑	1.588e-08 ↓	[-2,8]	1.615e-011

Table S12: PScanChIP analysis of genomic regions identified by VDR ChIP-exo peaks. Results refer to VDR ChIP-exo CP_{o_3} consensus peak set (15,509 regions). Results refers to best 20 ranking PWMs. “XXmotif15bp”: RXR::VDR PWM obtained via *de novo* motif finding using XXmotif. “DREME7bp”: monomeric VDR PWM obtained via *de novo* motif finding using DREME. Other PWMs are from Jaspar. **L**_: ‘local’. **G**_: ‘global’. ↑ indicates over-representation. ↓ indicates under-representation. Results are ordered by local p-value.

We ran PScanChIP using the 15bp PWM derived from the XXmotif *de novo* search and the full PWM collection from the Jaspar Vertebrate database [Mathelier et al., 2014]. The global background for the analysis consisted of DNaseI digital genomic footprinting data for LCL CEPH individual GM12865[20]. Results for this analysis are shown in Table S12, Pag. 24.

The best overall performing PWM is the Jaspar consensus RXR::VDR DR3 motif. It is strongly over-represented locally (in the peak sequences, compared to surrounding regions) and strongly over-represented globally (in the peak regions, compared to DHS sites from CEPH GM12865 background) and significantly centrally enriched. We used the Jaspar RXR::VDR consensus DR3 PWM for all following analyses.

3.5 Functional enrichment at VDR binding locations

We used GREAT [McLean et al., 2010] to assess whether the VDR binding regions in our consensus ChIP-exo peaksets were positionally close to genes enriched for any functional role. We carried out the following enrichment analyses:

1. CP_{o_3} (15,509 intervals)
2. $\mathrm{CP}_{o_{10}}$ (2,329 intervals)
3. Sites containing the top 20% and bottom 20% quantiles (PScanChip Score, Figure S10, Pag. 48) of RXR::VDR DR3 motif instances (15bp PWM, RXR::VDR DR3 Jaspar PWM)

Species, assembly and background were set to GREAT’s defaults (UCSC hg19 and whole genome). For the association rule settings, we used a nearest gene association rule (1000kb window): each gene is assigned a regulatory domain that extends in both directions to the midpoint between the gene’s TSS and the nearest gene’s TSS but no more than the maximum extension in one direction (Figure S11, Pag. 49).

[20]Coriell GM12865, lineage: mesoderm, tissue: blood, from `http://genome.ucsc.edu/ENCODE/protocols/cell/human/GM12865_Stam_protocol.pdf`

3.6 Phylogenetic conservation at RXR::VDR motif locations

We collected PhastCons scores [Siepel et al., 2005] for each nucleotide in the 15bp consensus RXR::VDR motifs within CP_{o_3} regions. RXR::VDR motif intervals were those found via the PScanChIP analysis (RXR::VDR Jaspar consensus, Section 3.4, Pag. 23). We tested both vertebrate and primate PhastCons score tracks (hg19) downloaded from the UCSC browser [Kent et al., 2002]. Deciles for the PhastCons score distributions at *class I* VDR binding regions are shown in Figure S17A. We used R [R Core Team, 2014] to carry out all the significance tests. A Kruskal Wallis test revealed a significant ($\alpha = 0.05$) effect of nucleotide position within the motif on PhastCons score, both for vertebrate conservation ($\chi^2(14) = 264.28$, $p < 0.01$) and for primate conservation ($\chi^2(14) = 38.70$, $p < 0.01$). Thus, we proceeded to apply *post-hoc* tests (R package PMCMR, Tukey and Kramer (Nemenyi) test with Chi-square correction) to identify which nucleotide positions pairs show significant differences in their PhastCons score distributions (Figure S17B shows a Hinton plot of significant pairs for class I VDR binding sites).

3.7 Definition of Linkage Disequilibrium Intervals

We defined linkage disequilibrium blocks based on two alternative definitions of linkage disequilibrium: a D'-based and a r^2-based definition.

D'-based definition of LD blocks

We downloaded data for the 1000G Phase I Integrated Release Version 3 Haplotypes (2010-11 data freeze, 2012-03-14 haplotypes) split by population (EUR and AFR panels) from the repository at the Centre for Statistical Genetics, University of Michigan[21] (monomorphic sites removed). This repository did not contain data for chromosome X: we downloaded data for chrX directly from the 1000 genomes main ftp site, partitioned it to obtain data from CEU/YRI cohorts only and merged the resulting entries with autosomal data using in-house scripts.

We pre-processed vcf files with vcf-tools[22] version 0.1.12b and PLINK Purcell [2014] version 1.9b to obtain Plink binary bfiles, with pre-filtering options `--maf 0.002 --hwe 0.001`. We derived haplotype blocks for each of the two populations using PLINK 1.9b [Purcell, 2014] with options

```
 plink
--blocks no-pheno-req
--blocks-max-kb 500
--blocks-strong-lowci 0.7005
```

which produced strong LD blocks based on the D' definition (which has advantages over an r^2 definition, see for example Zapata [2000]); PLINK's implementation of D'-based LD blocks is based on the definition and thresholds in [Gabriel et al., 2002]. CI settings were left at their default settings: Low CI was set 0.7005, High CI was set to 0.98 and recombination CI was set to 0.9. Maximum haploblock span was set to 500 kb.

r^2-based definition of LD blocks

We downloaded pre-computed r^2-based LD blocks provided by the BROAD institute[23] for CEU $r^2 = 1$ and $r^2 > 0.8$. These were obtained based on 1000 genomes SNPs (Phase 1 v. 3) filtered by the developer of BEAGLE[24]. We processed the LD blocks and mapped the sentinel genome-wide significant GWAS SNPs using in-house code.

[21] `www.sph.umich.edu/csg/abecasis/MaCH/download/1000G.2012-03-14.html`
[22] `vcftools.sourceforge.net/`
[23] `data.broadinstitute.org/srlab/BEAGLE/1kG-beagle-release3/ld_intervals/`
[24] `bochet.gcc.biostat.washington.edu/beagle/1000_Genomes_phase3_v5/READ_ME_beagle_ref`

4 Detection of Genotype-associated Differential VDR Binding Affinity

4.1 VDR-ASB — Allele-specific Binding Analysis

To verify if genetic variation under VDR peaks is statistically associated with modulation or disruption of VDR binding affinity signal, we performed an Allele-Specific Binding (ASB) analysis: we utilised the sequence composition of ChIP-exo sequence reads overlapping heterozygous SNPs to determine the sequences originating from each allele separately [McDaniell et al., 2010; Pickrell et al., 2010; Rozowsky et al., 2011] and to identify allele-specific binding events with significant difference in the number of mapped reads between parental alleles.

Background

We employed a modified version of the AlleleSeq pipeline [Rozowsky et al., 2011] to perform an allelic imbalance analysis. We refer to Rozowsky et al. [2011] for details on the pipeline. Briefly, we tested for significant allelic imbalance all read pile-ups intersecting a variant showing heterozygous genotype, given a null hypothesis of 50% paternal versus 50% maternal read distribution. We followed Rozowsky et al. in controlling for two major sources of bias typically encountered when running an allele-specific analysis of NGS short read data: a bias due to mapping to the reference hg19 genome [Degner et al., 2009] and a bias due to unannotated copy number variants skewing the read counts for a proportion of the SNPs being tested [Pickrell et al., 2011].

To correct for the first source of bias (reference mapping bias) we derived, for each LCL sample in our study, two personalised genome sequences, incorporating the genotype data available for the variants in the 1000 genomes project. Following Rozowsky et al. [2011], for each LCL individual, we aligned its ChIP-exo reads to each of its parental reference genomes and determined a best-case alignment based on the characteristics of each read. This resulted in a set of optimal alignments devoid of reference mapping bias.

To correct for the second source of bias (unannotated copy number variants. e.g. regions of genomic duplication or deletion potentially resulting in spurious allelic imbalance hypotheses) we obtained whole genome sequencing data for our study LCL samples and performed de-novo CNV detection using CNVnator [Abyzov et al., 2011]. CNV calls from CNVnator were passed on to the AlleleSeq pipeline, to discard any ASB SNP hypotheses falling into regions characterised by significant copy number variation.

In addition, we corrected for a third source of potential erroneous ASB SNP calls by removing any significant allelic imbalance hypothesis falling under repeat regions contained in the ENCODE blacklist data [Dunham et al., 2012] (Section 2.1, Pag. 4).

Setup

We ran the allele-specific binding analysis on a subset of 20 of the available 27 LCL individuals, conditioned on the simultaneous availability of genotype and whole genome sequence data for each individual considered.

We used 1000 genomes genotyped variants to construct two personalised genomes for each LCL individual using the vcf2diploid tool [Rozowsky et al., 2011]. For the genotype data, 1000 genomes genotyped variants were directly available for 15 of the 20 samples for which whole genome sequence data was available (Table S1, Pag. 5). For the remaining 5 individuals we used available Hapmap variants as a scaffold to carry out imputation from the 1000 genomes reference as explained in Section 2.3, Pag. 11. All variant sets were applied to the 1000 genomes technical reference, `1kg_v37` (b37), and indexes were generated using Bowtie 0.12.9 [Langmead et al., 2009].

To address the unannotated CNV bias, we obtained whole genome sequencing data for 16 LCL individuals from the 1000 genomes ftp site[25] (9 CEU individuals, 7 YRI individuals, 4X coverage). This low coverage whole genome sequencing data was not available through the 1000 genomes project for 14 of the 30 LCL individuals. However, we were able to source high coverage whole genome alignment for 4 of the 14 individuals (NA07029, NA10831, NA19191, NA19249) via 1000 genomes Complete Genomics data[26].

[25] `ftp://ftp.1000genomes.ebi.ac.uk/vol1/ftp/data/`

[26] `http://www.completegenomics.com/public-data/69-Genomes/`

We downloaded CG whole genome sequences in SRA through the NCBI website[27], NCBI SRA accessions SRR800229, SRR800245, SRR833574, SRR833565, 51X average coverage).

We called CNVs on the 20 whole genome alignments using CNVnator [Abyzov et al., 2011] using the recommended bin size of 1000bp for the low coverage alignments and 100bp for the high coverage alignments. We processed the CNV calls for usage in the ASB pipeline via in-house scripts. LCL individuals lacking whole genome alignments were not considered in this study.

We ran one allelic imbalance test for each of the twenty ChIP-exo samples. The Bowtie parameters for the alignments to the parental genomes were set to AlleleSeq defaults (`--best --strata -v 2 -m 1`). We only tested heterozygous SNPs underlying a minimum of 5 uniquely mapping reads, and reported VDR-ASB hypotheses after FDR correction (FDR < 0.1) of the empirical p-values produced by AlleleSeq's randomisation procedure. As vcf2diploid skips variants with different rsID at the same position during the construction of the diploid genomes, we also discarded any such variants from the ASB imbalance results.

4.2 VDR-QTL — Genotype-Phenotype Bayesian Association Testing

The Allele-Specific Binding analysis described above is only able to test variants which are heterozygous for the LCL sample under consideration.

To see whether we could detect variation or disruption of binding affinity at VDR binding regions intersecting variants which would be missed by the ASB approach, we used an approach which is in part orthogonal to the VDR-ASB analysis. For each VDR binding region overlapping a variant, we analysed all the 27 samples at once. We grouped the samples based on the genotypes of the underlying variant, and performed QTL association testing between the variant's genotype (under the assumption of an additive genetic model [Stephens and Balding, 2009]) and the phenotype at that location (the VDR binding affinity based on the ChIP-exo peak size at that location). We used an in-house pipeline based on SNPs and indels imputed with IMPUTE2 [Howie et al., 2009] and Bayesian regression modelling based on SNPTEST [Marchini et al., 2007] and BIMBAM [Servin and Stephens, 2007]. Bayesian methods for analyzing SNP associations are now an established tool in GWAS analysis [Scott et al., 2007; Burton et al., 2007; Wakefield, 2009] and have advantages over the use of p-values in power and interpretation [Stephens and Balding, 2009] although they require tighter initial modelling assumptions when compared to frequentist methods.

Background

We performed imputation of the variation data because we had high coverage 1000 Genomes variants for only a subset of the samples (Table S1, Pag. 5). Imputation of the variation data was carried out as described in Section 2.3 (Pag. 11). The phenotype data consisted of read counts under peak intervals for the CP_{o_3} consensus peak set. Only 27/30 samples were considered for the QTL analysis, following our findings detailed in Section 3.1. Pag. 17.

For the tests, we considered a standard regression model for additive genetic effects with normally distributed error as described by Marchini et al. [2007]

$$\phi'_i = \gamma e_i + \epsilon_i, \qquad \epsilon_i \sim N(0, \sigma^2) \tag{S6}$$

where ϕ'_i is the residual phenotype of the ith individual after a baseline mean and any covariate effects have been estimated and subtracted off, e_i is an additive coding for the thresholded or expected genotype of the ith individual and σ^2 is the error variance. The null model M_0, to which the model M_1 in Equation 4.2 is compared, is

$$\phi'_i = \epsilon_i, \qquad \epsilon_i \sim N(0, \sigma^2). \tag{S7}$$

Ultimately, within a Bayesian framework we are interested in calculating, for each test, a Bayes Factor (BF), which is a ratio of marginal likelihoods between the model of association and the null model

$$BF = \frac{P(\text{data}|M_1)}{P(\text{data}|M_0)}. \tag{S8}$$

[27] `http://trace.ncbi.nlm.nih.gov/Traces/sra`

Setup

The QTL analysis was performed according to SNPTEST recommendations and standard procedures[28]. As regards the prior specification, SNPTEST uses a Normal Inverse Gamma prior on the regression coefficient γ and on the error variance σ^2

$$\gamma|\sigma^2 \sim N(m_\gamma, V_\gamma\sigma^2) \qquad \sigma^2 \sim \text{InverseGamma}(a, b) \tag{S9}$$

where the prior variance on γ is specified in terms of the fraction V_γ of the error variance σ^2. For the analysis, we set $V_\gamma = 0.2, m_\gamma = 0, a = 3, b = 2$. We provided genotypes in binary .bgen IMPUTE2 format (which associates probabilities, rather than hard calls, to imputed genotypes) and employed the `expected` genotype count option to account for genotype uncertainty information from IMPUTE2.

For each test, phenotypes were first mean centred and scaled to unit variance, then quantile normalised. The two covariates *ethnicity* (CEU/YRI) and *gender* (male/female) were estimated and subtracted off ϕ prior to carrying out the regression tests. We only considered imputed variants underlying peak intervals in the CP_{o_3} consensus peaksets. For each peak, we tested each unique pair $(\text{variant}, \text{bindingaffinity})$ individually, using binding affinity signal for all the 27 samples, segregated in classes based on genotype (homozygous reference, heterozygous, homozygous non-reference, coded as 0,1,2 under the assumption of an additive genetic model).

To assess the robustness of the hypotheses generated with IMPUTE2/SNPTEST, we ran another Bayesian regression algorithm, BIMBAM [Servin and Stephens, 2007]. For this analysis, the regression model was as before (Equation 4.2) while priors were as in Servin and Stephens [2007]. Genotype and phenotype sets were as in the previous analysis, and phenotypes were again quantile normalised, this time using the R function `qqnorm`, before testing.

Posterior Probability of Association (PPA)

Having obtained a list of BFs from the genotype-phenotype association, we then considered the problem of computing, for each SNP, the Posterior Probability of Association (PPA), i.e. the probability that it is truly associated with the phenotype (irrespective of power, sample size or how many SNPs were tested). The PPA combines the evidence observed in the data and quantified via the BF with *prior probability* that the SNP is truly associated with a phenotype. Following Stephens and Balding [2009], we can express the PPA in term of the Posterior Odds (PO) on M_1,

$$\text{PPA} = \text{PO}/(1 + \text{PO}) \tag{S10}$$

where PO is defined as

$$\text{PO} = \text{BF} \times \pi/(1 - \pi). \tag{S11}$$

In a Bayesian association context, π is the prior probability of M_1, and can be interpreted as a prior estimate of the overall proportion of SNPs that are truly associated with a phenotype.

If we make the agnostic assumption that 50% of the variants underlying the VDR peak intervals are associated with a binding affinity phenotype and set $\pi = .5$ (that is we set the prior odds to 1:1, which means we assume that, *a priori*, association with genetic variation in the peak regions is considered equally plausible as no association) and we filter the candidates for $\text{PPA} > 50\%$, we obtain the candidates in Table S13 (for the SNPTEST pipeline) and Table S14 (for the BIMBAM pipeline) (Manhattan Plots of SNPTEST and BIMBAM results presented in Supplementary Figure S12.

A total of 4 VDR-QTL loci were identified by both SNPTEST and BIMBAM. Summary results for the event corresponding to SNP rs17160772 (chr7-138,803,023, hg19) are presented in Figure S13, Pag. 51, and further two sample loci (for SNP rs13386439, chr2-212,320,111 and SNP rs178399, chr6-2,791,683) are in Supplementary Figures S14 and S15, respectively.

[28] `https://mathgen.stats.ox.ac.uk/genetics_software/snptest/snptest.html#introduction`

Chr	Max_peak_rd	RsID	Pos.	alleleA	alleleB	AA	AB	BB	$\log_{10}(\mathbf{BF})$	$\mathbf{PPA}(\pi = 0.5)$
8	26.85	rs68025741	110,533,892	G	GA	10.00	10.00	7.00	3.10718	0.76
2	184.26	**rs13386439**	212,320,111	A	T	13.98	9.02	4.00	2.07696	0.68
6	264.51	rs178399	2,791,683	G	A	19.00	5.00	3.00	1.85762	0.65
9	20.75	rs10816673	111,241,335	C	T	16.00	8.00	3.00	1.62579	0.62
8	76.27	**rs6985113**	105,698,914	G	A	5.00	12.00	10.00	1.59276	0.62
16	27.79	rs11150614	31,366,016	A	G	1.00	8.00	18.00	1.42352	0.59
19	44.58	**rs1560707**	10,750,738	T	G	7.00	8.00	12.00	1.38037	0.58
3	147.48	rs7651107	72,035,276	A	C	10.00	11.58	5.42	1.35183	0.58
5	208.66	rs6580323	139,340,779	C	T	2.07	14.49	10.44	1.34747	0.57
1	46.37	rs10800934	203,398,630	G	C	9.00	13.00	5.00	1.29216	0.57
6	28.24	rs17243157	24,667,323	C	T	21.00	6.00	0.00	1.27171	0.56
7	366.73	**rs17160772**	138,803,023	G	C	15.00	11.00	1.00	1.23887	0.56
1	30.57	rs6687177	157,151,799	G	C	9.00	11.00	7.00	1.15258	0.55
1	69.85	rs58934828	245,108,637	A	G	15.13	10.85	1.01	1.14268	0.53
17	23.49	rs733342	80,822,080	A	G	12.00	14.00	1.00	1.10570	0.53
4	31.18	rs6828800	144,293,936	G	A	17.33	7.33	2.33	1.08461	0.52
5	32.10	rs199643317	43,106,026	C	CTG	4.00	12.00	11.00	1.06912	0.52
20	25.29	rs2246735	4,803,494	C	T	5.40	15.61	5.99	1.06315	0.52
7	41.61	rs66841847	64,147,102	T	TGG	14.88	10.12	2.00	1.00171	0.50

Table S13: IMPUTE2/SNPTEST QTL genotype-binding affinity association. Only results at the $log_{10}\text{BF} > 1$ threshold are shown. SNP rsIDs highlighted in bold font intersect with top BIMBAM findings.

Chr	Pos.	RsID	$\log_{10}(\mathbf{BF})$	R_Pval	μ	a	d	$\mathbf{PPA}(\pi = 0.5)$
7	138,803,023	**rs17160772**	1.851	0.0001	-0.189	0.380	0.015	0.65
1	45,187,330	rs11800505	1.652	0.0003	0.224	-0.339	-0.010	0.62
18	19,192,255	rs2850556	1.622	0.0001	-0.260	0.343	0.014	0.62
16	77,224,656	rs3743759	1.415	0.0002	0.168	-0.323	-0.004	0.59
19	4,471,839	rs10415051	1.400	0.0002	-0.267	0.323	0.010	0.58
2	212,320,111	**rs13386439**	1.365	0.0003	-0.202	0.321	0.001	0.58
4	159,588,976	rs11544037	1.363	0.0006	-0.284	0.303	0.012	0.58
1	173,683,954	rs10798295	1.339	0.0002	-0.197	0.316	0.002	0.57
3	195,839,165	rs68024867	1.337	0.0003	0.280	-0.317	0.004	0.57
8	105,698,914	**rs6985113**	1.308	0.0001	0.259	-0.317	-0.002	0.57
19	4,471,797	rs10421126	1.298	0.0002	-0.274	0.317	0.008	0.56
4	159,939,342	rs591191	1.281	0.0003	0.210	-0.314	0.000	0.56
17	66,244,260	rs3815321	1.194	0.0001	0.131	-0.314	-0.012	0.54
19	10,750,738	**rs1560707**	1.192	0.0003	0.240	-0.295	0.000	0.54
1	94,057,720	rs236326	1.132	0.0012	-0.285	0.284	0.003	0.53
3	73,020,288	rs62249929	1.124	0.0008	-0.221	0.285	-0.001	0.53
15	84,582,124	rs4842838	1.072	0.0007	-0.263	0.291	0.009	0.52
10	74,058,511	rs10762508	1.064	0.0014	-0.279	0.277	0.010	0.52
14	67,117,500	rs6573693	1.027	0.0011	0.226	-0.287	-0.005	0.51
18	54,318,503	rs12956085	1.005	0.0019	-0.261	0.269	0.009	0.50

Table S14: IMPUTE2/BIMBAM QTL genotype-binding affinity association. Only results at the $log_{10}\text{BF} > 1$ threshold are shown. SNP rsIDs highlighred in bold font interesect with top SNPTEST findings.

5 Analysis of VDR-BVs

5.1 General setup

For all the analyses, we only kept those VDR-BV which had non-ambiguous ancestral allele information in 1000 genomes; variants having no ancestral information and triallelic variants were not considered further. For all variants and all VDR-BV analyses below, we defined as $\mathrm{a}_{\mathrm{ANC}}$ the ancestral allele specified for the VDR-BV in the 1000g `AA INFO` field; we defined as $\mathrm{a}_{\mathrm{DER}}$ the VDR-BV's 'derived' allele as follows:

- if $\mathrm{a}_{\mathrm{ANC}}$ corresponds to the 1000g `REF`, then $\mathrm{a}_{\mathrm{DER}}$ is the 1000g `ALT`;
- if $\mathrm{a}_{\mathrm{ANC}}$ corresponds to the 1000g `ALT`, then $\mathrm{a}_{\mathrm{DER}}$ is the 1000g `REF`;

All VDR-BVs analyses of affinity change phenotype in the manuscript refer to the temporal event of a transition of the kind

$$\mathrm{a}_{\mathrm{ANC}} \longrightarrow \mathrm{a}_{\mathrm{DER}}. \tag{S12}$$

Definition of VDR-sBVs and VDR-rBVs

We carried out enrichment tests of VDR-BVs at increasing levels of stringency. We did this to evaluate whether any enrichment results observed for the full set of VDR-BVs were broadly consistent when restricting the set of VDR-BV hypotheses to subsets of higher confidence predictions. We created two sets of higher confidence VDR-BV predictions:

VDR-sBV or VDR *stringent* Binding Variants: to generate these, we retained all VDR-QTL, and only kept a VDR-ASB provided its AlleleSeq FDR was $\mathrm{FDR} \leq 0.01$;

VDR-rBV or VDR *reproducible* Binding Variants: to generate these, we retained all VDR-BVs which were discovered at the same location in at least 2 of samples and which, in addition, showed *coherent* binding affinity change direction for all LCL samples involved in the replication: for instance, a VDR-BV which replicates in samples 1,2,3 and determines a LOB for samples 1,2, but a GOB for sample 3, is not part of the VDR-rBV set.

All enrichment analyses described below were thus performed on each of these three sets: VDR-BVs, VDR-sBVs and VDR-rBVs.

5.2 Analysis of VDR-BV PWM occupancy

We used PScanChIP [Zambelli et al., 2013] to survey the impact of VDR-BVs on known PWM motif intervals. In this analysis we did not restrict the study PWM intervals to those underlying CP_{o3} VDR peaks (see Section 3.4, Page 23 for this): rather, here we considered enrichment at all 43,332 VDR-BVs, even when these VDR-BVs did not intersect a CP_{o3} peak. To do this, we used the following basic workflow:

1. run PScanChIP on bed file containing VDR-BVs coordinates
2. select enriched PWMs from PScanChIP output and generate Encode PWM representation of these
3. run Funseq2 [Khurana et al., 2013] and perform a motif breaking analysis based on the TFM-Pvalue plugin [Touzet and Varre, 2007] to obtain a list of VDR-BVs that hit and significantly break any of the input PWMs.

The list of PWM which are locally ($p < 0.1$) globally or centrally enriched at VDR-BVs according to PScanChIP is in Table S15.

Name	Jaspar ID	Local p-value	Global p-value	Preferred position	Preferred position p-value
**RXRA::VDR**	MA0074.1	1.3E-38 ↑	1.2E-51 ↑	[7,17]	0.0478
NR2F1	MA0017.1	8.9E-19 ↑	0.0004 ↑	[7,17]	0.0039
NR4A2	MA0160.1	1.2E-17 ↑	0.0009 ↑	[9,19]	2.7E-5
Pax6	MA0069.1	1.6E-15 ↑	1.0E-12 ↑	[-3,7]	9.8E-5
Gata1	MA0035.1	6.9E-12 ↑	0 ↑	[3,13]	0.0117
TAL1::GATA1	MA0140.2	2.0E-10 ↑	1.7E-27 ↑	[-61,-51]	0.0246
Ddit3::Cebpa	MA0019.1	8.8E-6 ↑	2.2E-91 ↑	[1,11]	1.5E-6
Sox2	MA0143.3	1.1E-5 ↑	6.2E-17 ↑	[-16,-6]	0.0007
Tal1::Gata1	MA0140.1	1.9E-5 ↑	0 ↑	[-61,-51]	0.0367
MAFF	MA0495.1	0.0010 ↑	1.9E-28 ↑	[49,59]	0.0007
SOX10	MA0442.1	0.0593 ↑	0 ↑	[-17,-7]	0.0145
CTCF	MA0139.1	4.0E-99 ↑	0 ↓	[-6,4]	2.3E-50
RORA_1	MA0071.1	6.1E-11 ↑	7.5E-75 ↑	[-25,-15]	1.0000
GABPA	MA0062.2	2.7E-57 ↑	0 ↓	[-5,5]	2.0E-28
RXR::RAR_DR5	MA0159.1	1.3E-46 ↑	1.1E-18 ↓	[-2,8]	1.1E-12
Esrrb	MA0141.1	2.2E-42 ↑	2.8E-15 ↓	[6,16]	9.0E-7
ELK4	MA0076.1	3.3E-37 ↑	0 ↓	[-6,4]	1.0E-53
NFYA	MA0060.1	9.1E-35 ↑	3.1E-16 ↓	[-2,8]	7.1E-6
ESR1	MA0112.2	1.3E-57 ↑	0 ↓	[-5,5]	2.2E-23
Zfp423	MA0116.1	9.0E-57 ↑	0 ↓	[-3,7]	5.7E-51
ELK1	MA0028.1	1.4E-31 ↑	0 ↓	[0,10]	3.8E-15
Pax2	MA0067.1	1.2E-33 ↑	1.1E-61 ↓	[-6,4]	2.8E-32
CREB1	MA0018.1	8.4E-49 ↑	0 ↓	[-4,6]	1.2E-84
ESR2	MA0258.1	1.1E-52 ↑	5.4E-71 ↓	[-1,9]	4.0E-7
ARID3A	MA0151.1	2.0E-77 ↓	0 ↑	[-62,-52]	3.3E-31
Mycn	MA0104.2	8.4E-54 ↑	0 ↓	[-5,5]	1.1E-161
NFE2L1::MafG	MA0089.1	2.0E-10 ↑	3.5E-27 ↑	[3,13]	1.0000

Table S15: PScanChIP analysis of PWMs enriched at or around VDR-BVs. ↑ indicates over-representation. ↓ indicates under-representation. The columns "Preferred position" indicates the position within the regions where the motif tends to be found more frequently with best matches to the matrix. Coordinates are relative to the center of the regions, which has coordinate 0.

PScanChIP Score > 0.5				PScanChIP Score > 0.7			
PWM	Jaspar ID	#VDR-BVs	%Total	PWM	Jaspar ID	#VDR-BVs	%Total
**RXRA::VDR**	MA0074.1	4,447	**0.1026**	**RXRA::VDR**	MA0074.1	2,867	**0.0662**
ELK1	MA0028.1	2,083	0.0481	ELK1	MA0028.1	2,164	0.0499
Ddit3::Cebpa	MA0019.1	1,849	0.0427	Ddit3::Cebpa	MA0019.1	1,928	0.0445
Znf423	MA0116.1	1,810	0.0418	Znf423	MA0116.1	1,808	0.0417
NFYA	MA0060.1	1,725	0.0398	Gata1	MA0035.1	1,750	0.0404
Gata1	MA0035.1	1,690	0.0390	Pax2	MA0067.1	1,733	0.0400
Pax2	MA0067.1	1,678	0.0387	NFYA	MA0060.1	1,706	0.0394
Tal1::Gata1	MA0140.1	1,586	0.0366	CREB1	MA0018.1	1,612	0.0372
CREB1	MA0018.1	1,549	0.0357	Tal1::Gata1	MA0140.1	1,581	0.0365
MAFF	MA0495.1	1,522	0.0351	RXR::RAR_DR5	MA0159.1	1,544	0.0356
RXR::RAR_DR5	MA0159.1	1,511	0.0349	NR2F1	MA0017.1	1,513	0.0349
Mycn	MA0104.2	1,483	0.0342	SOX10	MA0442.1	1,486	0.0343
SOX10	MA0442.1	1,442	0.0333	MAFF	MA0495.1	1,452	0.0335
ESR2	MA0258.1	1,437	0.0332	Mycn	MA0104.2	1,445	0.0333
NR2F1	MA0017.1	1,425	0.0329	ESR2	MA0258.1	1,428	0.0330
CTCF	MA0139.1	1,300	0.0300	NR4A2	MA0160.1	1,295	0.0299
NR4A2	MA0160.1	1,218	0.0281	NFE2L1::MafG	MA0089.1	1,169	0.0270
Pax6	MA0069.1	1,135	0.0262	Pax6	MA0069.1	1,067	0.0246
NFE2L1::MafG	MA0089.1	1,113	0.0257	CTCF	MA0139.1	1,017	0.0235
TAL1::GATA1	MA0140.2	1,078	0.0249	Gabpa	MA0062.2	984	0.0227
Gabpa	MA0062.2	1,011	0.0233	TAL1::GATA1	MA0140.2	983	0.0227
Sox2	MA0143.3	897	0.0207	Sox2	MA0143.3	929	0.0214
Esrrb	MA0141.1	784	0.0181	Esrrb	MA0141.1	797	0.0184
ARID3A	MA0151.1	712	0.0164	ARID3A	MA0151.1	752	0.0174
ELK4	MA0076.1	665	0.0153	RORA	MA0071.1	656	0.0151
RORA	MA0071.1	633	0.0146	ELK4	MA0076.1	627	0.0145
ESR1	MA0112.2	483	0.0111	ESR1	MA0112.2	162	0.0037
Total in PWMs above	-	38,266	0.8831	-	-	36,455	0.8413
Total	-	43,332	1.0000	-	-	43,332	1.0000

Table S16: Ranking of PWM models hit by VDR-BVs.

Ranking of PWM models hit by VDR-BVs

Having determined a list of affected PWM models and, for such PWM models, a list of intervals where our VDR-BVs were found to significantly disrupt the motif, we assigned VDR-BVs to PWM intervals as follows:

1. If the VDR-BV significantly breaks an RXR::VDR motif (Jaspar code MA0074.1) assign the VDR-BV to RXR::VDR.
2. For all remaining VDR-BVs, assign each to the PWM interval with the PWM model associated with the highest PscanChIP score.

Ranking results are shown, for two different minimum PScanChIP score thresholds ($s > 0.5$ and $s > 0.7$) in Table S16.

Table S16 shows that only 6.6% – 10.5% (2,867 or 4,447) of VDR-BVs lie within (or 'hit') RXR::VDR consensus motifs (Pscanchip score > 0.7 or > 0.5, respectively). Two possible reasons to explain these low proportions are that variants (for example, VDR-BVs) could alter DNA-binding affinity for a) other subunits of a larger multi-molecular complex involving the VDR receptor ('collaborative binding' hypothesis [Heinz et al., 2013]) or for b) factors that inhibit RXR::VDR binding. In the second scenario, indirect RXR-VDR affinity to a site could be increased because of a reduction in affinity of a direct binding factor: if a DNA variant reduces the affinity of a direct binding factor, and this factor is a cofactor of RXR-VDR that inhibits its binding to DNA (e.g. by steric hindrance), then this variant would (indirectly) increase the affinity of RXR-VDR to this site.

5.3 VDR-BV Enrichment Analyses

We tested for enrichment of VDR-BVs in a number of genomic features and disease intervals. We performed tests to verify whether VDR-BVs were overlapping, more often than expected by chance, with any of the following annotation tracks:

1. RXR::VDR DR3 (Jaspar) motif intervals detected under CP_{o_3} binding regions, including class I and class II intervals;
2. ENCODE chromatin segmentation data for LCL sample GM12878[29];
3. ENCODE TFBS clusters (V3) uniformly processed by the ENCODE Analysis Working Group[30];
4. ENCODE DNase clusters (V2) from UW and Duke ENCODE data uniformly processed by the ENCODE Analysis Working Group[31];
5. High-Occupancy Target (HOT) regions [Yip et al., 2012] (track extracted from Funseq annotation);
6. LCL DNase-QTL data (Degner et al. [2012], from the Pritchard QTL resourcee[32]);
7. LCL eQTL data from the Pritchard eQTL resource[33];
8. LCL CEU/YRI eQTL data from the GEUVADIS resource [Lappalainen et al., 2013];
9. CEU LD blocks for genome-wide significant GWAS SNPs from the GRASP catalog, V. 2 Eicher et al. [2015].

We performed three groups of enrichment tests. All tests were based on bootstrapping, however tests differed depending on the features of the annotation we were testing against. Additionally, each group of tests required the definition of slightly different null hypotheses and relative test backgrounds.

Test 1: VDR-BV enrichment at RXR::VDR DR3 motifs in CP_{o_3} binding peaks

In the first group of tests (relative to track (1) above) were wished to assess whether VDR-BV were over-represented in the canonical RXR::VDR DR3 motifs we had detected within CP_{o_3} binding regions and, if so, whether any enrichment at well conserved (class I) and poorly conserved-to-absent (class II) sites differed. Here,

> $\mathcal{H}_0$: VDR-BVs do not impact RXR::VDR motifs within CP_{o_3} binding regions more than any other 1000 Genomes variants in VDR binding regions tested for binding variation potential would,

at the $\alpha = 0.1$ significance level. For this, we created a background composed of 114,155 non-coding (Gencode v. 16 annotation, from Funseq2) 1000 Genomes variants tested by AlleleSeq [Rozowsky et al., 2011] for allelic imbalance (and therefore subsuming VDR ChIP-exo pile-ups of at least 5 reads, see Section 4.1, Pag. 26) in any of the LCL samples, and belonging to one of the following two classes:

- variant is under VDR ChIP-exo read pileup and is labelled with an AlleleSeq `ASYM` outcome (i.e., the variant is a VDR-BV: it significantly impacts VDR binding affinity, $FDR \leq 0.1$);
- variant is under VDR ChIP-exo read pileup and is labelled with an AlleleSeq `SYM` outcome (i.e., the variant does not significantly impact VDR binding affinity, $FDR > 0.1$)

Therefore, the final background is represented by the set of 1000 Genomes variants under VDR ChIP-exo pileups which were tested by AlleleSeq in the LCL samples and therefore had the potential of being VDR binding modifiers.

[29] `hgdownload.cse.ucsc.edu/goldenPath/hg19/encodeDCC/wgEncodeBroadHmm/`
[30] `hgdownload.cse.ucsc.edu/goldenPath/hg19/encodeDCC/wgEncodeRegTfbsClustered/`
[31] `hgdownload.cse.ucsc.edu/goldenPath/hg19/encodeDCC/wgEncodeRegDnaseClustered/`
[32] `eqtl.uchicago.edu/cgi-bin/gbrowse/eqtl/`
[33] `eqtl.uchicago.edu/cgi-bin/gbrowse/eqtl/`

Test 2: VDR-BV enrichment at ENCODE and HOT genomic intervals

In the second group of tests (relative to tracks (2) to (5) above) we were interested in assessing any enrichment of VDR-BVs in chromatin features *over and above* the enrichment of VDR CP_{o_3} binding regions in ENCODE features (promoters, enhancers, DNase I cut sites) and HOT regions we had already observed and documented in the manuscript. Here,

> $\mathcal{H}_0$: VDR-BVs do not impact any specific genomic feature over and above the observed enrichment of VDR CP_{o_3} binding regions in such regions

at the significance level $\alpha = 0.1$. For this, we selected as a background a subset of the background in Test 1, which satisfied *both* the following two conditions:

- The variant is a 1000 Genomes variant which has been tested for allelic imbalance by AlleleSeq in any of the LCL samples (same as test 1);
- The variant is located in any of the CP_{o_3} VDR binding regions.

This yielded a total of 20,330 variants (1000 Genomes phase I V. 3, 2010/11/23). VDR-BVs were considered enriched in a particular annotation if the corrected $p \leq 0.1$.

For both test 1 and test 2, we corrected for genome-wide patterns of GC content variability using isochore tracks, and carried out the bootstrapping procedure using 10,000 randomisations over the selected background using the GAT Heger et al. [2013], similarly to what we did in Section 3.3, Pag. 22.

Test 3: VDR-BV enrichment in QTL and GWAS disease variants

In the final group of enrichment analyses (relative to tracks (6) to (9) above), we were interested in assessing the existence of significant over-representation of VDR-BVs in functional and disease *variants* (unlike with tests 1 and 2, where the enrichment was against *intervals*). Here,

> $\mathcal{H}_0$: VDR-BVs are not dsQTL/eQTL in LCLs, or are not in strong linkage disequilibrium (LD, see next section for our working definition of LD) with genome-wide significant GWAS disease variants more frequently than 1000 Genome variants present in VDR binding sites that were tested for VDR binding affinity variation potential

at the significance level $\alpha = 0.1$. In other words, we test whether our VDR-BVs are more likely to be GWAS tag SNPs (or in strong LD with them) than a stringent background of those 1000 genomes variants which we had tested for VDR binding affinity variation potential (i.e. we restrict the background to only 1000 genomes variants in VDR ChIP-exo peaks, which removes the bias that would have ensued if foreground variants in regulatory regions had been tested against a broad background of all 1000 genomes variant, including variants outside regulatory regions).

Crucially, it was not possible to simply use here the background defined for Test 1, because carrying out bootstrapping tests of variants against variants presents some additional challenges (See for example this[34] discussion). We thus wrote in-house code to perform bootstrapping tests which addressed the following potential sources of confounders and spurious enrichment:

MHC variation Any foreground or background variants in the MHC region (chr6:29,540,169-33,215,544) were not considered in any of the enrichment tests;

1000 genomes ancestral allele annotation Any foreground or background variants lacking explicit ancestral allele annotation in the INFO field of the 1000 genomes vcf were not considered for the tests;

LD-dependence We performed *LD-clumping* of the foreground and background variants: only LD-independent (based on a D' definition of LD, see next Section) VDR-BVs and background variants

[34] `www.cureffi.org/2013/01/29/sampling-a-matching-distribution-for-bootstrapping/`

were kept. If multiple VDR-BVs were in the same LD block, we selected the one which produced the largest mean variation Δ_{BA} in VDR binding affinity , defined as

$$\overline{\Delta}_{\text{BA}} = \frac{1}{N}\sum_{i=1}^{N}\Delta^{i}_{\text{BA}} = \frac{1}{N}\sum_{i=1}^{N}|\text{BA}^{i}(\text{allele}_1) - \text{BA}^{i}(\text{allele}_2)| \tag{S13}$$

where $i = 1, \ldots, N$ is the LCL sample in which the VDR-ASB was detected, and $\text{BA}^{i}(\text{allele})$ is the normalised read count at the heterozygous position for each of the two alleles, as detected by AlleleSeq in the i-th LCL sample.

DAF frequency matching To make sure the enrichment analyses would not be skewed by the capability of the asymmetric binding assay to capture rare variation, and by the fact that rare alleles in QTL or GWAS associations tests are more rarely considered to be GWAS-associated SNPs, we accounted for frequency matching of foreground and backgrounds of the bootstrapping procedure.

Starting from the background set of 114,155 variants (described in Test 1), which we corrected for LD-independence and MHC membership as above, we sampled with replacement 10,000 sets of background SNPs (each one containing the same number of SNPs as the VDR-BV foreground being tested) where we made sure each set was DAF-matched with the foreground, using a binning approach (0.05 increments, 20 bins). When opportune, we computed empirical p-values and corrected for multiple hypotheses using the R function `p.adjust`, with `BH` option.

As the frequency matching was done on the derived allele frequency, rather than the alternate allele frequency (or MAF), any variants showing absent or ambiguous (e.g. triallelic) ancestral allele information in the 1000 Genomes vcf were not considered in the bootstrapping procedure.

Further notes on VDR-BV enrichment at GWAS disease variants

We performed enrichment tests of VDR-BVs against LD blocks defined according to a D'-based definition (Section 3.7, Pag. 25). We used the NCBI GRASP catalog, v. 2 [Eicher et al., 2015] to which we added a recent set of non-MHC Multiple Sclerosis GWAS variants [(IMSGC), International Multiple Sclerosis Genetics Consortium, 2013]. For this analysis, we preferred the NCBI GRASP catalog to the NHGRI-EBI GWAS Catalog [Welter et al., 2014] because of the 19% higher initial number of studies in the GRASP catalog (2,082 GWAS studies in v. 2.0.0.0, compared to 1,751 for the NHGRI GWAS catalog [Welter et al., 2014]). We filtered out any eQTL, methylation QRT and Methylation level GRASP data: a SNP was filtered out if its GRASP `PaperPhenotypeDescription` field contained the string `Quantitative trait` or if its GRASP `Phenotype` contained any of strings

```
Gene expression
Methylation level
Methylation QTL
Differential exon level expression
PC
SM
Serum Metabolite
Serum ratio
Transcription termination
lysoPC
```

Then, we mapped genome-wide significant ($p < 5 \times 10^{-8}$) SNPs (all traits or diseases represented by at least 5 total SNPs) to their corresponding strong LD block using in-house scripts. Beyond these basic filtering steps, we performed no further pre-selection of GWAS traits/diseases to test: if at least 1 VDR-BV in the foreground set was in a strong LD block for a trait/disease, that trait/disease was tested. Enrichments were calculated using an in-house bootstrapping routine as detailed in the previous section. We retained enrichment results passing the FDR ≤ 0.1 threshold. Additionally, if a VDR-BV-to-disease enrichment was supported by only 1 VDR-BV, the association was not presented in Figure 6 (main text) or Figure S20. In other words, after thresholding the FDR, only significant disease associations supported by at least 2 VDR-BVs were kept for display.

Track	Annot	Obs	Exp	CI95low	CI95high	σ	fold	l2fold	p-val
**VDR-BVs** $\pm$ 10KB	ChIPseq VITd genes	466	237.12	220	255	10.54	1.96	0.97	0.0001
**VDR-BVs** $\pm$ 1KB	ChIPseq VITd genes	301	225.10	202	249	14.32	1.33	0.42	0.0001
**VDR-rBVs** $\pm$ 10KB	ChIPseq VITd genes	9	4.96	2	9	2.20	1.68	0.75	0.0636
**VDR-rBVs** $\pm$ 1KB	ChIPseq VITd genes	4	2.37	0	5	1.53	1.48	0.57	0.2173

Table S17: VDR-BV enrichment at differentially expressed genes after calcitriol stimulation.

VDR-BV Enrichment at Differentially Expressed genes following Calcitriol Stimulation

To evaluate whether VDR-BVs lie closer than expected to genes that are differentially expressed upon calcitriol stimulation in LCLs, we retrieved expression data from [Ramagopalan et al., 2010] (Supplementary Table 2). We lifted over hg18 annotation to hg18 using GENCODE [Harrow et al., 2012] v.19 coordinates, retaining only lines marked by a 'gene' and 'protein-coding' field.

We then asked whether the VDR-BVs and VDR-rBVs are closer to the differentially expressed genes compared to the background of all protein-coding GENCODE v.19 genes. We extended each VDR-BV nucleotide position upstream and downstream by either 10kb or 1kb. We then ran GAT enrichment analyses [Heger et al., 2013] correcting for background GC-content and mappability. Results are shown in Table S17.

5.4 Analyses of VDR-BV Effect in RXR::VDR Consensus Motif

Concordance of PWM break effect and binding affinity change (LOB/GOB)

The analysis of genotype/phenotype concordance at VDR-BVs which significantly break the RXR::VDR consensus was performed as follows: for each of N total VDR-BVs tagged by a `MOTIFBR:VDR_JASPAR` by Funseq and for each of M total samples where a significant ASB/QTL event had been detected, we derived the score pair

$$(\mathcal{S}_{\text{gt}}^{i}, \mathcal{S}_{\text{ph}}^{j}) \qquad \forall i \in 1, \ldots, N \qquad \forall j \in 1, \ldots, M \tag{S14}$$

where S_{gt} is a *genotype direction* score defined as

$$\mathcal{S}_{\text{gt}} = \frac{s_{\text{gt}}(\text{a}_{\text{ANC}}) + 1}{s_{\text{gt}}(\text{a}_{\text{DER}}) + 1} \tag{S15}$$

and S_{ph} is a *phenotype direction* score defined as

$$\mathcal{S}_{\text{ph}} = \frac{s_{\text{ph}}(\text{a}_{\text{ANC}}) + 1}{s_{\text{ph}}(\text{a}_{\text{DER}}) + 1}. \tag{S16}$$

Here, the generic s_{gt} is obtained from Funseq2 (motif breaking statistics at the default Funseq p-value threshold; TFMPvalue [Touzet and Varre, 2007]; $p < 4 \times 10^{-8}$) and the generic s_{ph} is the read count at VDR-BVs derived from the VDR-QTL and VDR-ASB output.

Based on these assumptions, we classify a RXR::VDR breaking VDR-BV event as 'concordant' if

$$(\mathcal{S}_{\text{gt}} > 1 \wedge \mathcal{S}_{\text{ph}} > 1) \vee (\mathcal{S}_{\text{gt}} < 1 \wedge \mathcal{S}_{\text{ph}} < 1) \tag{S17}$$

and 'discordant' if

$$(\mathcal{S}_{\text{gt}} > 1 \wedge \mathcal{S}_{\text{ph}} < 1) \vee (\mathcal{S}_{\text{gt}} < 1 \wedge \mathcal{S}_{\text{ph}} > 1). \tag{S18}$$

Binding affinity directionality analysis (LOB/GOB)

The analysis of Loss of Binding (LOB) and Gain of Binding (GOB) phenotype magnitude at VDR-BVs in the RXR::VDR motif was performed similarly to the above.

For this group of analyses, however, we considered all those VDR-BVs which had been annotated by Funseq2 as hitting an instance of the RXR::VDR consensus motif (Funseq2 tag: `TFM:VDR_JASPAR`) and not only the subset of these found to 'break' a motif at the $p < 4 \times 10^{-8}$. Separately, we also considered LOB/GOB calculations for 1000 Genomes variants in RXR::VDR motif instances which had *not*

**Chr.**	**Pos.**	**RS ID**	**Alleles**	**Sample(s)**	**HP**	**Ph_dir**	**DAF**(EUR-AFR)	**Funseq_Annotation**
chr2	9,782,901	rs150039439	GA	NA19213(YRI)	5	LOB	NA-0.002	Enh,TFM,TFP
chr4	3,081,642	rs28393280	AG	NA12383(CEU)	12	LOB	0.04-0.2	DHS,Enh,TFM,TFP — HTT(Intron)
chr4	15,756,102	rs115843103	AG	NA19213(YRI)	12	LOB	NA-0.05	DHS,Enh,TFM,TFP — FAM200B(Distal),FBXL5(Distal)
chr5	1,297,711	rs116433983	CT	NA19249(YRI)	2	LOB	NA-0.04	TFM,TFP
chr7	22,680,066	rs4722163	CA	NA11829(CEU)	11	LOB	0.06-0.01	DHS,TFM,TFP
chr8	50,064,771	rs117660559	GA	NA19190(YRI)	14	LOB	NA-0.03	DHS,Enh,TFM,TFP — C8orf22(Distal)
chr8	67,344,978	rs116029466	AG	NA19189(YRI)	6	LOB	NA-0.04	DHS,TFM,TFP — ADHFE1(Intron&Medial)
chr8	68,146,420	rs146542989	CG	NA19247(YRI)	2	LOB	NA-0.01	DHS,TFM,TFP —ARFGEF1(Intron)
chr8	101,878,883	rs112500060	GT	NA19190(YRI)	2	LOB	NA-0.07	TFM,TFP
chr19	40,862,149	rs148144038	GT	NA19191,NA19189(YRI)	11	LOB	NA-0.0041	DHS,TFM,TFP — PLD3(Intron)
chr19	44,269,709	rs76057752	GA	NA12872(CEU),NA19248,NA19249(YRI)	2	LOB	0.03-0.03	Enh,TFM,TFP
chr21	16,594,530	rs148449534	GT	NA19189(YRI)	10	LOB	NA-0.02	DHS,Enh,TFM,TFP — HSPA13(Distal),NRIP1(Distal)
chr21	27,106,656	rs17001334	AG	NA12383(CEU)	6	LOB	0.09-0.04	TFM,TFP — ATP5J(UTR),GABPA(Promoter)
chr2	134,989,327	rs80279279	GC	NA12489(CEU),NA19213(YRI)	5	GOB	0.9974-0.96	DHS,TFM,TFP — MGAT5(Intron)
chr15	70,006,857	rs71404233	GA	NA19213(YRI)	12	GOB	0.93-0.95	Enh,TFM,TFP — NOX5(Distal),TLE3(Distal)

Table S18: VDR-BVs in bottom (LOB) or top (GOB) deciles of CEU or YRI DAF distributions

passed the Alleleseq test for significant allelic imbalance. For both these distinct groups (VDR-BVs and 'non-VDR-BVs') we tagged a sample as 'LOB' when the transition in Equation S12 resulted in a

$$\mathcal{S}_{\text{ph}} > 1 \tag{S19}$$

and 'GOB' when it resulted in a

$$\mathcal{S}_{\text{ph}} < 1. \tag{S20}$$

LOB or GOB events caused by VDR-BVs which resulted in significant, but discordant across sample, phenotypic effects were not considered.

DAF analysis of VDR-BVs in RXR::VDR consensus

The analysis of Derived Allele Frequencies (DAF) for VDR-BVs in the RXR::VDR motif was performed similarly to the above. For each VDR-BV, we obtained alternate allele count information for the African and European 1000 genomes populations. Those VDR-BVs having no alternate allele count information in both cohorts were not considered further. The alternate allele count was mapped using the ancestral allele count information to obtain the derived allele count. DAF data was only considered when relative to any of the two hexamers in the consensus RXR::VDR motif (positions 1-5 and positions 10-15). DAF values for VDR-BVs in the non specific DR3 3nt spacer sequence (positions 7-9) were not considered. All significance analyses were performed using non-parametric tests in R.

6 Supplementary Figures

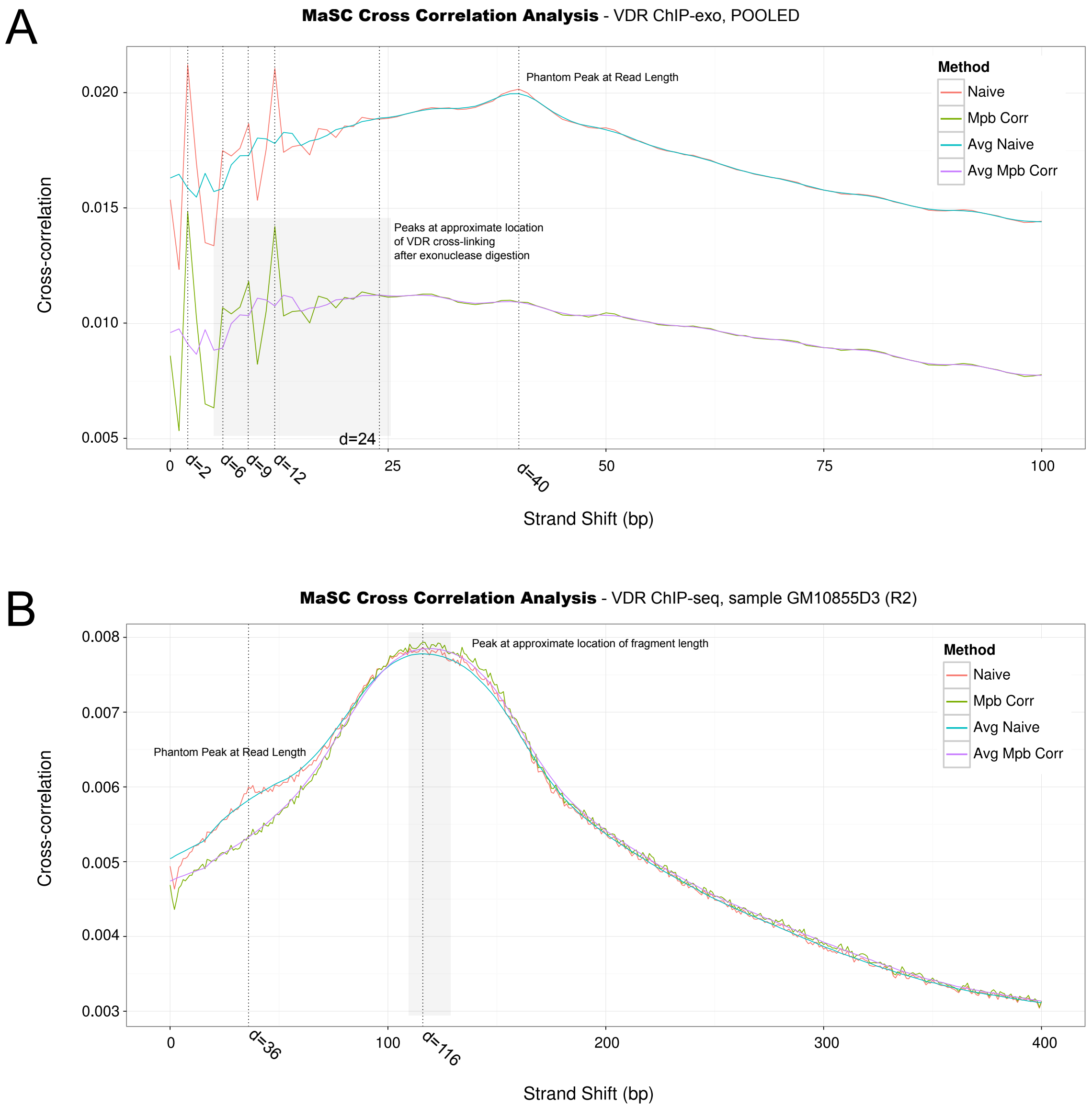

Figure S1: Cross-correlation profiles. **A**: pooled ChIP-exo data. **B**: sample VDR ChIP-seq data from Ramagopalan et al. [2010] (sample GM10855, replicate number 2, calcitriol stimulated). Cross-correlations were calculated for $d = [0, 1500]$ nt. Here, only the intervals $d = [0, 100]$ for **A** and $d = [0, 400]$ for **B** are shown for clarity. The vertical dotted lines mark the position of the phantom peak at read length ($d = 40$nt for the ChIP-exo data and $d = 36$nt for the ChIP-seq data) and the approximate positions for the consensus fragment length $\tilde{d}_{\mathrm{fragment}}$ (for the ChIP-seq data) and $\tilde{d}_{\mathrm{CL}}$ (for the ChIP-exo data). Plots marked with ‘avg’ denote cross-correlation profiles smoothed with 2nt (A) and 15nt (B) sliding windows.

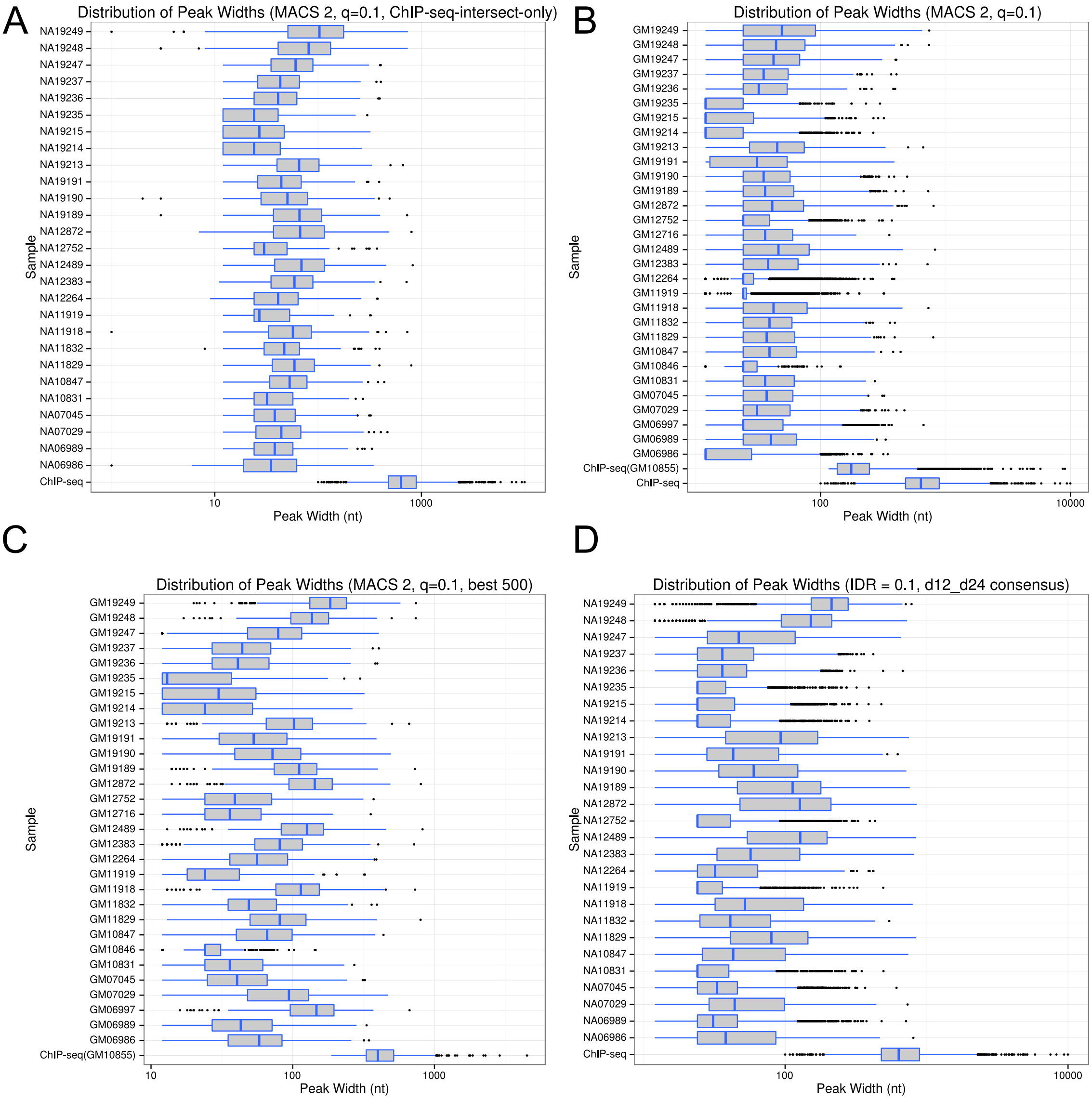

Figure S2: Distribution of Peak Widths for the ChIP-exo samples and comparison with ChIP-seq samples. **A**: peaks obtained with MACS 2 merging two peaksets for each sample, for $\tilde{d}_{\mathrm{CL}} = 12$ and $\tilde{d}_{\mathrm{CL}} = 24$, $q = 0.1$ in both cases. **B**: peaks obtained with MACS 2 merging the best 500 peaks (measure: MACS 2 p-value) from two peaksets for each sample, for $\tilde{d}_{\mathrm{CL}} = 12$ and $\tilde{d}_{\mathrm{CL}} = 24$. **C**: peaks obtained as in **A**, however only peaks intersecting with VDR ChIP-seq consensus peakset from Ramagopalan et al. [2010] are plotted. **D**: peaks obtained via IDR thresholding, at the IDR=0.1 level. For comparison, distributions are plotted also for the VDR ChIP-seq consensus peakset from Ramagopalan et al. [2010] (label: 'ChIP-seq', **A,B,C,D**) and for a MACS2 analysis ran on ChIP-seq sample GM10855 (replicate number 2, calcitriol Activated, $\tilde{d}_{\mathrm{fragment}} = 116$, $q = 0.1$, label: 'ChIP-seq(GM10855)', **A,B,C,D**).

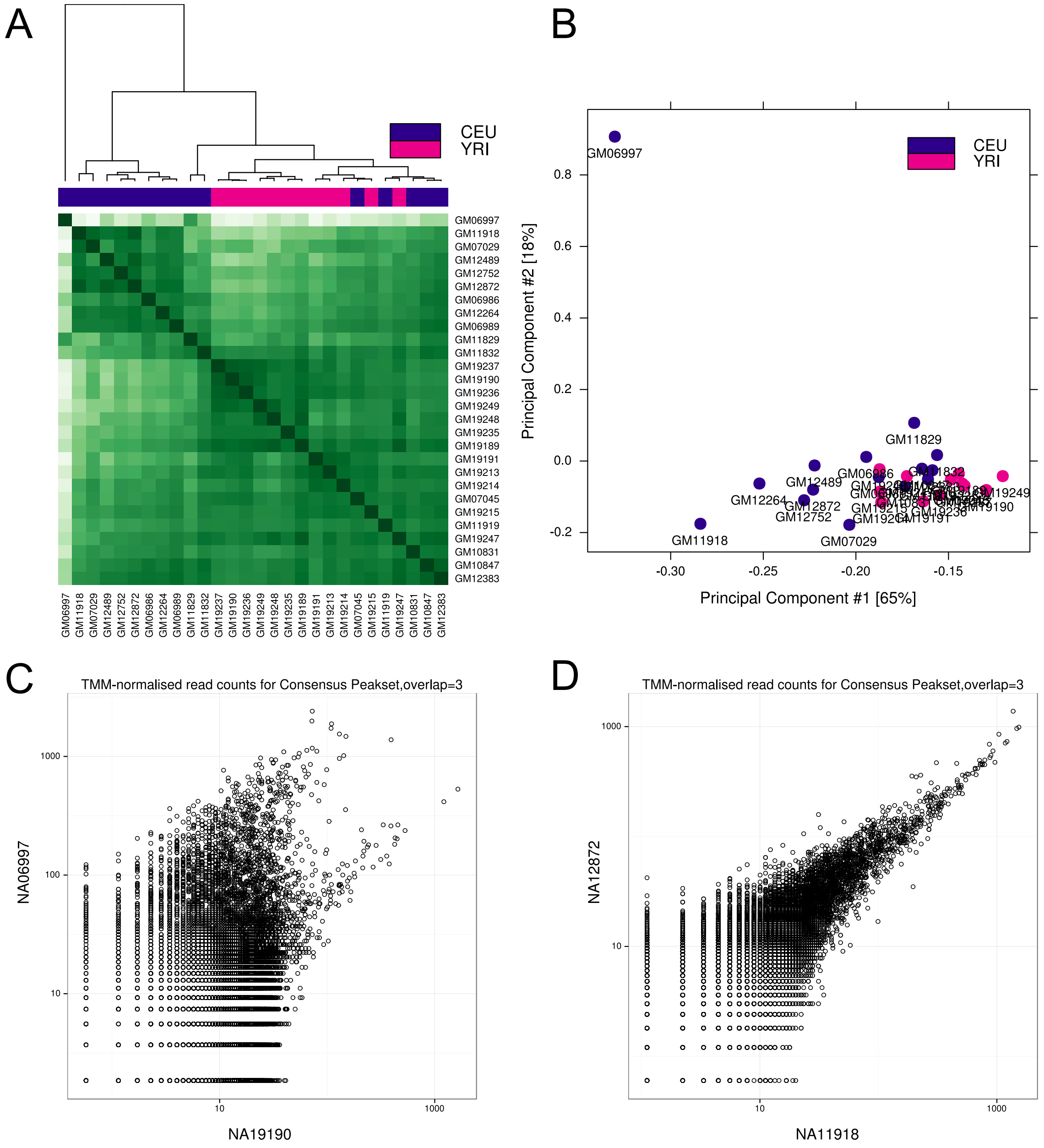

Figure S3: Summary plots for binding affinity data (TMM-normalised read counts) for the consensus VDR binding sites in Table S10, Pag. 19. Data shown is based on a consensus set of 16,563 sites, overlapping 3/28 samples. **A**: sample-level correlation heatmap. **B**: PCA analysis. **C**: Scatterplot showing example of poorly correlated sample pair. **D**: scatterplot showing example of well correlated sample pair.

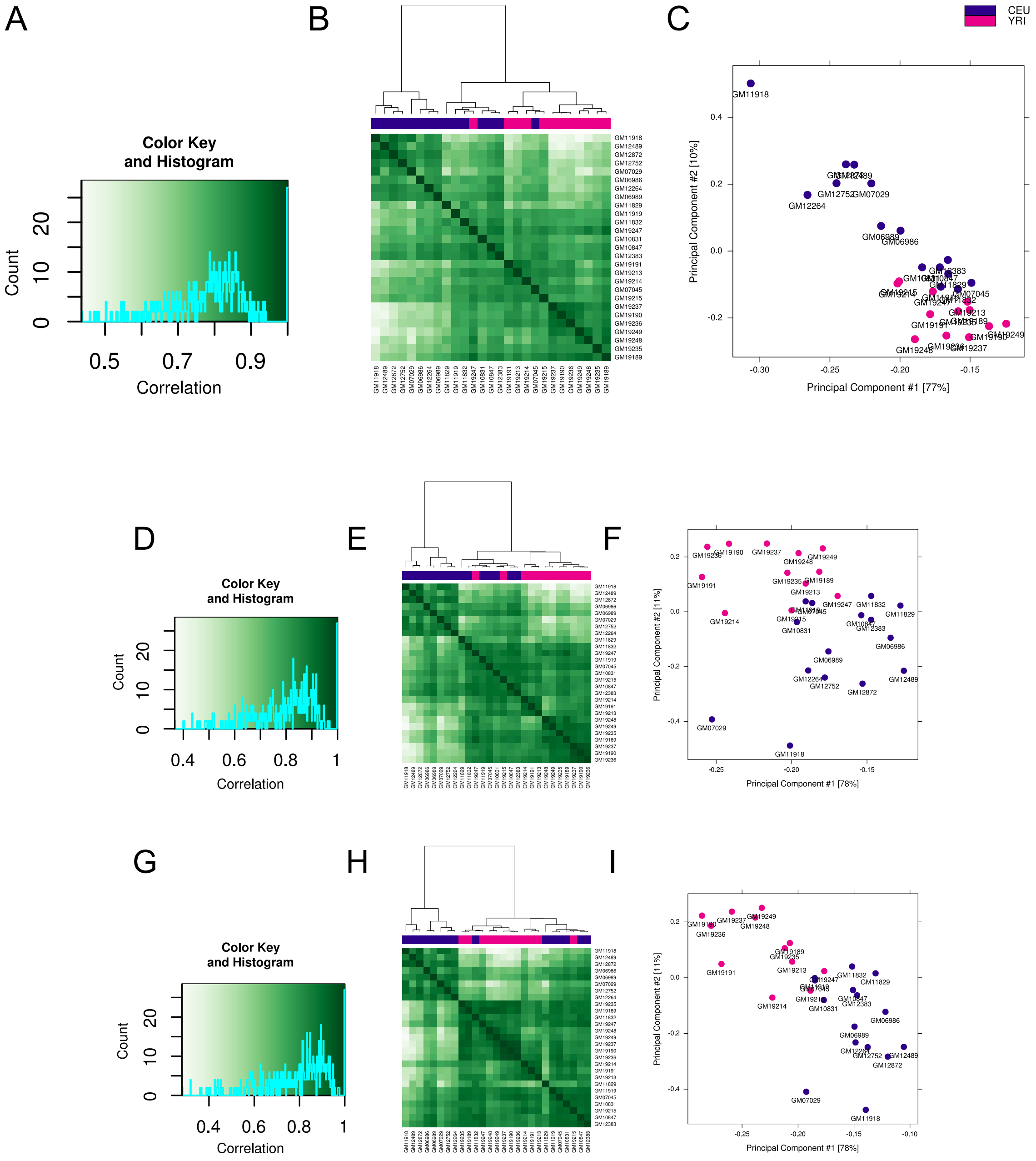

Figure S4: Summary plots for the binding affinity data (TMM-normalised read counts) for the consensus VDR binding sites in Table S10, Pag. 19. **A-C**: final consensus set of 15,509 sites, each called in at least 3/27 samples. **D-F**: consensus set of 2,329 sites, each called in at least 10/27 samples. **G-I**: consensus set of 732 sites, each called in at least 20/27 samples. **A,D,G**: sample-level correlation histogram. **B,E,H**: sample-level correlation heatmap. **C,F,I**: principal component analysis.

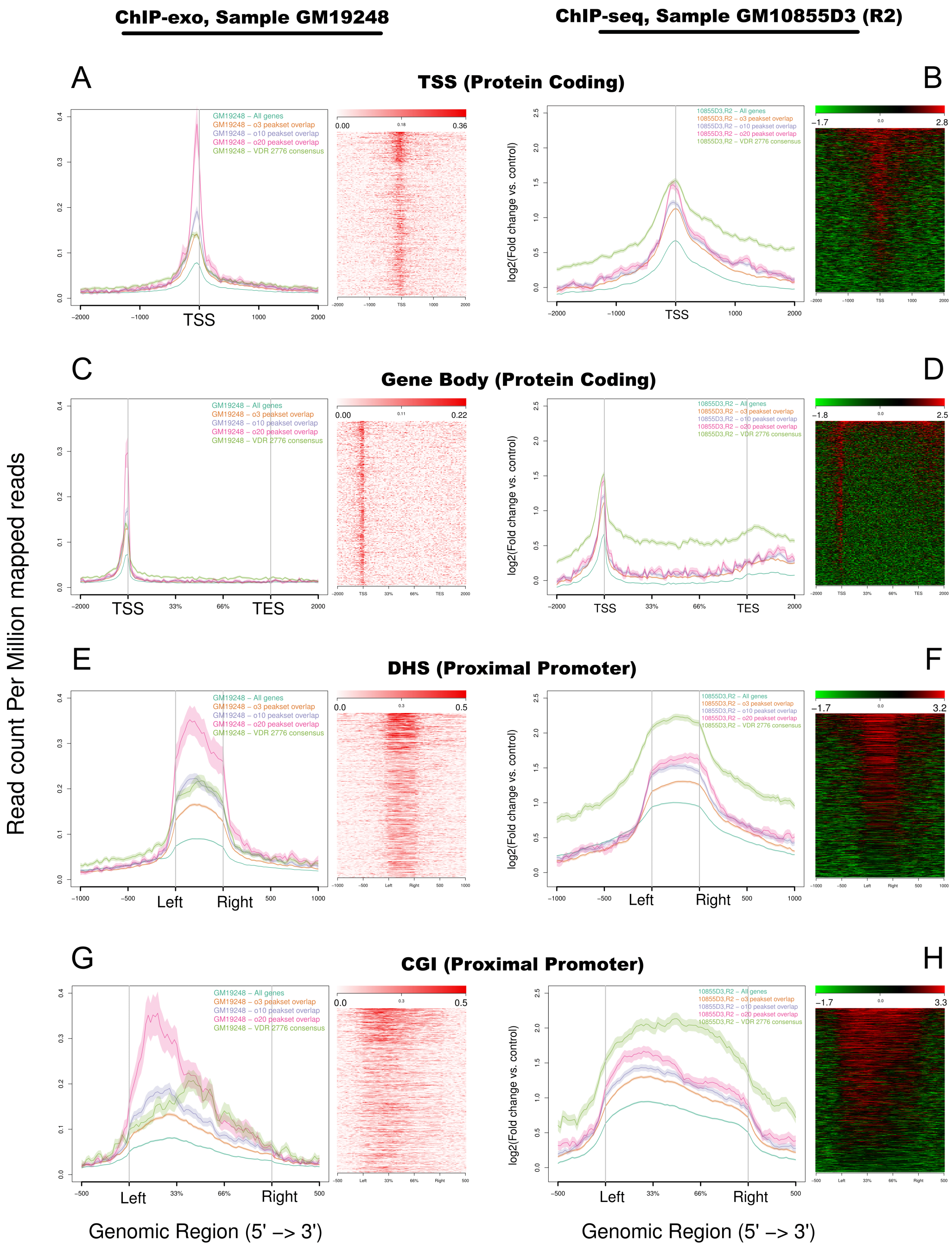

Figure S5: VDR binding signal profile for a ChIP-exo sample (NA19248, *left column*) and a ChIP-seq sample from the data in Ramagopalan et al. [2010] (NA10855D3-R2, *right column*). For each panel, a pile-up meta-profile (Ensembl v. 75) averaged over genes is shown. Several traces (with matching SE) are shown in each panel, corresponding to VDR pile-up at features for all 1) Ensembl 75 protein coding genes, 2) genes in proximity (proximity = ± 1 kb) of CP_{o_3} / $CP_{o_{10}}$ / $CP_{o_{20}}$ VDR intervals, 3) genes in proximity of the VDR ChIP-seq consensus peakset (2,776 peaks) from Ramagopalan et al. [2010]. For the ChIP-seq profiles (*right column*) meta-profiles show fold change of IP versus control signal. The ChIP-exo and the ChIP-seq samples shown are characterised by comparable numbers of mapping reads. Heatmaps are shown only for genomic features associated to the CP_{o_3} (left column) and VDR ChIP-seq (right column) consensus peaksets, respectively. Heatmap width has been rescaled to minimum common width for clarity.

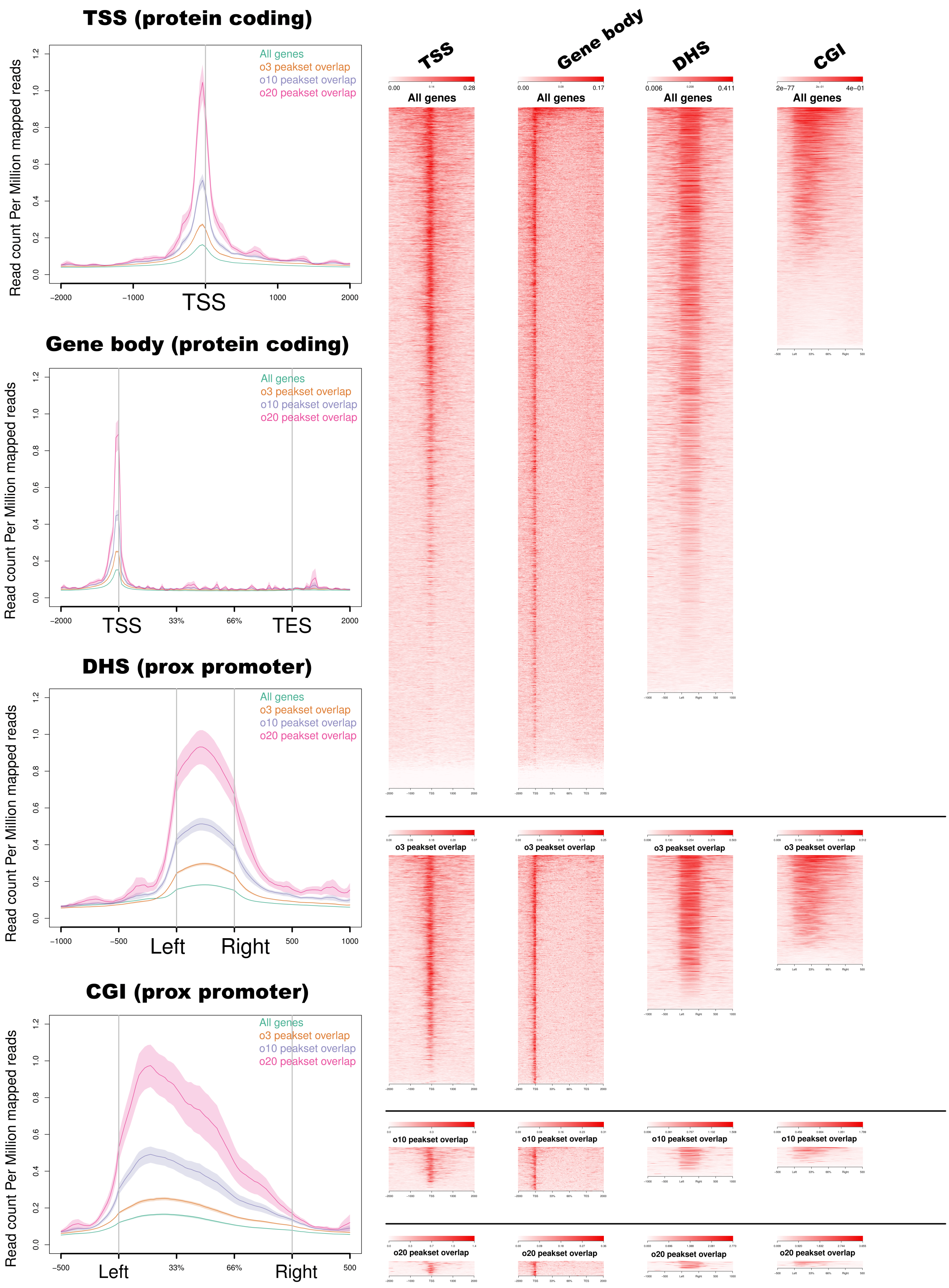

Figure S6: VDR ChIP-exo signal profiles at common genomic features based on the pooled data from the 27 samples.

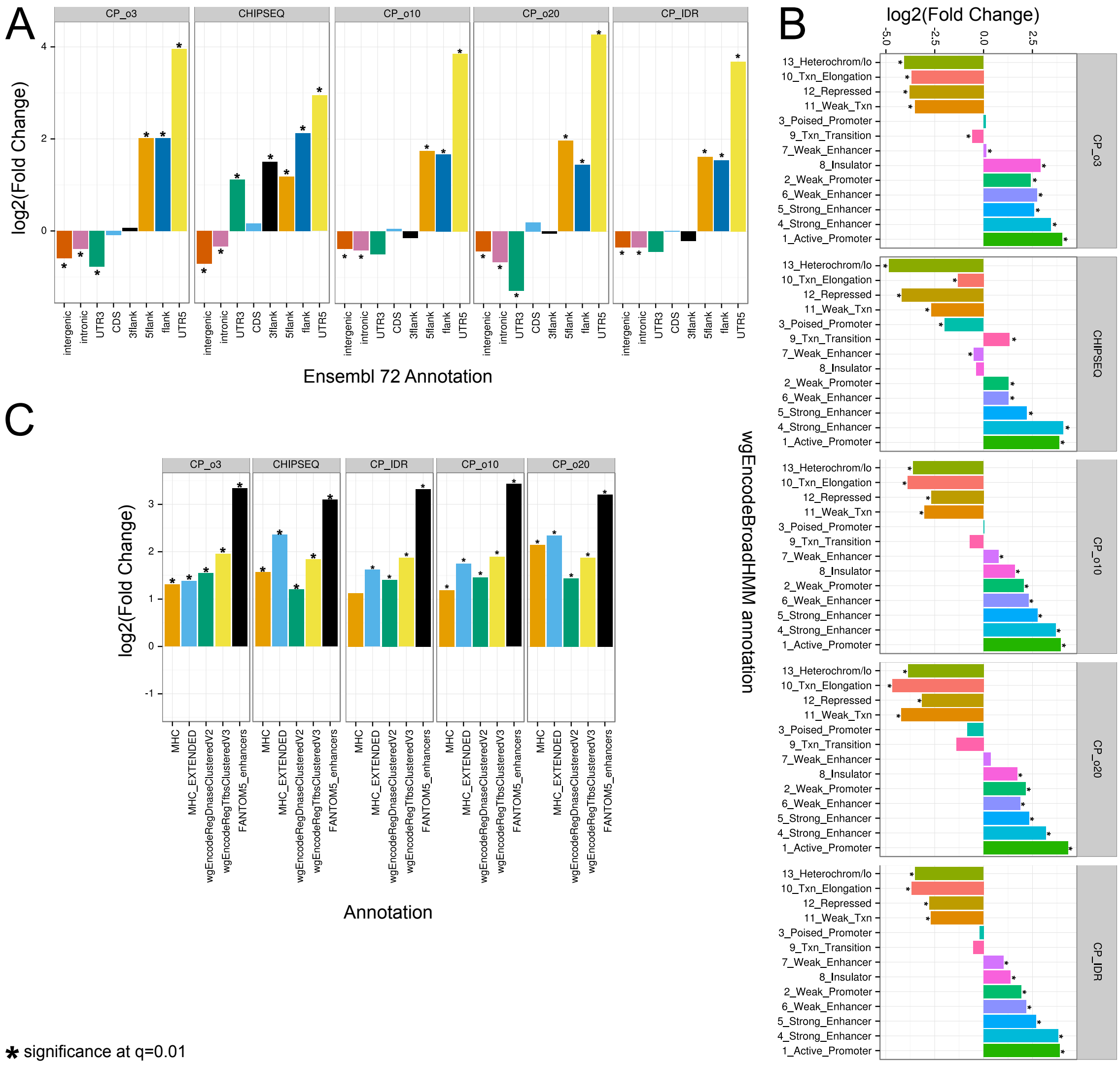

Figure S7: Genomic Association Testing of VDR binding regions at several genomic features. **A**: Genomic Association Testing at gene-centered annotation (Ensembl 72). **B**: Genomic Association Testing of VDR binding regions at chromHMM Broad Chromatin Marks (ENCODE). **C**: Genomic Association Testing at several features: intervals for a definition of the Major Histocompatibility Complex (MHC); intervals at a stringent definition for the Major Histocompatibility Complex (MHC_EXTENDED); clustered DNase sites from ENCODE; clustered Transcription Factor hubs from ENCODE; FANTOM5 enhancers sites. An asterisk indicates significance at the adjust p-value $q = 0.01$ threshold.

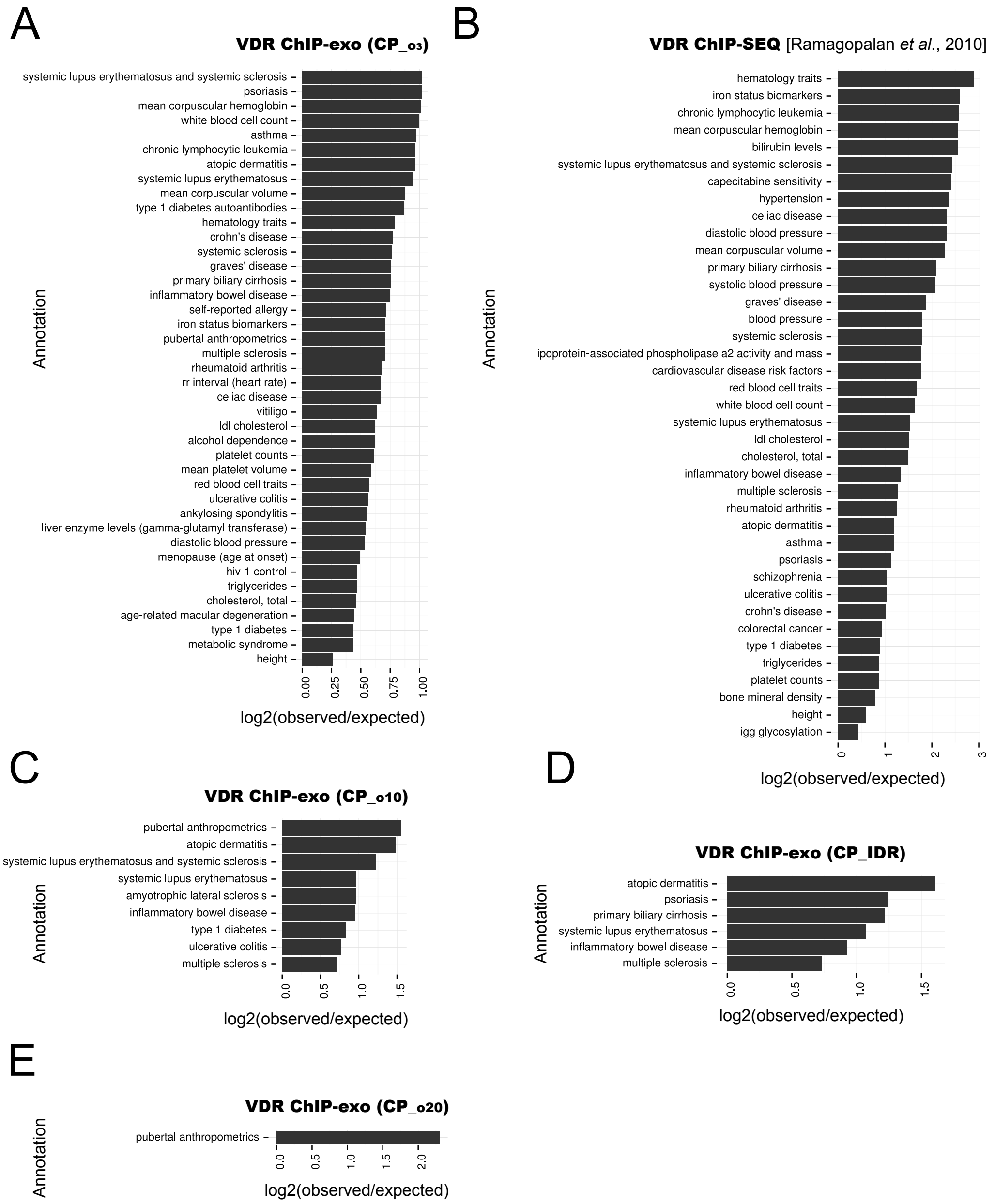

Figure S8: Genomic Association Testing — GWAS catalog intervals. Traits and Diseases showing enrichment of VDR binding (CP_{o_3} peakset) within intervals identified by GWAS. All diseases/traits in the GWAS catalog (accessed 2014/02/18) represented by at least 20 SNPs were analyzed and those showing significant enrichment of VDR binding at 1% FDR threshold are shown. Bars map to fold change of observed vs. expected overlaps. Disease intervals are defined, for backward compatibility, as in [Ramagopalan et al., 2010]. **A**: results for VDR CP_{o_3} ChIP-exo binding regions. **B**: results for VDR CHIP-seq binding regions from Ramagopalan et al. [2010]. **C**: results for VDR $\mathrm{CP}_{o_{10}}$ ChIP-exo binding regions. **D**: results for VDR CP_{IDR} ChIP-exo binding regions. **E**: results for VDR $\mathrm{CP}_{o_{20}}$ ChIP-exo binding regions.

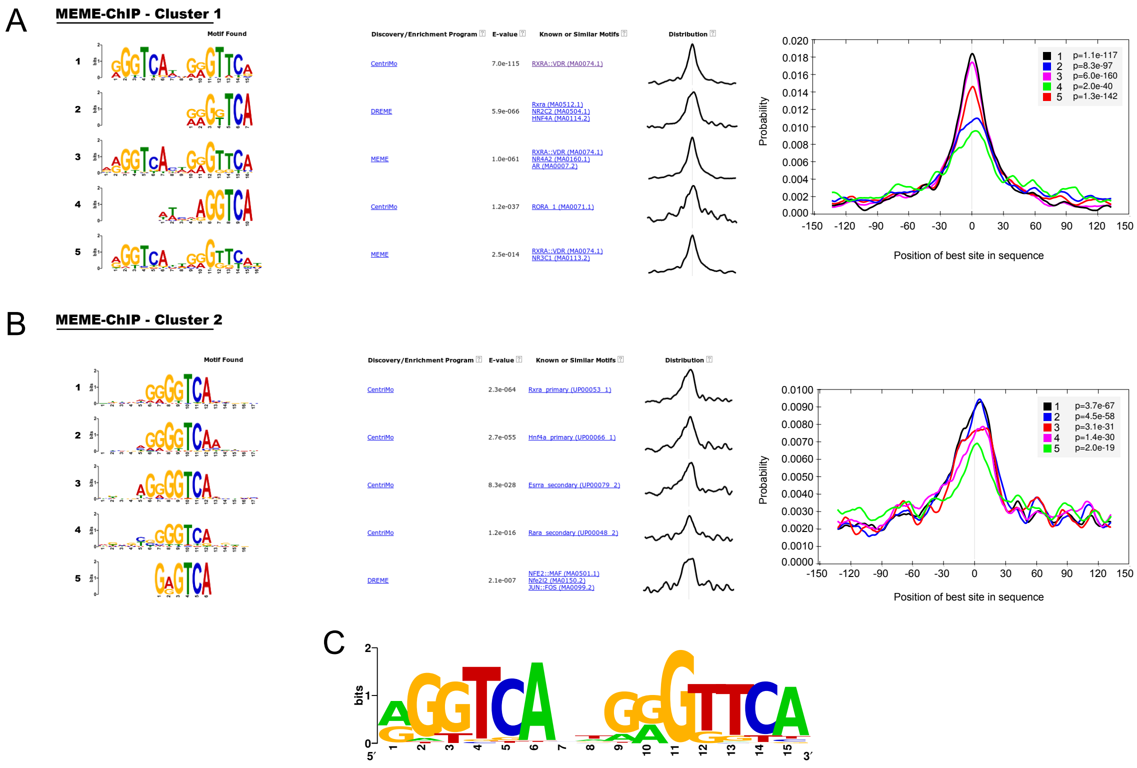

Figure S9: *De novo* motif analyses at genomic regions identified by VDR ChIP-exo peaks. Results refer to VDR ChIP-exo $\mathrm{CP}_{o_{10}}$ peak set (2,329 regions, 15% of the total number of regions in main CP_{o_3} consensus peak set). Panels A and B show the 2 most relevant clusters of motifs, ordered by E-value. For panels A and B, all motifs are centrally enriched, with enrichment pattern shown in last column (distribution). Panel C shows the highest ranking motif found by XXmotif, which closely resembles the canonical RXR::VDR DR3 motif.

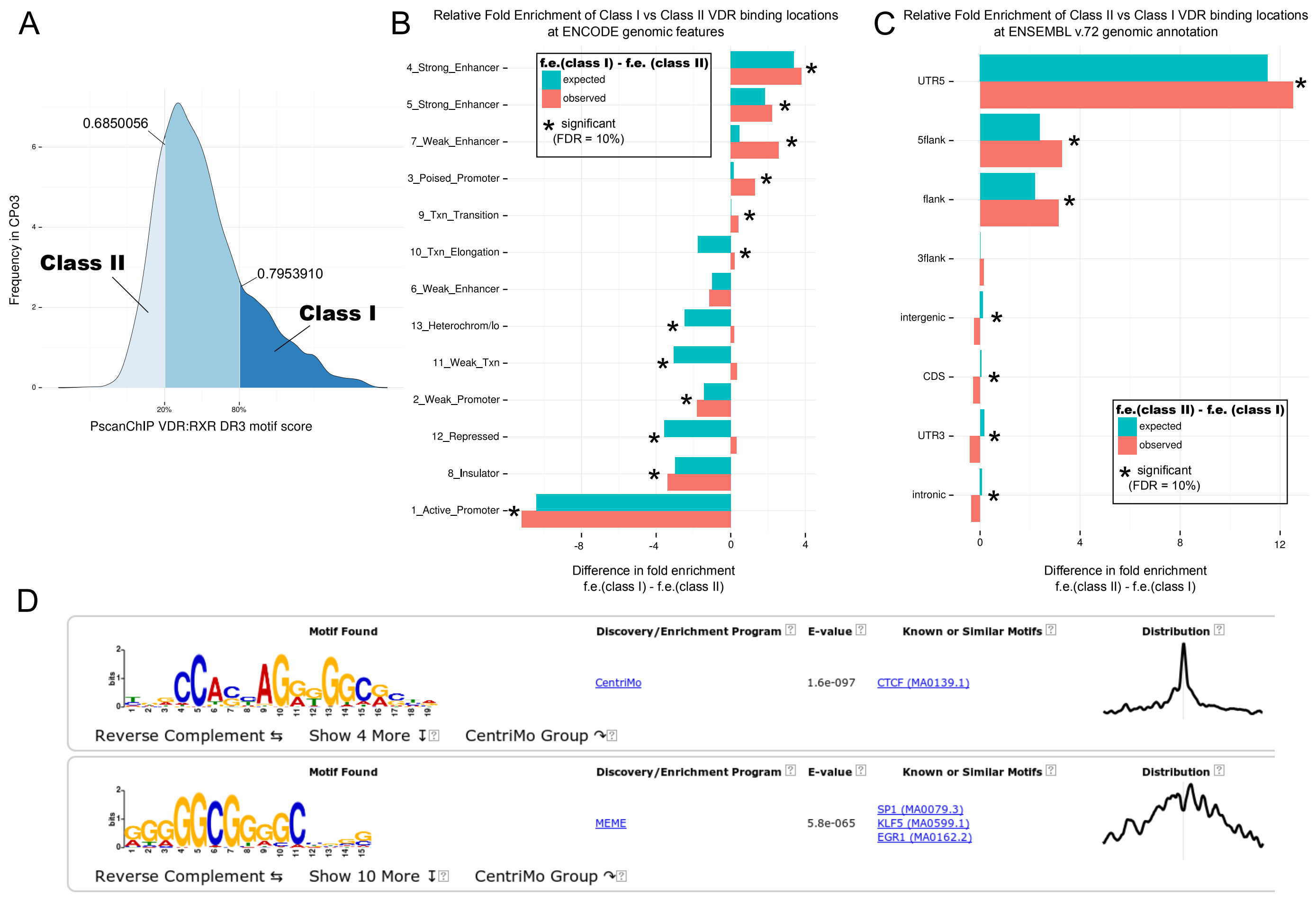

Figure S10: **A**: PScanChIP Motif Score Distribution for the RXR::VDR DR3 consensus motif. Bottom 20% (class II) and Top 20% (class I) quantiles are shown, together with corresponding score cut. **B,C**: differential enrichment of class I vs class II VDR binding sites at ENCODE genomic features (**B**) and Ensembl v. 72 genomic annotation (**C**). Grey bars indicate that the fold change between observed and expected fold enrichment is not significant at the 1% FDR. **D**: *de novo* motif analysis and central enrichment at class II VDR binding sites.

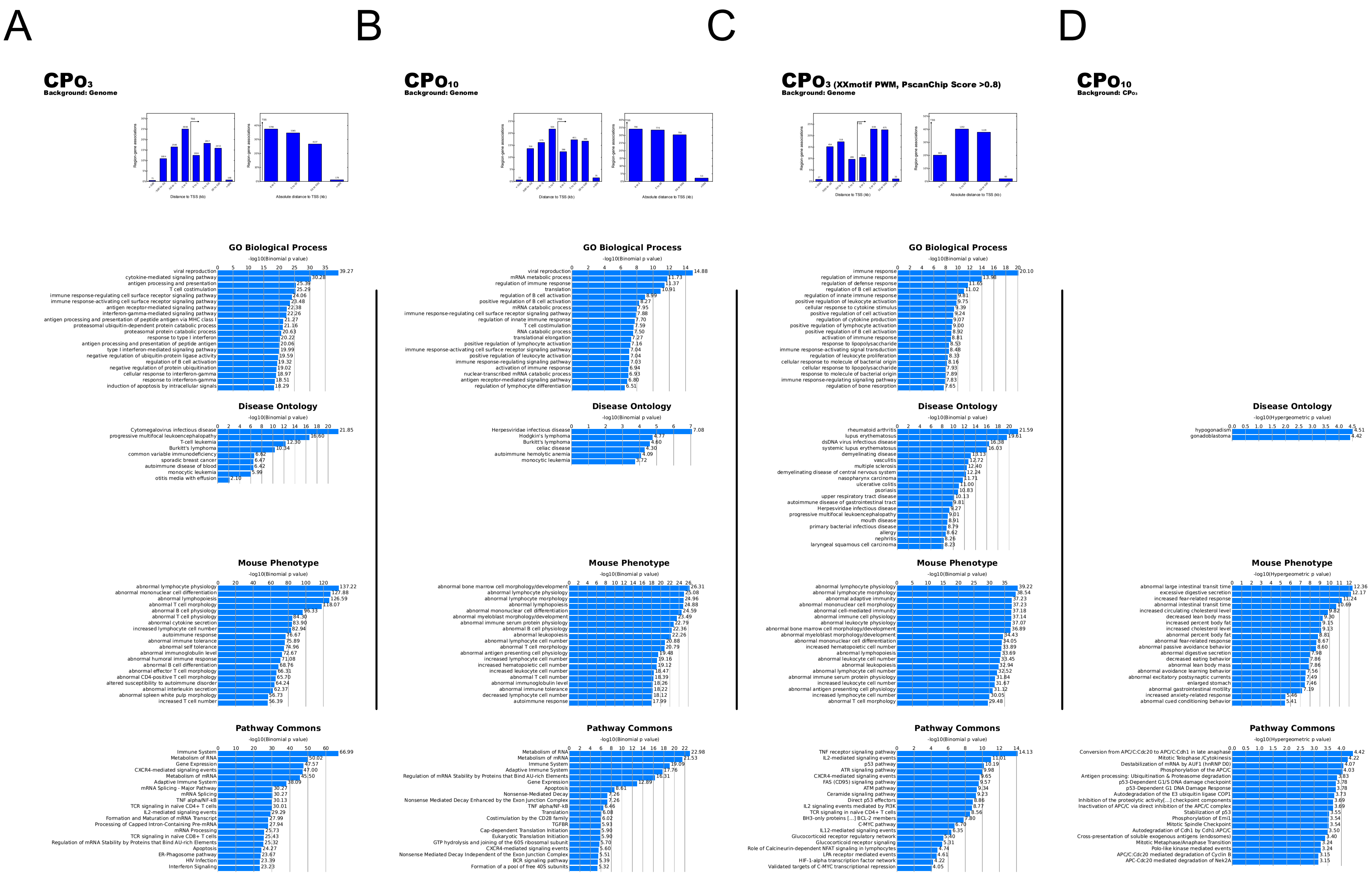

Figure S11: Supplementary GREAT analysis enrichment results. The panel shows a graphical summary of a gene enrichment analysis carried out using GREAT [McLean et al., 2010] to evidence any enrichment of gene annotation in the proximity of VDR ChIP-exo binding sites. For each VDR peak set, we considered the following annotations: GO Biological Process, Disease Ontology, Mouse Phenotype and Pathway Commons. Peaksets as follows: **A**: CP_{o_3}. **B**: $\mathrm{CP}_{o_{10}}$. **C**: A selection of CP_{o_3} VDR binding sites containing strong (PScanChIP Score > 0.8) instances of the consensus RXR::VDR motif. **D**: relative enrichment of $\mathrm{CP}_{o_{10}}$ sites over CP_{o_3} sites. GREAT settings used are detailed in the Supplementary Document.

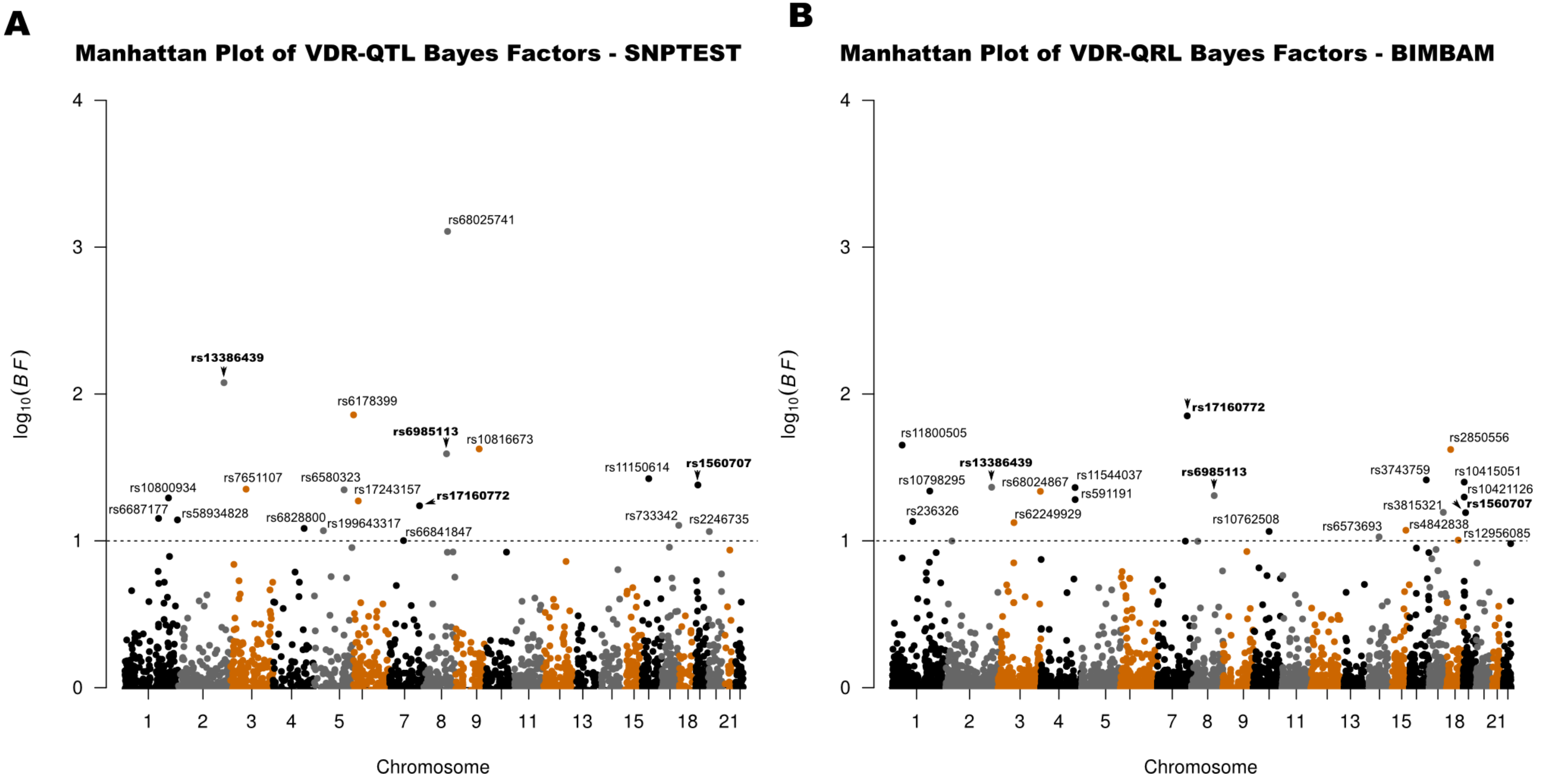

Figure S12: Manhattan Plots for the VDR-QTL Bayesian association analysis. **A**: SNPTEST analysis. **B**: BIMBAM analysis. A suggestive line is set at $\log_1 0(BF) = 1$, i.e. $M_1 = 10 \times M_0$. SNP RsIDs highlighted in boldface are observed in both analyses.

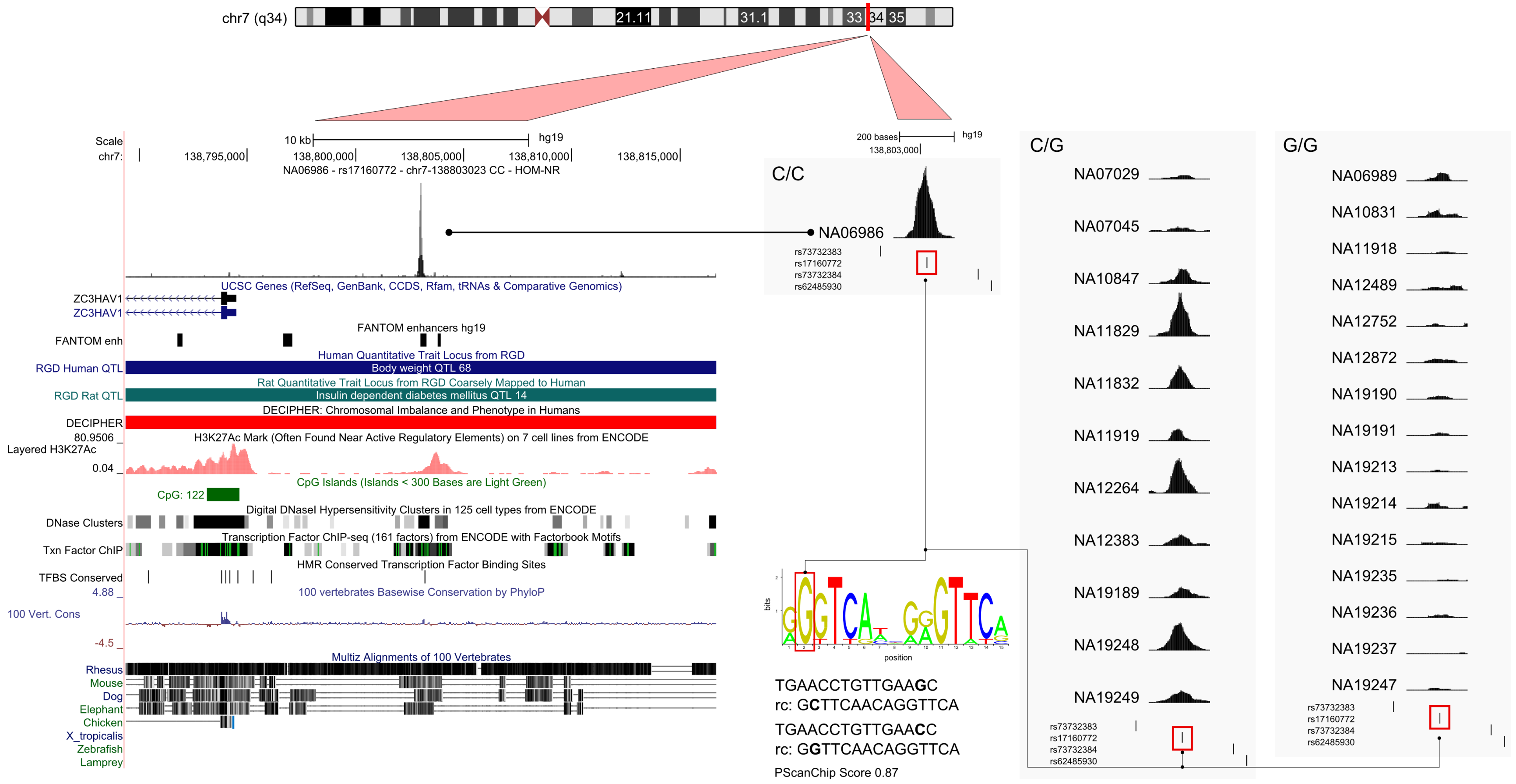

Figure S13: Sample VDR-QTL call at chromosome 7. Summary image for VDR binding region containing rs17160772, a SNP which associates with maximum binding affinity corresponding with the homozygous ancestral allele, intermediate levels of VDR binding for the heterozygous, and low to absent VDR binding affinity for the homozygous derived allele. The variant hits a conserved G at position 2 in the consensus RXR::VDR motif. The binding region is a known FANTOM5 enhancer location.

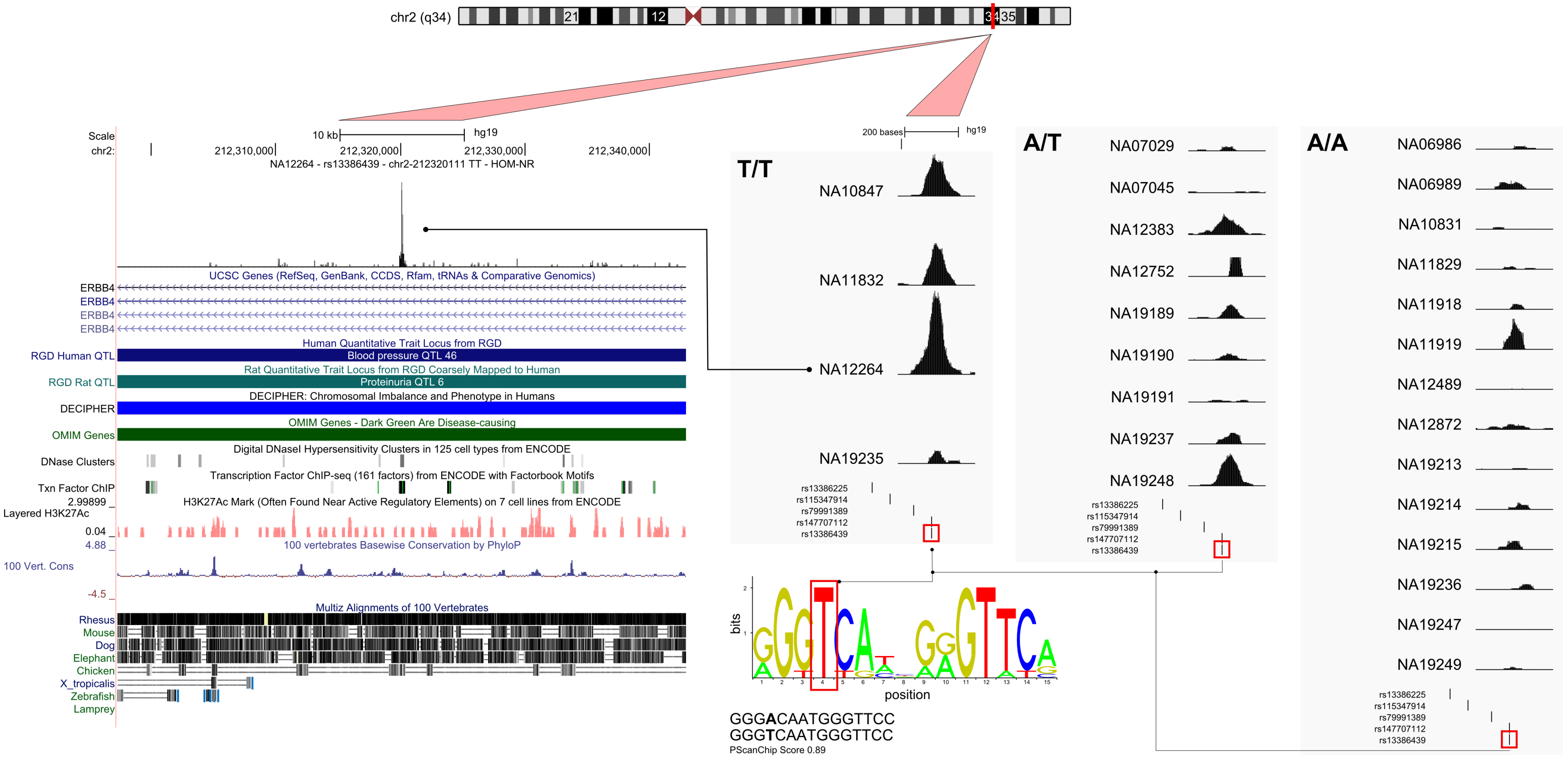

Figure S14: Sample VDR-QTL call at chromosome 2. Summary image for VDR binding region containing rs13386439, a SNP which associates with maximum binding affinity corresponding with the homozygous derived allele (TT), intermediate levels of VDR binding for the heterozygous, and low to absent VDR binding affinity for the homozygous ancestral allele (AA). The variant hits a conserved T at position 4 in the consensus RXR::VDR motif. The binding region is intronic to ERBB4.

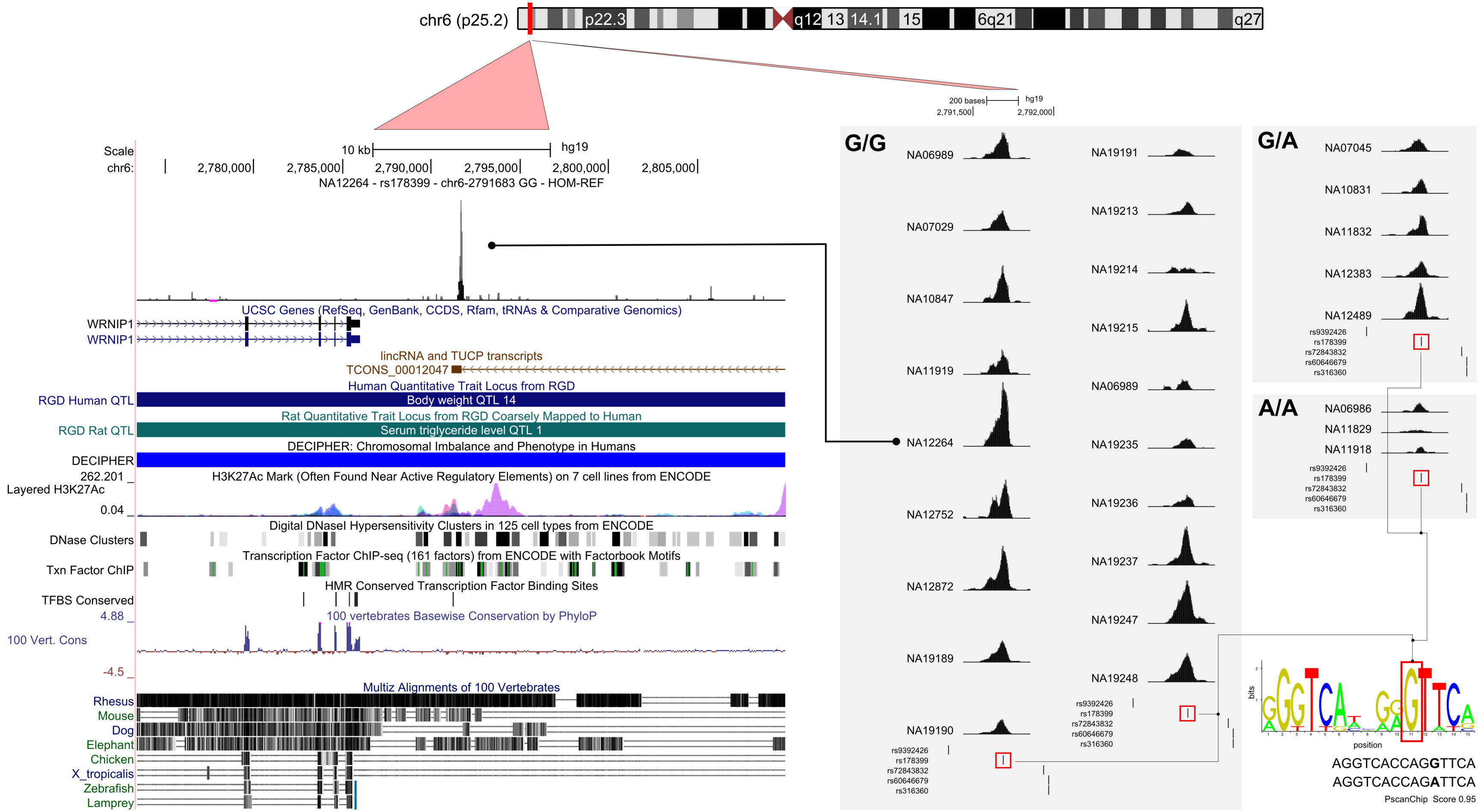

Figure S15: Sample VDR-QTL call at chromosome 6. Summary image for VDR binding region containing rs178399, a SNP which associates with maximum binding affinity corresponding with the homozygous ancestral allele (GG), intermediate levels of VDR binding for the heterozygous, and low to absent VDR binding affinity for the homozygous derived allele (AA). The variant hits a conserved G at position 11 in the consensus RXR::VDR motif.

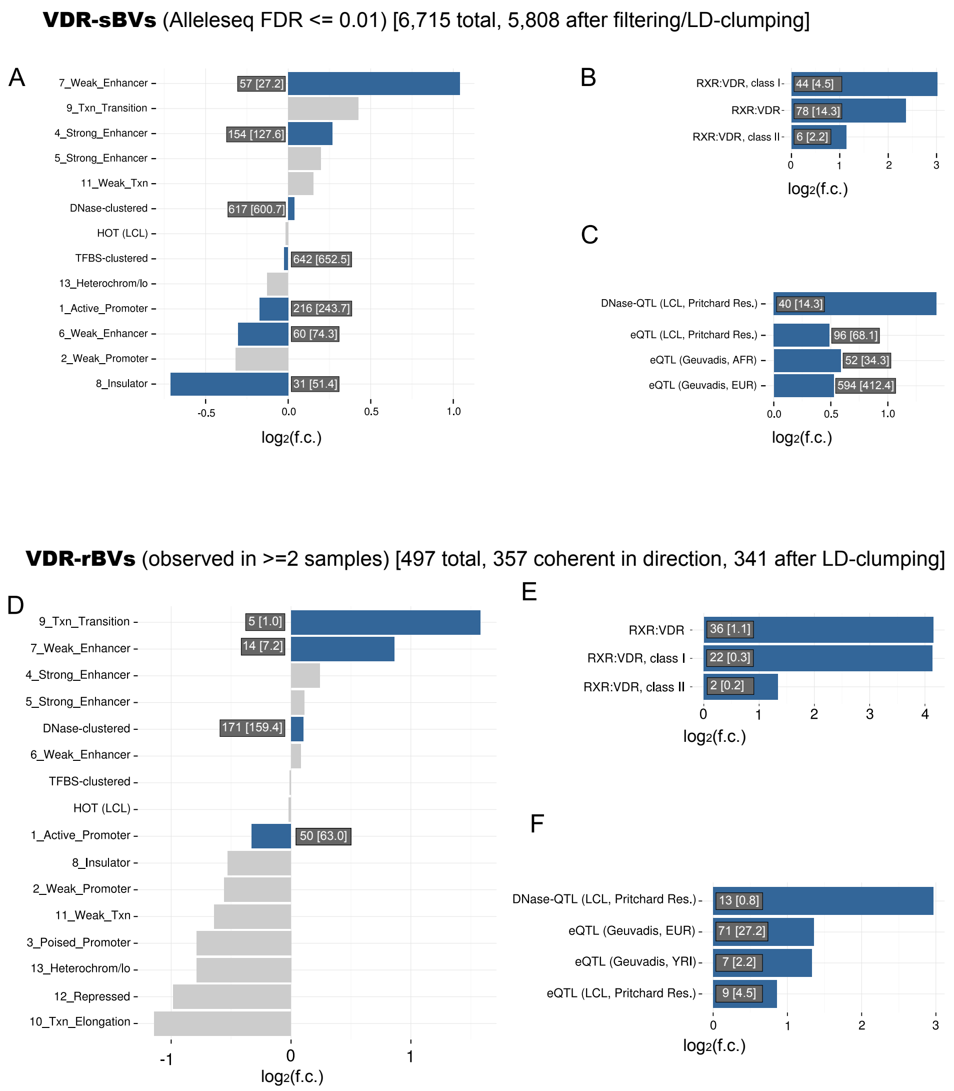

Figure S16: Genomic association testing of VDR-sBVs/VDR-rBVs with functional annotation. **A-C**: results are shown for VDR-sBVs, or *stringent* VDR-BVs (AlleleSeq FDR ≤ 0.01). **D-F**: results are shown for VDR-rBVs or *reproducible* VDR-BVs (i.e. VDR-BVs which are called at the same genomic position in ≥ 2 LCL samples considered). **A** (**D**): Enrichment of VDR-sBVs (VDR-rBVs) within ENCODE chromatin state segmentation tracks for LCL NA12878, TF-dense regions (HOT [Yip et al., 2012] and ENCODE clustered TF binding sites) and DNase hypersensitive areas (ENCODE). **B** (**E**): Enrichment of VDR-sBVs (VDR-rBVs) in RXR::VDR consensus motif intervals present in VDR CP_{o3} binding regions. **C** (**F**): Enrichment of VDR-sBVs (VDR-rBVs) at DNase-QTLs and eQTLs from the Pritchard resource and CEU/YRI LCL eQTLs from the GEUVADIS resource. Significant enrichments are indicated using blue histogram bars (f.c. = fold change of observed versus expected overlaps; $\mathrm{FDR} = q < 0.1$; numbers in a box close to each bar indicate 'observed [expected]' overlap counts; grey bars indicate lack of significance). For panels **A** and **D**, enrichments shown are above-and-beyond the previously observed enrichments of VDR CP_{o3} peaks in the same functional annotation classes (relative to a background of 20,330 1000 Genomes SNPs in CP_{o3} VDR binding regions). For panels **B** and **E**, enrichments are relative to a background of 114,155 1000 Genomes variants lying under VDR ChIP-exo read pileups (≥ 5 reads) which had been tested as potential VDR binding affinity modifiers. This background was further corrected for the analyses in panel **C** and **F**, were only LD-independent foreground VDR-BVs were tested and 10,000 DAF-matched random background sets were extracted with replacement from the main set of 114,155 background variants. In panel **F**, 71 VDR-rBVs (20% of the independent VDR-rBV set) are LCL eQTLs in the GEUVADIS CEU panel, a 2.5-fold enrichment over the null expectation

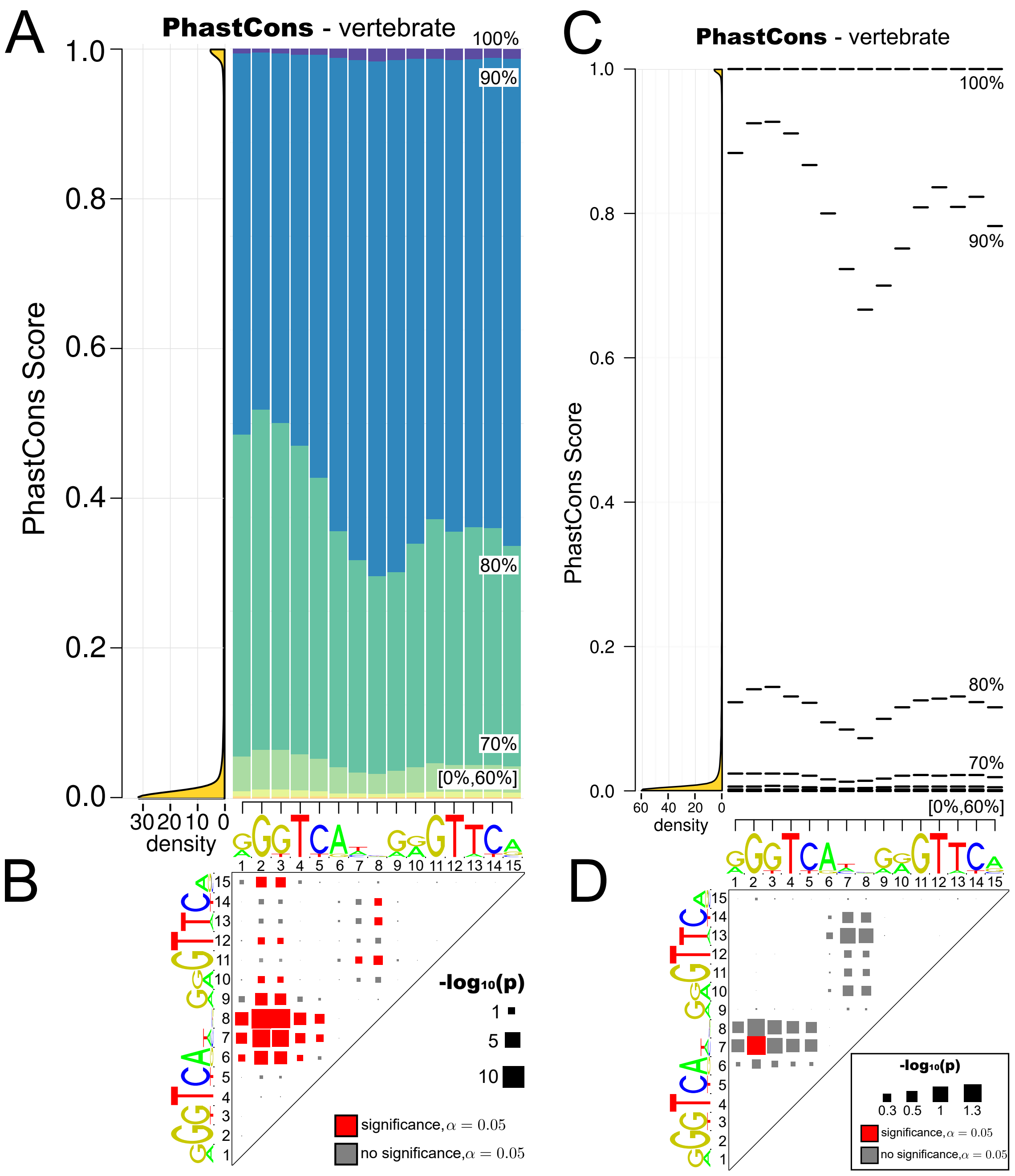

Figure S17: Evolutionary conservation of VDR-BVs at bound RXR::VDR motif intervals. **A**: Distribution of vertebrate Phastcons scores across the RXR::VDR motif for genomic positions in the CP_{o_3} consensus VDR binding regions. Panel **A**, **left**: density plot (yellow) summarising the distribution of PhastCons scores aggregated across all 15 RXR::VDR nucleotide positions. Panel **A**, **right**: deciles of per-nucleotide distributions. **B**: Hinton plot showing results for *post-hoc* Kruskal-Wallis pairwise analyses of nucleotide position-to-conservation dependence, which highlights significant differences (shown as red squares) between nucleotide position pairs within the RXR::VDR motif. **C** and **D**: same as in **A** and **B**, but at RXR::VDR motif intervals bound at class I sites only.

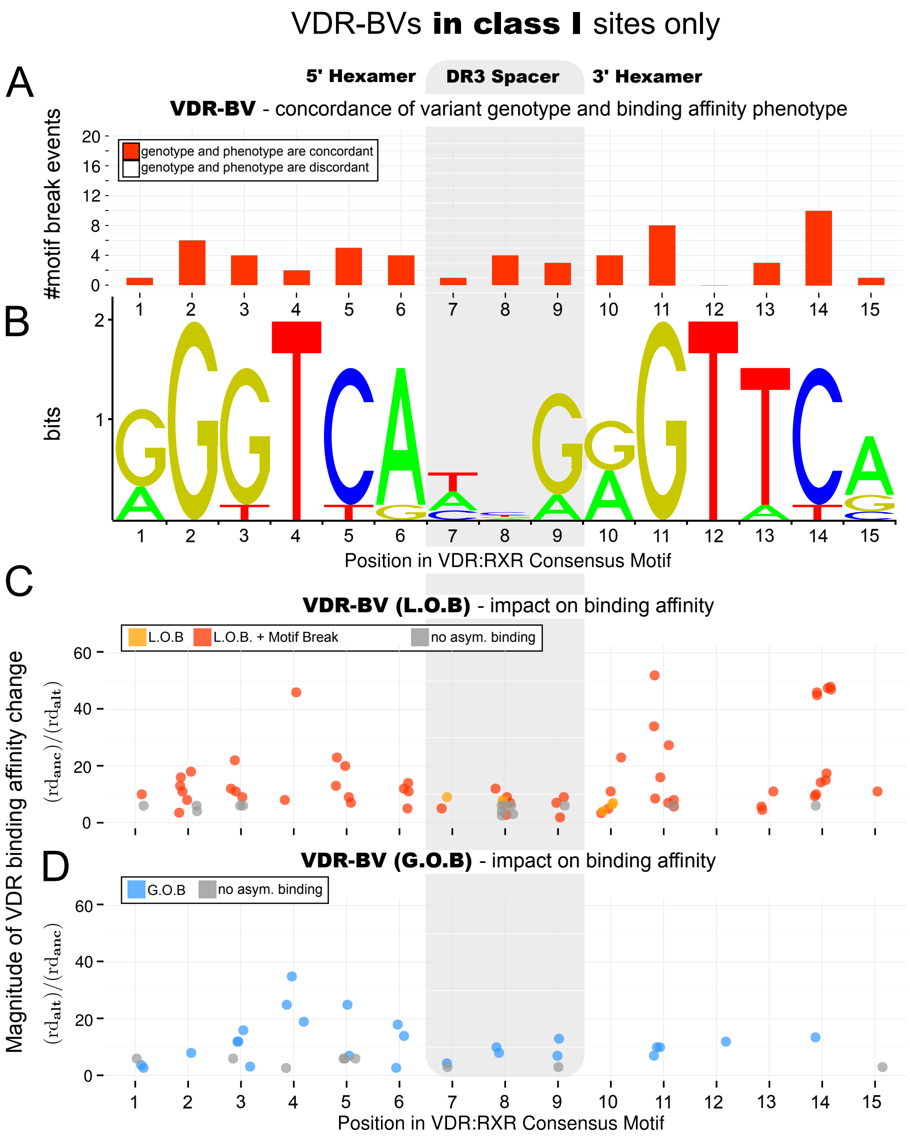

Figure S18: Class I RXR::VDR motifs — effects of binding variation on VDR binding affinity. Genome-wide quantification of the effect of genetic variation on VDR binding, based on the analysis of VDR-BVs in CP_{o_3} binding peaks which hit the canonical RXR::VDR DR3 heterodimeric consensus motif (motif shown in panel **B**, JASPAR database, Mathelier et al. [2014]). The figure shows data relative to those VDR-BVs which hit an instance of the VDR motif at class *I* VDR ChIP-exo binding regions. **A**: Distribution across the RXR::VDR motif of VDR-BVs which cause significant motif disruption (significance assessed via FunSeq2 using TFMPvalue [Touzet and Varre, 2007], with default threshold $p < 4 \times 10^{-8}$) and quantification of the concordance between directionality of VDR PWM disruption and directionality of resulting VDR binding affinity variation. 56/56 (100%) VDR-rBVs in CP_{o_3} VDR peaks that significantly break the RXR::VDR motif predict the direction of VDR binding affinity change. **C,D**: Genome-wide quantification of the phenotypic effect of all VDR-BVs intersecting the RXR::VDR motif (including those which do not generate a motif break at the above significance level). The vertical axis (*Magnitude of VDR binding affinity change*) indicates the fold change of read depth of the ancestral versus the derived allele (panel **C**) or derived versus the ancestral allele (panel **D**). **C**: Impact on VDR binding affinity of Loss of Binding (LOB, orange dots) VDR-BVs and Loss of Binding VDR-BVs which test for significant RXR::VDR motif break (red dots). **D**: Impact on VDR binding affinity of Gain of Binding (GOB) VDR-BVs (blue dots). **C,D**: Grey dots indicate impact on binding affinity of those 1000 Genomes variants carried by the LCL samples which do not test for significant asymmetric binding (AlleleSeq [Rozowsky et al., 2011], FDR threshold = 0.1) and are not, therefore, VDR-BVs.

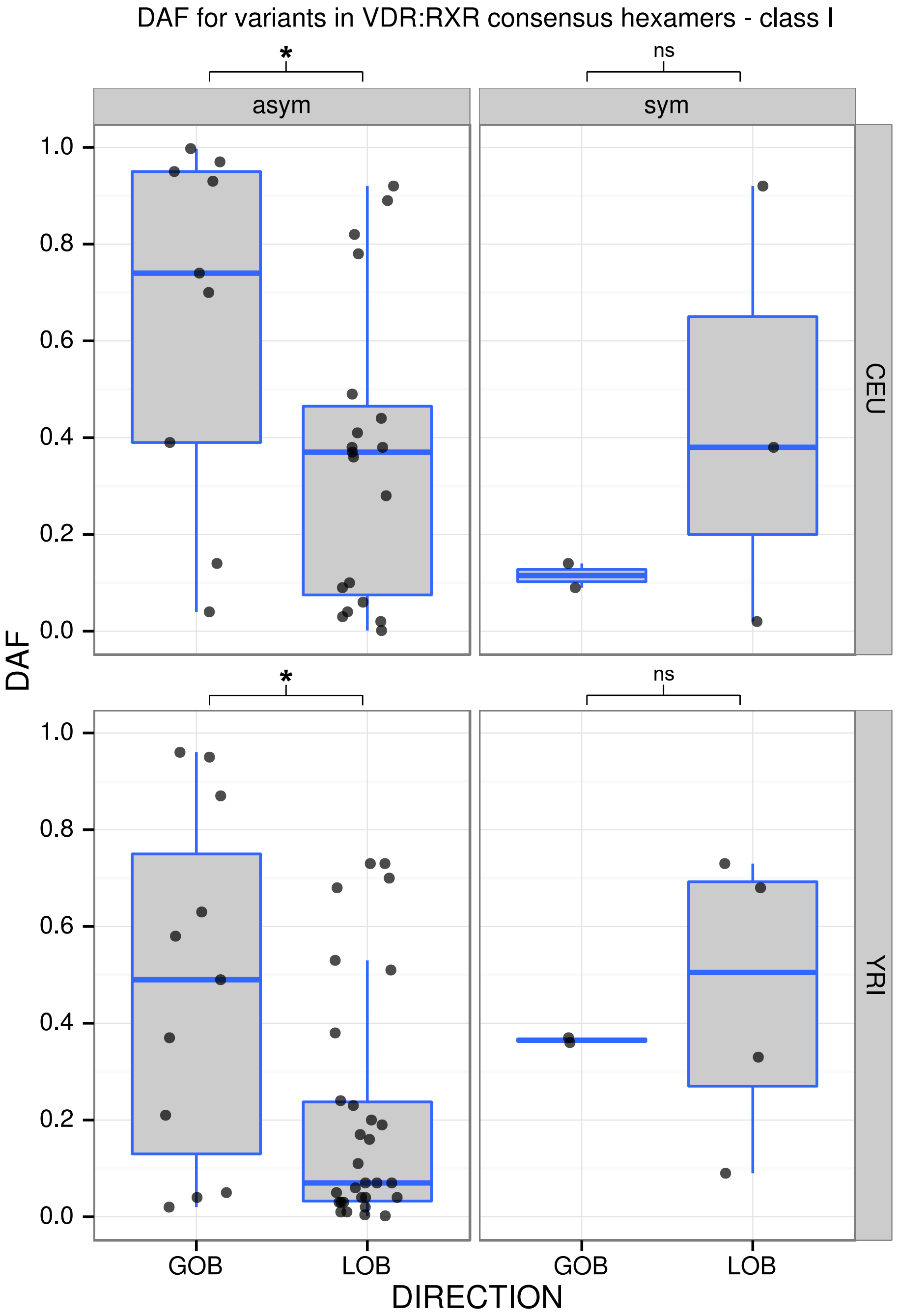

Figure S19: Evolutionary conservation of VDR-BVs at class I RXR::VDR motifs. The diagrams show distributions of the Derived Allele Frequency (DAF) for variants in RXR::VDR consensus motifs at class I binding sites only. DAF values are separated by ethnicity (top row: CEU; bottom row: YRI); variants are separated by their effect on VDR binding affinity (left column: VDR-BVs; right column: 1000genomes variants carried by the LCL samples which do not test for significant asymmetric binding and are not, therefore, VDR-BVs). Within each of the four quadrants, variants are split in two groups, based on their effect on VDR binding affinity direction (whether GOB or LOB). For all quadrants, only DAFs for variants hitting hexamer positions (i.e. hitting either the RXR recognition element at positions 1-6 or the VDR recognition element at positions 10-15) in class I RXR::VDR motif are shown. Asterisk indicates significance (Wilcoxon rank sum tests; $\alpha = 0.05$). *ns* = not significant.

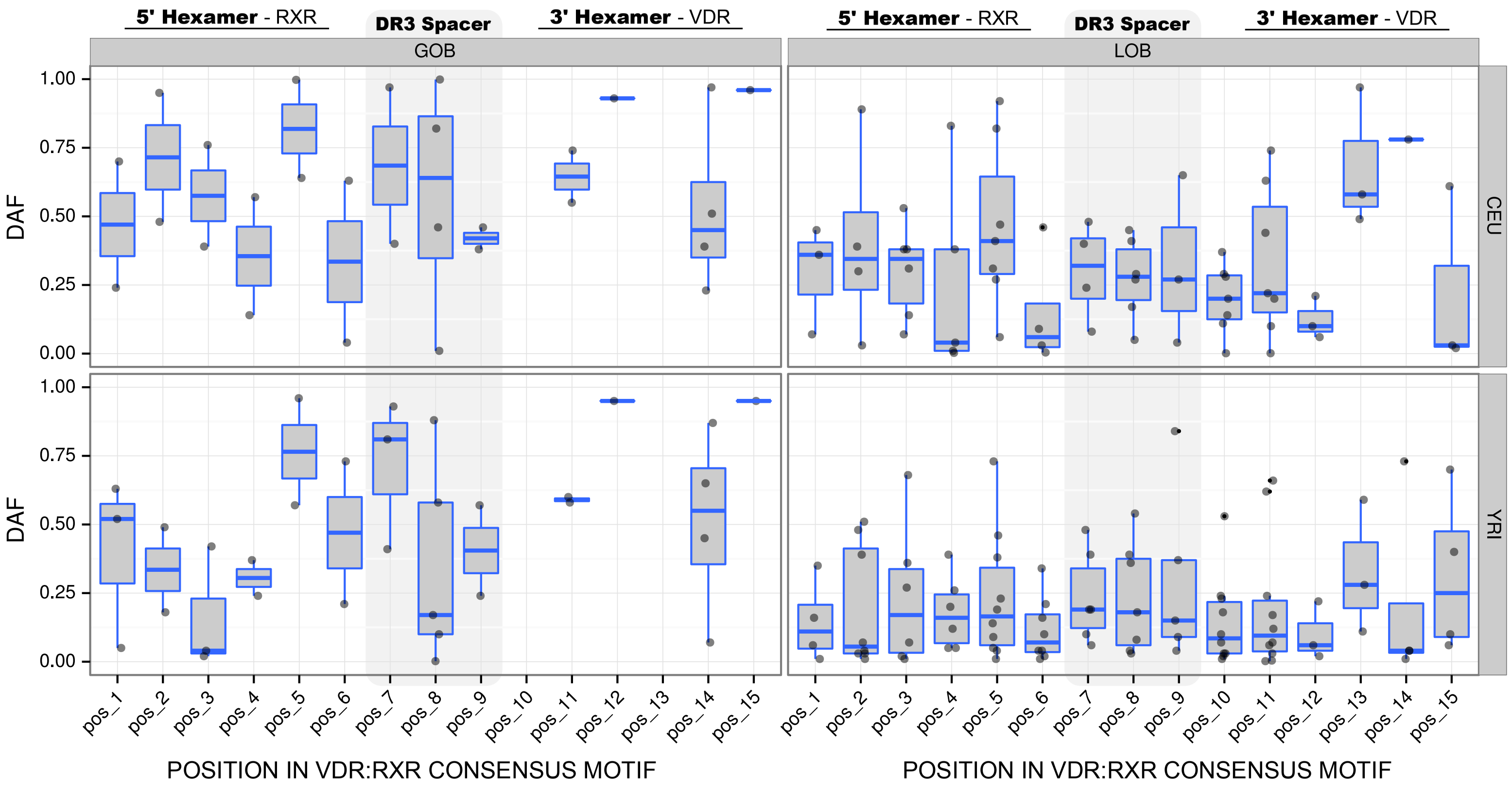

Figure S20: Distribution of DAFs for VDR-BVs in RXR::VDR motifs. Diagrams showing the distributions of the Derived Allele Frequency (DAF) for those VDR-BVs hitting a RXR::VDR consensus motifs. DAF values are separated by ethnicity (top row: CEU; bottom row: YRI); variants are split in two groups, based on their effect on VDR binding affinity direction (whether GOB or LOB).

References

Abecasis, G. R., Altshuler, D., Auton, A., Brooks, L. D., et al. (2010). A map of human genome variation from population-scale sequencing. *Nature*, 467(7319):1061–1073.

Abecasis, G. R., Auton, A., Brooks, L. D., DePristo, M. A., Durbin, R. M., Handsaker, R. E., Kang, H. M., Marth, G. T., McVean, G. A., Altshuler, D. M., et al. (2012). An integrated map of genetic variation from 1,092 human genomes. *Nature*, 491(7422):56–65.

Abyzov, A., Urban, A. E., Snyder, M., and Gerstein, M. (2011). CNVnator: an approach to discover, genotype, and characterize typical and atypical CNVs from family and population genome sequencing. *Genome Res.*, 21(6):974–984.

Andersson, R., Gebhard, C., Miguel-Escalada, I., Hoof, I., Bornholdt, J., Boyd, M., Chen, Y., Zhao, X., Schmidl, C., Suzuki, T., et al. (2014). An atlas of active enhancers across human cell types and tissues. *Nature*, 507(7493):455–461.

Bailey, T., Krajewski, P., Ladunga, I., Lefebvre, C., Li, Q., Liu, T., Madrigal, P., Taslim, C., and Zhang, J. (2013). Practical guidelines for the comprehensive analysis of ChIP-seq data. *PLoS Comput. Biol.*, 9(11):e1003326.

Bailey, T. L. (2011). DREME: motif discovery in transcription factor ChIP-seq data. *Bioinformatics*, 27(12):1653–1659.

Bailey, T. L., Boden, M., Buske, F. A., Frith, M., Grant, C. E., Clementi, L., Ren, J., Li, W. W., and Noble, W. S. (2009). MEME SUITE: tools for motif discovery and searching. *Nucleic Acids Res.*, 37(Web Server issue):W202–208.

Bailey, T. L. and Machanick, P. (2012). Inferring direct DNA binding from ChIP-seq. *Nucleic Acids Res.*, 40(17):e128.

Burton, P. R., Clayton, D. G., Cardon, L. R., Craddock, N., et al. (2007). Genome-wide association study of 14,000 cases of seven common diseases and 3,000 shared controls. *Nature*, 447(7145):661–678.

Carroll, T. S., Liang, Z., Salama, R., Stark, R., and de Santiago, I. (2014). Impact of artefact removal on ChIP quality metrics in ChIP-seq and ChIP-exo data. *Frontiers in Genetics*, 5(75).

Crooks, G. E., Hon, G., Chandonia, J. M., and Brenner, S. E. (2004). WebLogo: a sequence logo generator. *Genome Res.*, 14(6):1188–1190.

Degner, J. F., Marioni, J. C., Pai, A. A., Pickrell, J. K., Nkadori, E., Gilad, Y., and Pritchard, J. K. (2009). Effect of read-mapping biases on detecting allele-specific expression from RNA-sequencing data. *Bioinformatics*, 25(24):3207–3212.

Degner, J. F., Pai, A. A., Pique-Regi, R., et al. (2012). DNase I sensitivity QTLs are a major determinant of human expression variation. *Nature*, 482(7385):390–394.

Dunham, I., Kundaje, A., Aldred, S. F., Collins, P. J., Davis, C. A., Doyle, F., Epstein, C. B., Frietze, S., Harrow, J., Kaul, R., et al. (2012). An integrated encyclopedia of DNA elements in the human genome. *Nature*, 489(7414):57–74.

Eicher, J. D., Landowski, C., Stackhouse, B., Sloan, A., Chen, W., Jensen, N., Lien, J. P., Leslie, R., and Johnson, A. D. (2015). GRASP v2.0: an update on the Genome-Wide Repository of Associations between SNPs and phenotypes. *Nucleic Acids Res.*, 43(Database issue):799–804.

Feng, J., Liu, T., Qin, B., Zhang, Y., and Liu, X. S. (2012). Identifying ChIP-seq enrichment using MACS. *Nat Protoc*, 7(9):1728–1740.

Feng, X., Grossman, R., and Stein, L. (2011). PeakRanger: a cloud-enabled peak caller for ChIP-seq data. *BMC Bioinformatics*, 12:139.

Flicek, P., Amode, M. R., Barrell, D., Beal, K., et al. (2014). Ensembl 2014. *Nucleic Acids Research*, 42(D1):D749–D755.

Frazer, K. A., Ballinger, D. G., Cox, D. R., Hinds, D. A., et al. (2007). A second generation human haplotype map of over 3.1 million SNPs. *Nature*, 449(7164):851–861.

Fujita, P. A., Rhead, B., Zweig, A. S., Hinrichs, A. S., et al. (2011). The UCSC Genome Browser database: update 2011. *Nucleic Acids Research*, 39(suppl 1):D876–D882.

Gabriel, S. B., Schaffner, S. F., Nguyen, H., Moore, J. M., Roy, J., Blumenstiel, B., Higgins, J., DeFelice, M., Lochner, A., Faggart, M., et al. (2002). The structure of haplotype blocks in the human genome. *Science*, 296(5576):2225–2229.

Guo, Y., Mahony, S., and Gifford, D. K. (2012). High Resolution Genome Wide Binding Event Finding and Motif Discovery Reveals Transcription Factor Spatial Binding Constraints. *PLoS Comput Biol*, 8(8):e1002638.

Harrow, J., Frankish, A., Gonzalez, J. M., Tapanari, E., Diekhans, M., Kokocinski, F., Aken, B. L., Barrell, D., Zadissa, A., Searle, S., Barnes, I., Bignell, A., Boychenko, V., Hunt, T., Kay, M., Mukherjee, G., Rajan, J., Despacio-Reyes, G., Saunders, G., Steward, C., Harte, R., Lin, M., Howald, C., Tanzer, A., Derrien, T., Chrast, J., Walters, N., Balasubramanian, S., Pei, B., Tress, M., Rodriguez, J. M., Ezkurdia, I., van Baren, J., Brent, M., Haussler, D., Kellis, M., Valencia, A., Reymond, A., Gerstein, M., Guigó, R., and Hubbard, T. J. (2012). Gencode: The reference human genome annotation for the encode project. *Genome Research*, 22(9):1760–1774.

Hartmann, H., Guthohrlein, E. W., Siebert, M., Luehr, S., and Soding, J. (2013). P-value-based regulatory motif discovery using positional weight matrices. *Genome Res.*, 23(1):181–194.

Haussler, M., Whitfield, G., Kaneko, I., Haussler, C., Hsieh, D., Hsieh, J.-C., and Jurutka, P. (2013). Molecular Mechanisms of Vitamin D Action. *Calcified Tissue International*, 92(2):77–98.

Heger, A., Webber, C., Goodson, M., Ponting, C. P., and Lunter, G. (2013). GAT: a simulation framework for testing the association of genomic intervals. *Bioinformatics*, 29(16):2046–2048.

Heinz, S., Romanoski, C. E., Benner, C., Allison, K. A., Kaikkonen, M. U., Orozco, L. D., and Glass, C. K. (2013). Effect of natural genetic variation on enhancer selection and function. *Nature*, 503(7477):487–492.

Howie, B., Marchini, J., and Stephens, M. (2011). Genotype imputation with thousands of genomes. *G3 (Bethesda)*, 1(6):457–470.

Howie, B. N., Donnelly, P., and Marchini, J. (2009). A flexible and accurate genotype imputation method for the next generation of genome-wide association studies. *PLoS Genet.*, 5(6):e1000529.

(IMSGC), International Multiple Sclerosis Genetics Consortium (2013). Analysis of immune-related loci identifies 48 new susceptibility variants for multiple sclerosis. *Nat Genet*, 45(11):1353–1360.

Kent, W. J., Sugnet, C. W., Furey, T. S., Roskin, K. M., Pringle, T. H., Zahler, A. M., and Haussler, D. (2002). The human genome browser at UCSC. *Genome Res.*, 12(6):996–1006.

Kharchenko, P. V., Tolstorukov, M. Y., and Park, P. J. (2008). Design and analysis of ChIP-seq experiments for DNA-binding proteins. *Nat. Biotechnol.*, 26(12):1351–1359.

Khurana, E., Fu, Y., Colonna, V., Mu, X. J., Kang, H. M., Lappalainen, T., Sboner, A., Lochovsky, L., Chen, J., Harmanci, A., et al. (2013). Integrative annotation of variants from 1092 humans: application to cancer genomics. *Science*, 342(6154):1235587.

Kuhn, R. M., Haussler, D., and Kent, W. J. (2013). The UCSC genome browser and associated tools. *Brief. Bioinformatics*, 14(2):144–161.

Kundaje, A., Jung, L. Y., Kharchenko, P., Wold, B., Sidow, A., Batzoglou, S., and Park, P. (2013). Assessment of ChIP-seq data quality using cross-correlation analysis.

Landt, S. G., Marinov, G. K., Kundaje, A., Kheradpour, P., et al. (2012). ChIP-seq guidelines and practices of the ENCODE and modENCODE consortia. *Genome Research*, 22(9):1813–1831.

Langmead, B., Trapnell, C., Pop, M., and Salzberg, S. (2009). Ultrafast and memory-efficient alignment of short DNA sequences to the human genome. *Genome Biology*, 10(3):R25.

Lappalainen, T., Sammeth, M., Friedlander, M. R., 't Hoen, P. A., Monlong, J., Rivas, M. A., Gonzalez-Porta, M., Kurbatova, N., Griebel, T., Ferreira, P. G., et al. (2013). Transcriptome and genome sequencing uncovers functional variation in humans. *Nature*, 501(7468):506–511.

Li, H. and Durbin, R. (2009). Fast and accurate short read alignment with burrows-wheeler transform. *Bioinformatics*, 25(14):1754–1760.

Li, Q., Brown, J. B., Huang, H., and Bickel, P. J. (2011). Measuring reproducibility of high-throughput experiments. *The Annals of Applied Statistics*, 5(3):1752–1779.

Lunter, G. and Goodson, M. (2011). Stampy: a statistical algorithm for sensitive and fast mapping of Illumina sequence reads. *Genome Res.*, 21(6):936–939.

Ma, W., Noble, W. S., and Bailey, T. L. (2014). Motif-based analysis of large nucleotide data sets using MEME-ChIP. *Nat Protoc*, 9(6):1428–1450.

Marchini, J. and Howie, B. (2010). Genotype imputation for genome-wide association studies. *Nat. Rev. Genet.*, 11(7):499–511.

Marchini, J., Howie, B., Myers, S., McVean, G., and Donnelly, P. (2007). A new multipoint method for genome-wide association studies by imputation of genotypes. *Nat Genet*, 39(7):906–913.

Marinov, G. K., Kundaje, A., Park, P. J., and Wold, B. J. (2014). Large-scale quality analysis of published ChIP-seq data. *G3 (Bethesda)*, 4(2):209–223.

Mathelier, A., Zhao, X., Zhang, A. W., Parcy, F., Worsley-Hunt, R., Arenillas, D. J., Buchman, S., Chen, C. Y., Chou, A., Ienasescu, H., et al. (2014). JASPAR 2014: an extensively expanded and updated open-access database of transcription factor binding profiles. *Nucleic Acids Res.*, 42(Database issue):D142–147.

McDaniell, R., Lee, B.-K., Song, L., Liu, Z., Boyle, A. P., Erdos, M. R., Scott, L. J., Morken, M. A., Kucera, K. S., Battenhouse, A., et al. (2010). Heritable Individual-Specific and Allele-Specific Chromatin Signatures in Humans. *Science*, 328(5975):235–239.

McLean, C. Y., Bristor, D., Hiller, M., Clarke, S. L., Schaar, B. T., Lowe, C. B., Wenger, A. M., and Bejerano, G. (2010). GREAT improves functional interpretation of cis-regulatory regions. *Nat. Biotechnol.*, 28(5):495–501.

Meyer, L. R., Zweig, A. S., Hinrichs, A. S., Karolchik, D., et al. (2013). The UCSC Genome Browser database: extensions and updates 2013. *Nucleic Acids Research*, 41(D1):D64–D69.

Pavesi, G., Mereghetti, P., Zambelli, F., Stefani, M., Mauri, G., and Pesole, G. (2006). MoD Tools: regulatory motif discovery in nucleotide sequences from co-regulated or homologous genes. *Nucleic Acids Research*, 34(suppl 2):W566–W570.

Pickrell, J. K., Gaffney, D. J., Gilad, Y., and Pritchard, J. K. (2011). False positive peaks in ChIP-seq and other sequencing-based functional assays caused by unannotated high copy number regions. *Bioinformatics*, 27(15):2144–2146.

Pickrell, J. K., Marioni, J. C., Pai, A. A., Degner, J. F., et al. (2010). Understanding mechanisms underlying human gene expression variation with RNA sequencing. *Nature*, 464(7289):768–772.

Purcell, S. (2014). Plink 1.9b. `pngu.mgh.harvard.edu/purcell/plink/`. Accessed: 2014-12-10.

Quinlan, A. R. and Hall, I. M. (2010). BEDTools: a flexible suite of utilities for comparing genomic features. *Bioinformatics*, 26(6):841–842.

Quinodoz, M., Gobet, C., Naef, F., and Gustafson, K. B. (2014). Characteristic bimodal profiles of RNA polymerase II at thousands of active mammalian promoters. *Genome Biol.*, 15(6):R85.

R Core Team (2014). *R: A Language and Environment for Statistical Computing*. R Foundation for Statistical Computing, Vienna, Austria.

Ramachandran, P., Palidwor, G. A., Porter, C. J., and Perkins, T. J. (2013). MaSC: mappability-sensitive cross-correlation for estimating mean fragment length of single-end short-read sequencing data. *Bioinformatics*, 29(4):444–450.

Ramagopalan, S. V., Heger, A., Berlanga, A. J., Maugeri, N. J., Lincoln, M. R., Burrell, A., Handunnetthi, L., Handel, A. E., Disanto, G., Orton, S.-M., et al. (2010). A ChIP-seq defined genome-wide map of vitamin D receptor binding: Associations with disease and evolution. *Genome Research*, 20(10):1352–1360.

Rhee, H. S. and Pugh, B. F. (2011). Comprehensive Genome-wide Protein-DNA Interactions Detected at Single-Nucleotide Resolution. *Cell*, 147(6):1408–1419.

Robinson, M. and Oshlack, A. (2010). A scaling normalization method for differential expression analysis of RNA-seq data. *Genome Biology*, 11(3):R25.

Ross-Innes, C. S., Stark, R., Teschendorff, A. E., Holmes, K. A., Ali, H. R., Dunning, M. J., Brown, G. D., Gojis, O., Ellis, I. O., Green, A. R., Ali, S., Chin, S.-F., Palmieri, C., Caldas, C., and Carroll, J. S. (2012). Differential oestrogen receptor binding is associated with clinical outcome in breast cancer. *Nature*, 481(7381):389–393.

Rozowsky, J., Abyzov, A., Wang, J., Alves, P., Raha, D., Harmanci, A., Leng, J., Bjornson, R., Kong, Y., Kitabayashi, N., et al. (2011). AlleleSeq: analysis of allele-specific expression and binding in a network framework. *Molecular Systems Biology*, 7(1):–.

Scott, L. J., Mohlke, K. L., Bonnycastle, L. L., Willer, C. J., et al. (2007). A genome-wide association study of type 2 diabetes in Finns detects multiple susceptibility variants. *Science*, 316(5829):1341–1345.

Servin, B. and Stephens, M. (2007). Imputation-Based Analysis of Association Studies: Candidate Regions and Quantitative Traits. *PLoS Genet*, 3(7):e114.

Shen, L., Shao, N., Liu, X., and Nestler, E. (2014). ngs.plot: Quick mining and visualization of next-generation sequencing data by integrating genomic databases. *BMC Genomics*, 15(1):284.

Shen, L., Shao, N. Y., Liu, X., Maze, I., Feng, J., and Nestler, E. J. (2013). diffReps: detecting differential chromatin modification sites from ChIP-seq data with biological replicates. *PLoS ONE*, 8(6):e65598.

Siepel, A., Bejerano, G., Pedersen, J. S., Hinrichs, A. S., Hou, M., Rosenbloom, K., Clawson, H., Spieth, J., Hillier, L. W., Richards, S., Weinstock, G. M., Wilson, R. K., Gibbs, R. A., Kent, W. J., Miller, W., and Haussler, D. (2005). Evolutionarily conserved elements in vertebrate, insect, worm, and yeast genomes. *Genome Res.*, 15(8):1034–1050.

Stark, R. and Brown, G. (2011). DiffBind: differential binding analysis of ChIP-Seq peak data. R package.

Stephens, M. and Balding, D. J. (2009). Bayesian statistical methods for genetic association studies. *Nat. Rev. Genet.*, 10(10):681–690.

Touzet, H. and Varre, J. S. (2007). Efficient and accurate P-value computation for Position Weight Matrices. *Algorithms Mol Biol*, 2:15.

Wakefield, J. (2009). Bayes factors for genome-wide association studies: comparison with P-values. *Genet. Epidemiol.*, 33(1):79–86.

Welter, D., MacArthur, J., Morales, J., Burdett, T., Hall, P., Junkins, H., Klemm, A., Flicek, P., Manolio, T., Hindorff, L., and Parkinson, H. (2014). The NHGRI GWAS Catalog, a curated resource of SNP-trait associations. *Nucleic Acids Res.*, 42(Database issue):D1001–1006.

Yip, K., Cheng, C., Bhardwaj, N., Brown, J., Leng, J., Kundaje, A., Rozowsky, J., Birney, E., Bickel, P., Snyder, M., and Gerstein, M. (2012). Classification of human genomic regions based on experimentally determined binding sites of more than 100 transcription-related factors. *Genome Biology*, 13(9):R48.

Zambelli, F., Pesole, G., and Pavesi, G. (2013). PscanChIP: Finding over-represented transcription factor-binding site motifs and their correlations in sequences from ChIP-Seq experiments. *Nucleic Acids Res.*, 41(Web Server issue):W535–543.

Zapata, C. (2000). The D' measure of overall gametic disequilibrium between pairs of multiallelic loci. *Evolution*, 54(5):1809–1812.

Zhang, Y., Liu, T., Meyer, C., Eeckhoute, J., Johnson, D., Bernstein, B., Nusbaum, C., Myers, R., Brown, M., Li, W., and Liu, X. S. (2008). Model-based Analysis of ChIP-Seq (MACS). *Genome Biology*, 9(9):R137.
